# *Prdm16* mutation determines sex-specific cardiac metabolism and identifies two novel cardiac metabolic regulators

**DOI:** 10.1093/cvr/cvad154

**Published:** 2023-10-16

**Authors:** Jirko Kühnisch, Simon Theisen, Josephine Dartsch, Raphaela Fritsche-Guenther, Marieluise Kirchner, Benedikt Obermayer, Anna Bauer, Anne-Karin Kahlert, Michael Rothe, Dieter Beule, Arnd Heuser, Philipp Mertins, Jennifer A Kirwan, Nikolaus Berndt, Calum A MacRae, Norbert Hubner, Sabine Klaassen

**Affiliations:** Charité – Universitätsmedizin Berlin, corporate member of Freie Universität Berlin and Humboldt-Universität zu Berlin, Berlin, Germany; Experimental and Clinical Research Center, Lindenberger Weg 80, 13125 Berlin, Germany; Max Delbrück Center for Molecular Medicine in the Helmholtz Association (MDC), Berlin, Germany; DZHK (German Centre for Cardiovascular Research), partner site Berlin, Berlin, Germany; Charité – Universitätsmedizin Berlin, corporate member of Freie Universität Berlin and Humboldt-Universität zu Berlin, Berlin, Germany; Experimental and Clinical Research Center, Lindenberger Weg 80, 13125 Berlin, Germany; Max Delbrück Center for Molecular Medicine in the Helmholtz Association (MDC), Berlin, Germany; DZHK (German Centre for Cardiovascular Research), partner site Berlin, Berlin, Germany; Charité – Universitätsmedizin Berlin, corporate member of Freie Universität Berlin and Humboldt-Universität zu Berlin, Berlin, Germany; Experimental and Clinical Research Center, Lindenberger Weg 80, 13125 Berlin, Germany; Max Delbrück Center for Molecular Medicine in the Helmholtz Association (MDC), Berlin, Germany; BIH Metabolomics Platform, Berlin Institute of Health (BIH) at Charité - Universitätsmedizin Berlin, Berlin, Germany; Max-Delbrück-Center for Molecular Medicine in the Helmholtz Association (MDC), Proteomics Platform, Berlin, Germany; Berlin Institute of Health (BIH) at Charité - Universitätsmedizin Berlin, Berlin, Germany; Core Unit Bioinformatics, Berlin Institute of Health at Charité - Universitätsmedizin Berlin, Berlin, Germany; BIH Metabolomics Platform, Berlin Institute of Health (BIH) at Charité - Universitätsmedizin Berlin, Berlin, Germany; Department of Congenital Heart Disease and Pediatric Cardiology, University Hospital of Schleswig-Holstein, Kiel, Germany; DZHK German Center for Cardiovascular Research, partner site Hamburg/Kiel/Lübeck, Germany; Institute of Immunology and Genetics, Kaiserslautern, Germany; Lipidomix GmbH, Berlin, Germany; Max Delbrück Center for Molecular Medicine in the Helmholtz Association (MDC), Berlin, Germany; Core Unit Bioinformatics, Berlin Institute of Health at Charité - Universitätsmedizin Berlin, Berlin, Germany; Max Delbrück Center for Molecular Medicine in the Helmholtz Association (MDC), Berlin, Germany; Max-Delbrück-Center for Molecular Medicine in the Helmholtz Association (MDC), Proteomics Platform, Berlin, Germany; Berlin Institute of Health (BIH) at Charité - Universitätsmedizin Berlin, Berlin, Germany; BIH Metabolomics Platform, Berlin Institute of Health (BIH) at Charité - Universitätsmedizin Berlin, Berlin, Germany; Charité – Universitätsmedizin Berlin, corporate member of Freie Universität Berlin and Humboldt-Universität zu Berlin, Berlin, Germany; Institute of Computer-assisted Cardiovascular Medicine, Deutsches Herzzentrum der Charité (DHZC), Berlin, Germany; Department of Molecular Toxicology, German Institute of Human Nutrition Potsdam—Rehbruecke (DIfE), Nuthetal, Germany; Harvard Medical School and Cardiovascular Division, Department of Medicine, Brigham and Women’s Hospital, Boston, USA; Max Delbrück Center for Molecular Medicine in the Helmholtz Association (MDC), Berlin, Germany; DZHK (German Centre for Cardiovascular Research), partner site Berlin, Berlin, Germany; Charité – Universitätsmedizin Berlin, corporate member of Freie Universität Berlin and Humboldt-Universität zu Berlin, Berlin, Germany; Experimental and Clinical Research Center, Lindenberger Weg 80, 13125 Berlin, Germany; Max Delbrück Center for Molecular Medicine in the Helmholtz Association (MDC), Berlin, Germany; DZHK (German Centre for Cardiovascular Research), partner site Berlin, Berlin, Germany; Department of Pediatric Cardiology, Deutsches Herzzentrum der Charité (DHZC), Berlin, Germany

**Keywords:** Cardiomyopathy, Prdm16, Mutation, Metabolism

## Abstract

**Aims:**

Mutation of the *PRDM16* gene causes human dilated and non-compaction cardiomyopathy. The PRDM16 protein is a transcriptional regulator that affects cardiac development via Tbx5 and Hand1, thus regulating myocardial structure. The biallelic inactivation of *Prdm16* induces severe cardiac dysfunction with post-natal lethality and hypertrophy in mice. The early pathological events that occur upon *Prdm16* inactivation have not been explored.

**Methods and results:**

This study performed in-depth pathophysiological and molecular analyses of male and female *Prdm16^csp1/wt^* mice that carry systemic, monoallelic *Prdm16* gene inactivation. We systematically assessed early molecular changes through transcriptomics, proteomics, and metabolomics. Kinetic modelling of cardiac metabolism was performed *in silico* with CARDIOKIN. *Prdm16^csp1/wt^* mice are viable up to 8 months, develop hypoplastic hearts, and diminished systolic performance that is more pronounced in female mice. *Prdm16^csp1/wt^* cardiac tissue of both sexes showed reductions in metabolites associated with amino acid as well as glycerol metabolism, glycolysis, and the tricarboxylic acid cycle. *Prdm16^csp1/wt^* cardiac tissue revealed diminished glutathione (GSH) and increased inosine monophosphate (IMP) levels indicating oxidative stress and a dysregulated energetics, respectively. An accumulation of triacylglycerides exclusively in male *Prdm16^csp1/wt^* hearts suggests a sex-specific metabolic adaptation. Metabolic modelling using CARDIOKIN identified a reduction in fatty acid utilization in males as well as lower glucose utilization in female *Prdm16^csp1/wt^* cardiac tissue. On the level of transcripts and protein expression, *Prdm16^csp1/wt^* hearts demonstrate an up-regulation of pyridine nucleotide-disulphide oxidoreductase domain 2 (Pyroxd2) and the transcriptional regulator pre-B-cell leukaemia transcription factor interacting protein 1 (Pbxip1). The strongest concordant transcriptional up-regulation was detected for *Prdm16* itself, probably through an autoregulatory mechanism.

**Conclusions:**

Monoallelic, global *Prdm16* mutation diminishes cardiac performance in *Prdm16^csp1/wt^* mice. Metabolic alterations and transcriptional dysregulation in *Prdm16^csp1/wt^* affect cardiac tissue. Female *Prdm16^csp1/wt^* mice develop a more pronounced phenotype, indicating sexual dimorphism at this early pathological window. This study suggests that metabolic dysregulation is an early event in the *PRDM16* associated cardiac pathology.

## Graphical Abstract

**Graphical Abstract:**
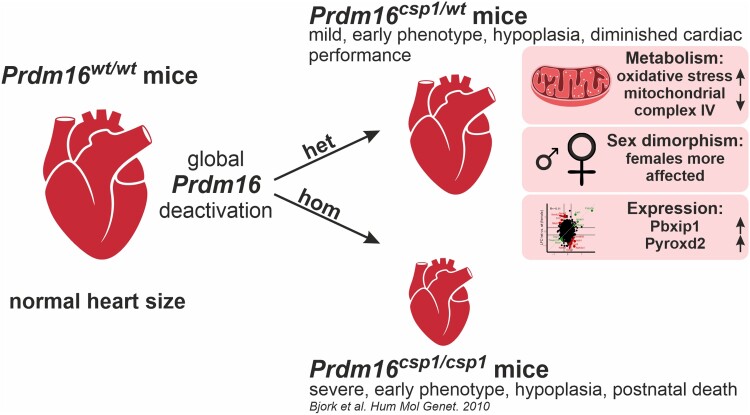

**Time of primary review: 47 days**

## 1. Introduction

Primary, genetically determined cardiomyopathies comprise a group of heterogenous cardiac diseases that eventually result in heart failure or arrhythmia. Approximately 100 genes have been linked to cardiomyopathy. These most frequently affect the sarcomere, Z-disc, mitochondria, or the regulation of ion channels.^1,2^ Transcription and splicing may be directly or indirectly disturbed in cardiomyopathies. The PR/SET domain 16 (PRDM16) protein directly affects transcriptional regulation.^3^ Mutation of the *PRDM16* gene causes dilated (DCM) and left ventricular non-compaction cardiomyopathy (LVNC) in patients.^4–7^ Recently, rare variant association analysis linked LVNC to PRDM16 protein-truncating variants.^8^ These findings suggest that *PRDM16* is a genetic factor that is critical for cardiac function, causing either monogenic cardiomyopathy or other myocardial phenotypes.

The homozygous germline inactivation of *Prdm16* in *Prdm16^csp1/csp1^* mice results in a complex phenotype involving several organs, cardiac hypoplasia, and early post-natal lethality.^9^ More recently, the role of *Prdm16* in myocardial development and LVNC was assessed after a homozygous, cardiac-specific inactivation of *Prdm16* in *Xmlc2Cre;Prdm16^flox/flox^* mice.^10^ These mice develop cardiac dysfunction and die prematurely, before post-natal Day 7. Previous work showed that during cardiac development, Prdm16 cooperates with the transcription factors T-box 5 (Tbx5) and heart and neural crest derivatives expressed 1 (Hand1) to promote gene programs required for myocardial growth and compaction.^10^ Prdm16 also suppresses neural gene expression.^10^ Consequently, its inactivation in *Xmlc2Cre;Prdm16^flox/flox^* mice leads to biventricular hypertrabeculation and left ventricular dilatation.^10^ This established that *Prdm16* is critical in orchestrating the transcriptional circuits that determine myocardial maturation during embryonic cardiac development and post-natal function.

PRDM16 had originally been characterized as a determinant for the differentiation, homeostasis, and function of brown/beige adipocytes.^11^ PRDM16 induces gene programs for the development of brown adipocytes, represses muscle/white adipocyte-specific genes, induces adaptive thermogenesis, and increases energy expenditure.^12–15^ On a molecular level, these effects are facilitated through its physical interactions with and co-ordination of key transcription factors such as the peroxisome proliferator-activated receptors alpha and gamma (PPARA, PPARG), mediator complex subunit 1 (MED1), and CCAAT enhancer-binding protein delta (CEBPD).^12,16,17^ Of note, a significant number of these PRDM16 associated proteins serve as critical regulators of fatty acid (FA) metabolism, glucose utilization, and/or cellular respiration.

This study tests the hypothesis that PRDM16 orchestrates cardiac metabolism and has potential roles beyond the known transcriptional functions in cardiac development and homeostasis. We characterize the heart of heterozygous *Prdm16^csp1/wt^* mice in depth to assess the impact of monoallelic germline *Prdm16* inactivation, the situation seen in patients with *PRDM16* associated cardiomyopathy. We explore early molecular events upon *Prdm16* inactivation and establish a pre-clinical animal model. We find that *Prdm16^csp1/wt^* mice show diminished cardiac performance, altered body composition, but normal survival. On the molecular level, cardiac cells exhibit changes in metabolism, redox balance, and FA/glucose utilization. Overall, this study establishes heterozygous *Prdm16^csp1/wt^* mice as a model for early molecular pathomechanistic events in the development of the *PRDM16* associated cardiomyopathy.

## 2. Methods

A detailed material and methods section is provided in the Supplementary material online, *Material*. The high-throughput sequencing data and proteome data have been made publicly available at the Gene Expression Omnibus (GEO accessionNo. GSE236791) and the Proteomics Identification Database (PRIDE accession No. PXD043601). Other data and study materials are available from the corresponding authors on reasonable request. Primer and antibodies used in this study are available (see Supplementary material online, *Tables SI* and *SII* in the Data Supplement). The *Prdm16^csp1/wt^* mice (FVB.C-*Prdm16^csp1^*/J) were received from Jackson Laboratories, USA (JAX stock #013100). The FVB.C-*Prdm16^csp1^*/J strain was originally established by *N*-ethyl-*N*-nitrosourea (ENU) mutagenesis inducing a missense C>A mutation at the intronic acceptor splice site of exon 7.^9^ Maintenance, physiological analysis, and organ collection of *Prdm16^csp1/wt^* mice were approved by the Landesamt für Gesundheit und Soziales Berlin (LAGeSo), Germany (G0070/17). Mice were euthanized by isoflurane inhalation and subsequent cervical dislocation. Organs were collected, immediately frozen in liquid nitrogen, and stored at −80°C. All experiments in this work involving the use of animals were monitored and approved by the LAGeSo and the MDC animal facility, according to the German law and the guidelines from Directive 2010/63/EU of the European Parliament.

## 3. Results

### 3.1 Germline, heterozygous inactivation of *Prdm16* induces mild cardiac dysfunction

**Figure 1:**
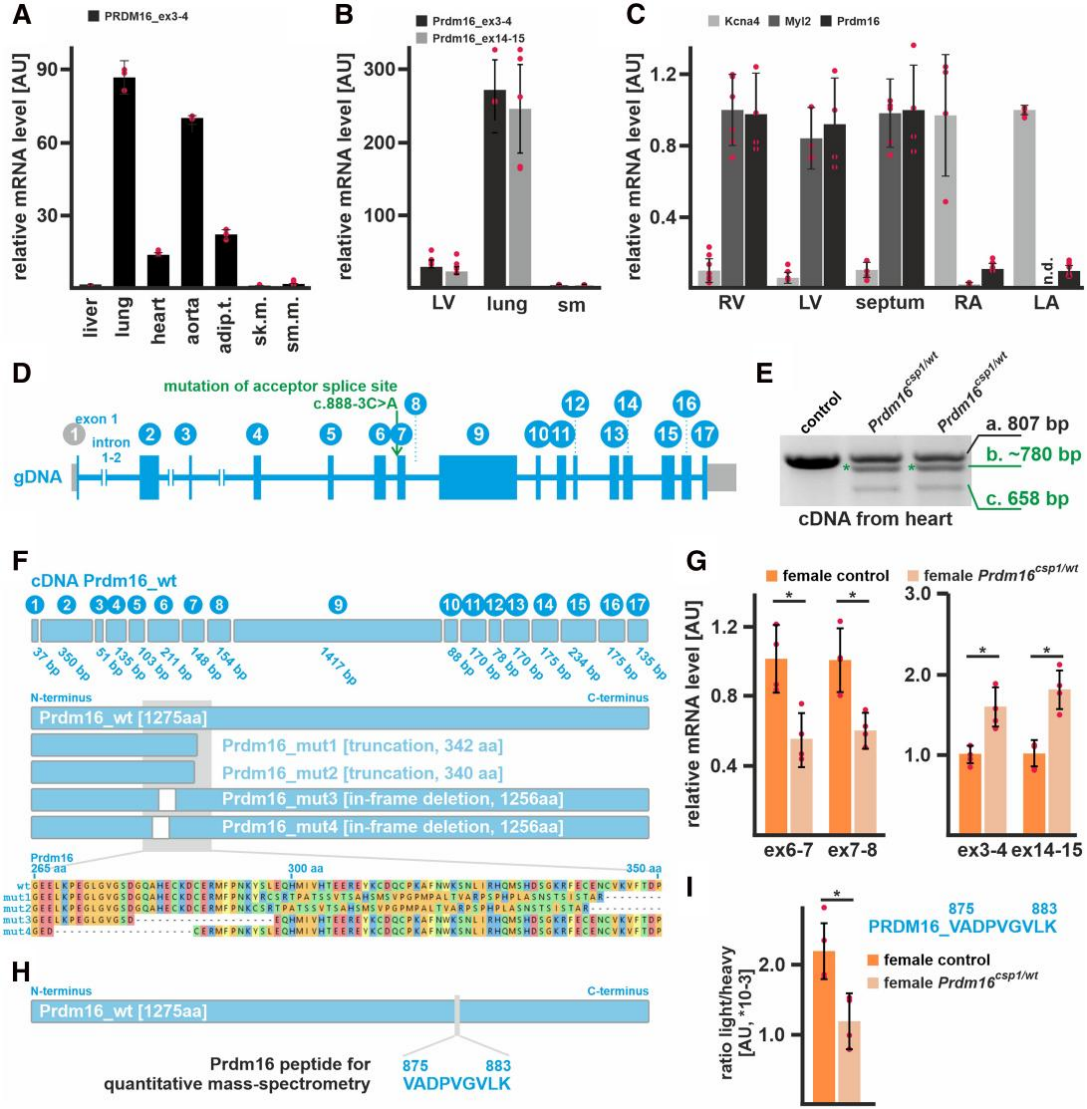
Expression of *Prdm16* in the heart of *Prdm16^csp1/wt^* mice. (*A*) Expression analysis reveals high *PRDM16* transcript levels in human lung, aorta, adipose tissue (adip.t.), and heart (technical replicate *n* = 3). Liver, skeletal muscle (sk.m.), and smooth muscle (sm.m.) show low *PRDM16* expression. (*B*) *Prdm16* is highly expressed in murine lung tissue, and shows robust levels in the left ventricle (LV) but low expression in skeletal muscle (sm) (*n* = 3). (*C*) *Prdm16* is robustly expressed in LV, right ventricle (RV), and the septum. The left (LA) and right atrium (RA) does not show considerable *Prdm16* expression. *Kcna4* and *Myl2* transcripts demonstrate atrial and ventricular tissue origin (*n* = 3). (*D*) The murine *Prdm16* gene comprises 17 exons, and *Prdm16^csp1/wt^* mice carry the point mutation c.888-3C>A affecting the acceptor splice site of intron_6–7. (*E*) PCR genotyping with heart cDNA from heterozygous *Prdm16^csp1/wt^* mice generates three products as follows: (i) wild-type fragment (807 bp), (ii) unknown mutant product (∼780 bp, *), and (iii) mutant product without exon 7 (658 bp). (*F*) The wild-type Prdm16 protein is 1275 amino acids (aa) long. Targeted high-throughput sequencing of the different PCR products identifies four main Prdm16 mutant proteins in *Prdm16^csp1/wt^* mice including truncation (mut1, mut2) and in-frame deletion (mut3, mut4) variants (see Supplementary material online, *Figures SI* and *SII* in the Data Supplement, for detailed results, see Data Supplement). (*G*) Quantitative detection of *Prdm16* transcript levels with qPCR detectors targeting the exon 7 deletion region shows approximately 40–50% reduced expression. In contrast, PCR detectors targeting exon3–4 and exon14–15 identify increased *Prdm16* levels. Analysis was performed on female *Prdm16^csp1/wt^* heart tissue (*n* = 3). (*H*) Detection of Prdm16 protein was achieved by PRM (parallel-reaction monitoring) using the Prdm16 derived peptide V875-K883. (*I*) Quantitation of Prdm16 peptide in control and *Prdm16^csp1/wt^* lung tissue (*n* = 4). The ratio of endogenous (light) and standard spike-in (heavy) peptide is shown. Statistical analysis was performed with unpaired *t*-test (*P* < 0.05).

In RNA preparations from human tissues, *PRDM16* transcripts are most abundant in lung, followed by aorta, adipose tissues, and heart (*Figure 1A*). Consistent with this, RNA extracts from murine tissue show approximately 10-fold higher *Prdm16* expression in lung compared to heart (*Figure 1B*). Comparing regions of the heart, *Prdm16* is abundantly expressed in the right ventricle (RV), left ventricle (LV), and septum but not in the atria (*Figure 1C*). Potassium voltage-gated channel member 4 (*Kcna4*) and myosin light chain 2 (*Myl2*) confirmed atrial and ventricular origin, respectively. To assess the physiological and molecular impact of *Prdm16* in the heart, we analysed heterozygous FVB.C-*Prdm16^csp1^*/J mice (*Prdm16^csp1/wt^*) rather than studying the effects of biallelic gene inactivation, which would not reflect the clinical situation.^9^ PCR genotyping and Sanger sequencing confirmed the presence of the c.888-3C>A (ENSMUSG00000039410) variant on the DNA level (*Figure 1D*, Supplementary material online, *Figure SIA* and *B* in the Data Supplement). To further validate the impact of the *Prdm16* acceptor splice site variant c.888-3C>A (ENSMUST00000030902.12) at the transcriptional level, we performed PCR and targeted high-throughput sequencing of total RNA isolated from different *Prdm16^wt/wt^* and *Prdm16^csp1/wt^* tissues. These analyses suggest that the *Prdm16* acceptor splice site variant c.888-3C>A affects mRNA splicing and produces several splice products. The most abundant splice products truncate Prdm16 proteins after approximately 340 amino acids (*Figure 1E* and *F*, for detailed results, see Data Supplement).

Next, we examined the expression of mutant *Prdm16* transcripts in the heart. *Prdm16* mutant transcripts are expressed at ∼40% lower levels than controls, as established using primer sets that target the mutated exon6–8 region (*Figure 1G*, Supplementary material online, *Figure SIF* in the Data Supplement). In contrast, primer sets measuring the overall *Prdm16* transcript level (exon3–4, exon14–15) detect increased expression in *Prdm16^csp1/wt^* hearts. This suggests that *Prdm16* inactivation due to the c.888-3C>A variant up-regulates overall *Prdm16* mRNA expression. Levels of Prdm16 protein were assessed with a mass spectrometry-based targeted proteomics approach, measuring and quantifying the abundance of the peptide Prdm16_V875-K883 (*Figure 1H*). As expected, a ∼50% reduction of the Prdm16_V875-K883 level was observed in *Prdm16^csp1/wt^* lung compared to controls (*Figure 1I*). In cardiac tissue, the Prdm16_V875-K883 detection limit was not reached.

**Figure 2:**
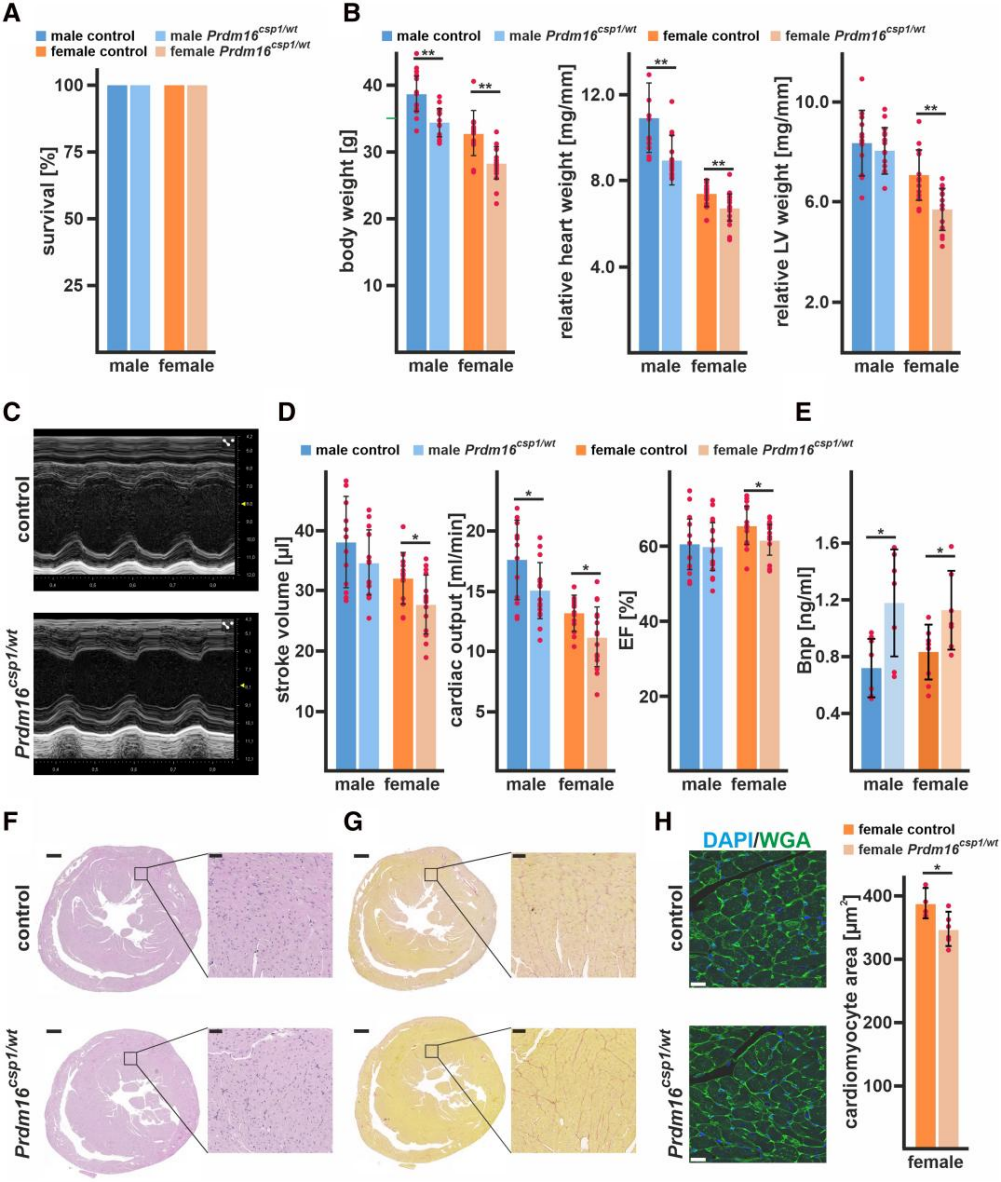
Diminished cardiac performance after *Prdm16* inactivation. (*A*) Survival of male and female *Prdm16^csp1/wt^* mice is normal until the age of 8 month (*n* > 13 each group). (*B*) Eight-month-old *Prdm16^csp1/wt^* mice of both sexes have a reduced absolute body weight. Normalization against tibia length demonstrated a diminished relative heart and LV weight in *Prdm16^csp1/wt^* mutants (*n* > 13 each group). (*C*) M-mode images illustrate the heart phenotype in *Prdm16^csp1/wt^* mice as assessed with echocardiography. (*D*) At the age of 8 months, *Prdm16^csp1/wt^* mice of both sexes have diminished cardiac function as illustrated by stroke volume, cardiac output, and ejection fraction (EF) (*n* > 12 each group). Full echocardiography data are available in Supplementary material online, *Tables SIV*–*SVI* in the Data Supplement. (*E*) Plasma brain natriuretic peptide (Bnp) levels were determined with ELISA (*n* > 5 each group). (*F*) Histological analysis with H&E staining reveals normal tissue organization in female *Prdm16^csp1/wt^* hearts. Scale bars in *E* and *F* are 500 µm (full heart) and 50 µm (inset). (*G*) Histological staining for fibrosis with Picro-Sirius red is negative in female *Prdm16^csp1/wt^* heart tissue. (*H*) The cell area of female heart muscle (cross sections) stained with wheat germ agglutinin (WGA) is significantly reduced (*n* = 5, each *n* includes >300 cells). Nuclei were stained with DAPI. Scale bar is 20 µm. Statistical analysis was performed with unpaired *t*-test (*P* < 0.05).

The survival of *Prdm16^csp1/wt^* mice is normal until 8 months (*Figure 2A*). Male and female *Prdm16^csp1/wt^* mice exhibit body weight reduction by ∼11% and ∼20%, respectively (*Figure 2B*, Supplementary material online, *Table SIII* in the Data Supplement). In female *Prdm16^csp1/wt^* mice, the drop in body weight was associated with a reduction in fat content (∼14%) and an increase in relative muscle tissue content (∼8%). *Prdm16^csp1/wt^* mice do not show any other abnormality or phenotype such as cleft palate or respiratory problems. Until the age of 8 months, we did not observe premature deaths. Both the absolute and relative heart weights are diminished in male and female *Prdm16^csp1/wt^* mice (*Figure 2B*). We used transthoracic echocardiography to characterize the cardiac physiology of *Prdm16^csp1/wt^* mice. Echocardiography indicated mild cardiac hypoplasia and reduced systolic functional parameters such as stroke volume, cardiac output, and ejection fraction (EF) (*Figure 2C* and *D*, Supplementary material online, *Tables SIV*–*SVI* in the Data Supplement) (original echocardiography images Supplementary material online, *Figure SIII* in the Data Supplement). Other cardiac parameters such as heart rate, systolic/diastolic blood pressure, or electrophysiology are unaffected in *Prdm16^csp1/wt^* mice. We found an elevation of plasma levels of brain natriuretic peptide (Bnp) in both sexes, indicating cardiomyocyte stretch with impaired systolic and possibly diastolic LV dysfunction (*Figure 2E*). Cardiac tissue organization, as assessed by histology and haematoxylin/eosin staining, appeared normal in *Prdm16^csp1/wt^* hearts (*Figure 2F*). Fibrosis was not detected, either by Picro-Sirius red staining or immunostaining of collagen 1 (Col1) and alpha smooth muscle actin (αSma) (*Figure 2G*, Supplementary material online, *Figure SIII* in the Data Supplement). Further analyses of *Prdm16^csp1/wt^* cardiac tissue with electron microscopy, morphometry, quantitative PCR, and immunostaining identified no abnormalities in sarcomeres (see Supplementary material online, *Figure SIV* in the Data Supplement, for detailed results, see Data Supplement). The relative mitochondrial area in heart tissue was unaffected. We assessed the cardiomyocyte area histomorphometrically using paraffin embedded heart tissue sections of comparable cross-sectional level after wheat germ agglutinin (WGA) staining. Cardiomyocytes of female *Prdm16^csp1/wt^* mice demonstrate a significant reduction of cross-sectional area explaining reduced myocardial mass (*Figure 2H*). Normal viability, myocardial hypoplasia, and diminished cardiac performance due to monoallelic *Prdm16* inactivation suggest that *Prdm16^csp1/wt^* mice are a suitable model to explore early pathophysiological changes in *PRDM16* associated cardiomyopathy.

### 3.2 Moderate transcriptional dysregulation in *Prdm16^csp1/wt^* hearts

**Figure 3:**
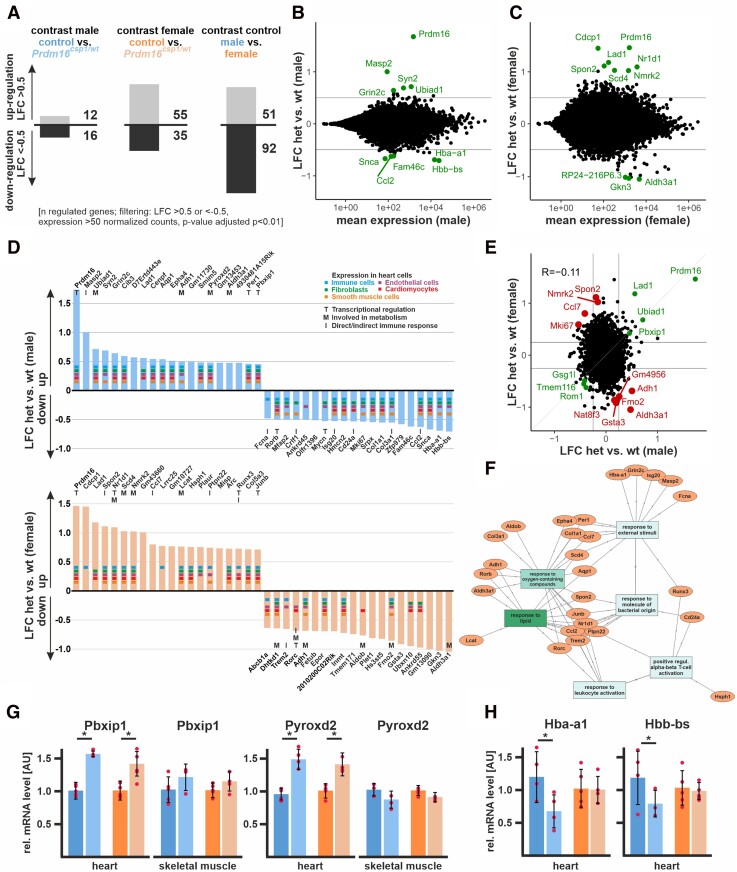
Transcriptome analysis of *Prdm16^csp1/wt^* heart tissue. (*A*) Transcriptome analysis with RNAseq of *Prdm16^csp1/wt^* LV shows in male 28 and in female 90 differentially regulated genes compared to controls. Analysis of male vs. female control hearts identifies 143 differentially regulated transcripts. Analysed biological replicates are *n* = 4–6. (*B* and *C*) Bioinformatic filtering ranked the normalized log2 cpk according the absolute log2 fold change (LFC) after elimination of regulated targets with an adjusted *P*-value (padj) < 10^−2^. Scatterplots show the LFC against mean expression for the contrast *Prdm16^csp1/wt^* (het) vs. controls (wt) in male (*B*) or female (*C*) heart. The top 10 differentially regulated genes (adj. *P* < 0.01) are highlighted and labelled. (*D*) Summary of top 20 up- and down-regulated genes in male (upper panel) and female (lower panel) hearts. Expression of selected genes in cardiac cell types is annotated according to the colour code. Functional association with transcription (T), metabolism (M), and immune response (I) is shown for relevant genes. (*E*) Scatterplot shows the LFC detected in *A* and *B* against each other. Genes with concordant changes (abs LFC > 0.25) are highlighted in green, and genes with discordant changes (top 10; adj. *P* < 0.01) are highlighted in red. The overall correlation is *R* = −0.11 (*n* = 21 276). (*F*) Gene ontology (GO) network was constructed with GOnet^18^ by using the male and female TOP20 up- and down-regulated genes in *Prdm16^csp1/wt^* hearts. GO terms *response to lipids* and *response to oxygen-containing compounds* show strongest association. (*G*) Validation of RNAseq expression data for *Pbxip1* and *Pyroxd2* was performed with qPCR using whole RNA extracts from heart and skeletal muscle. *Pbxip1* and *Pyroxd2* expression is increased in hearts from *Prdm16^csp1/wt^* mice of both sexes. (*H*) *Hba-a1* and *Hbb-bs* expression is diminished in male *Prdm16^csp1/wt^* hearts.

Because Prdm16 is a transcriptional regulator, we performed transcriptional profiling using RNAseq to compare heart tissue of male and female *Prdm16^csp1/wt^* mice with corresponding controls. Comparing female to male control heart tissue, we found 51 up-regulated and 92 down-regulated genes, applying an absolute log2 fold change (LFC) threshold > 0.5 (*Figure 3A*). Male *Prdm16^csp1/wt^* mice showed 12 up-regulated and 16 down-regulated genes compared to male controls. Female *Prdm16^csp1/wt^* mice revealed 55 up-regulated and 35 down-regulated genes compared to controls. Transcriptional profiling showed that *Prdm16* underwent the most significant up-regulation in males and females, suggesting an autoregulatory control of expression (*Figure 3B* and *C*). Other transcripts of the PRDM gene family did not exhibit changes in expression (see Supplementary material online, *Figure SVA* and *B* in the Data Supplement).

Next, we examined the top 20 up- and down-regulated genes for males and females. In male *Prdm16^csp1/wt^* mice, we observed up-regulation for MBL associated serine protease 2 (*Masp2*), synapsin II (*Syn2*) a coat protein of clathrin-coated vesicles, and UbiA prenyltransferase domain-containing 1 (*Ubiad1*) controlling coenzyme Q10 synthesis (*Figure 3D*). We detected decreased levels of transcripts for synuclein alpha (*Snca*), haemoglobin alpha adult chain 1 (*Hba-a1*), and haemoglobin subunit beta (*Hbb-ba*). Hba-a1 and Hbb-ba supply/control intracellular oxygen and become differentially regulated upon beta-adrenergic activation in a CaM kinase II dependent manner.^19^ Female *Prdm16^csp1/wt^* cardiac tissue showed up-regulation of the extra cellular matrix CUB domain-containing protein 1 (*Cdcp1*), ladinin (*Lad1*) involved in basement membrane organization, the cell adhesion protein spondin 2 (*Spon2*), nuclear receptor subfamily 1, group D, member 1 (*Nr1d1*), a transcriptional repressor co-ordinating metabolic pathways, and nicotinamide riboside kinase 2 (*Nmrk2*) regulating laminin matrix deposition. Aldehyde dehydrogenase family 3, subfamily A1 (*Aldh3a1*) and gastrokine 3 (*Gkn3*) showed strongest down-regulation in female *Prdm16^csp1/wt^* cardiac tissue. The top 20 genes were tested for their cellular expression in heart tissue (www.proteinatlas.org). Most genes showed a broad expression in cardiomyocytes, endothelial cells, fibroblasts, immune cells, and smooth muscle cells. Thus, no enrichment of cell lineage-specific genes was found. The majority of dysregulated genes in male and female *Prdm16^csp1/wt^* cardiac tissue is associated with transcription (T), metabolism (M), and indirect/direct immune response (I). Next, we evaluated the top 20 up- and down-regulated transcripts from male and female *Prdm16^csp1/wt^* hearts with sex-specific transcription pattern in rat cardiomyocytes.^20^ This analysis revealed that *Pbxip1* shows significant higher levels in females. Moreover, the transcripts of activity regulated cytoskeletal-associated protein (*Arc*), Jun B proto-oncogene (*Junb*), *Nr1d1*, period circadian clock 1 (*Per1*) appeared more abundant in female cardiomyocytes, while the transcripts centromere protein F (*Cenpf*), antigen identified by monoclonal antibody Ki 67 (*Mki67*), collagen 1 a1 (*Col1a1*), and collagen 3 a1 (*Col3a1*) were more abundant in male cardiomyocytes.

Concordant and discordant changes in males vs. females were assessed with LFC significance threshold of >0.25 and <−0.25, respectively. The strongest concordant up-regulated genes in *Prdm16^csp1/wt^* hearts were *Prdm16*, *Lad1*, *Ubiad1*, and the pre-B-cell leukaemia transcription factor interacting protein 1 (*Pbxip1*) (*Figure 3E*). We examined whether there was a systematic dysregulation of critical cardiac genes using the harmonizome gene set *congenital heart disease*. This did not indicate a consistent, significant dysregulation of cardiac-specific transcripts (see Supplementary material online, *Figure SVC* in the Data Supplement). Differentially expressed genes from *Prdm16^csp1/wt^* hearts (TOP5 up- and down-regulated genes) and known PRDM16 interacting genes (physical interaction, transcriptional targets, transcriptional complex component) were tested for dysregulation in a dataset obtained from human DCM patients.^21^ From over 60 known PRDM16 targets, we found that *PPARA*, *MED1*, and *CEBPD* were dysregulated in the human DCM screen (see Supplementary material online, *Table SVII* in the Data Supplement). Moreover, the chemotactic factor 2 (*Ccl2*) was dysregulated in DCM patients and in male *Prdm16^csp1/wt^* mice. Sex-specific gene expression validated correct technical procedures of RNAseq *Prdm16^csp1/wt^* mice analysis (see Supplementary material online, *Figure SVD* in the Data Supplement). Next, we generated a gene ontology (GO) network using the male and female TOP20 up- and down-regulated genes in *Prdm16^csp1/wt^* hearts (GOnet).^18^ We found the strongest functional association of dysregulated genes in *Prdm16^csp1/wt^* hearts for the GO terms *response to lipids* and *response to oxygen-containing compounds* (*Figure 3F*, Supplementary material online, *Figure SVE* in the Data Supplement). We verified up-regulated expression of *Pbxip1* and *Pyroxd2* in *Prdm16^csp1/wt^* hearts using qPCR (*Figure 3G*). Normal *Pbxip1* and *Pyroxd2* transcript levels in skeletal muscle suggests a cardiac-specific transcriptional response. Down-regulation of *Hba-a1* and *Hbb-ba* was also confirmed in male *Prdm16^csp1/wt^* hearts by qPCR (*Figure 3H*). Thus, *Prdm16^csp1/wt^* hearts show moderate, sex-specific transcriptional dysregulation, with *Prdm16*, *Lad1*, *Ubiad1*, and *Pbxip1* representing the most strongly regulated transcripts. GO and individual transcript evaluation suggest an impact on metabolic processes after monoallelic *Prdm16* inactivation.

### 3.3 Proteome analysis of *Prdm16^csp1/wt^* hearts reveals an up-regulation of *Pbxip1* and *Pyroxd2*

**Figure 4:**
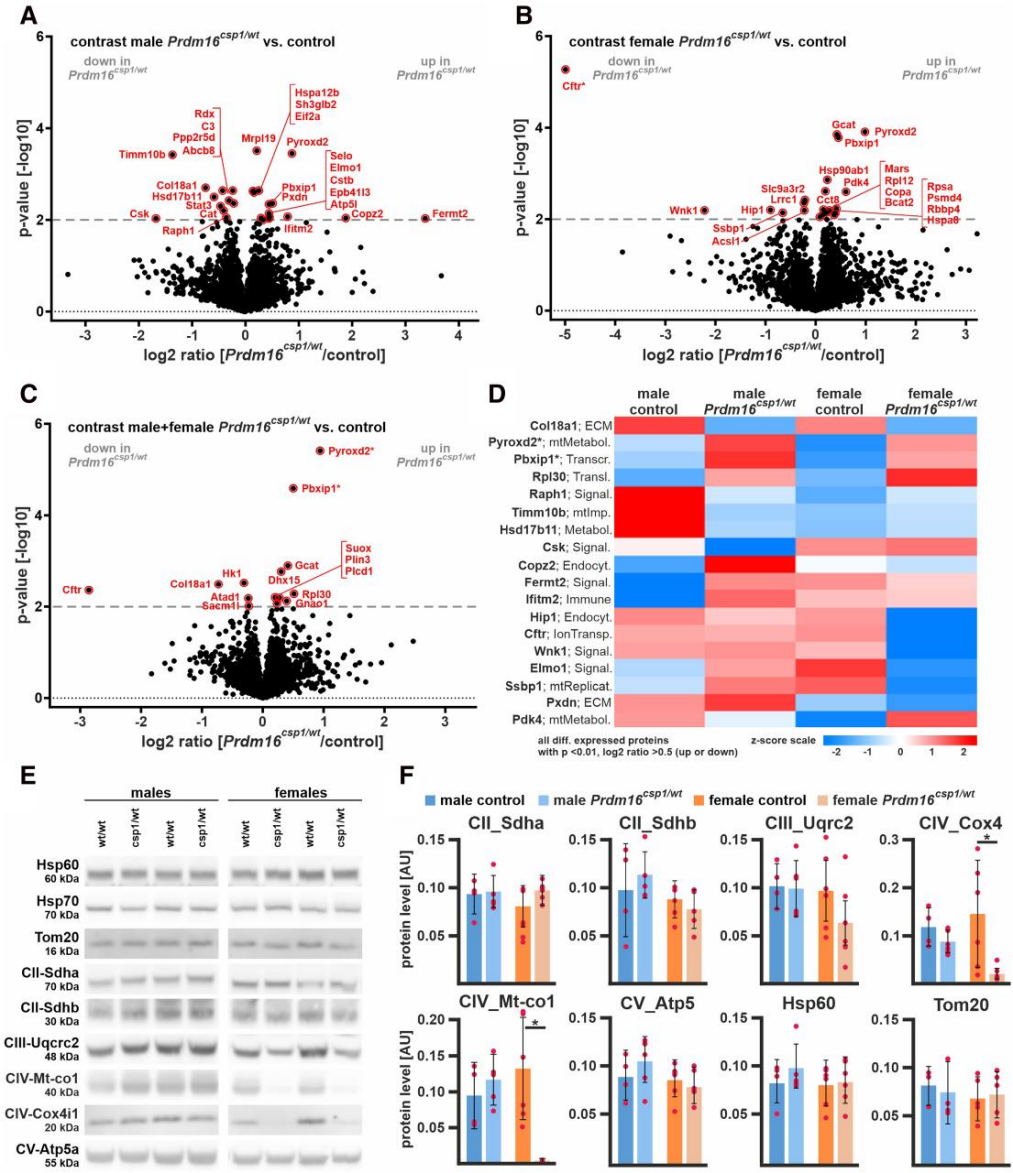
Proteome comparison of *Prdm16^csp1/wt^* cardiac tissue. Volcano plots for *Prdm16^csp1/wt^*/control pairwise comparisons with log2 ratio (*x*-axis) and *P*-value (*y*-axis) of male hearts (*A*), female hearts (*B*), and combined analysis of male + female hearts (*C*). Colouring indicates significantly altered proteins (threshold dashed grey line, *P*-value < 0.01), and * highlights highly significantly different proteins with *P*-value < 0.0001. (*D*) Heat map of all regulated proteins (*P*-value < 0.01, abs log2 ratio > 0.5) using *z*-scored values of group median intensity. Pre-B-cell leukaemia transcription factor interacting protein 1 (Pbxip1) and pyridine nucleotide-disulphide oxidoreductase domain 2 (Pyroxd2) are consistently increased in male and female *Prdm16^csp1/wt^* mice with *P*-value < 0.0001. Analysed biological replicates are *n* = 4 in each group. (*E*) Western blot analysis shows protein expression of selected mitochondrial proteins. (*F*) Quantification of western blot data showing mitochondrial protein levels. Analysed biological replicates are *n* > 4. Statistical analysis was performed with unpaired *t*-test, * indicates *P* < 0.05.

To validate our findings from the transcriptional analysis and to assess cardiac tissue protein expression, we performed a global proteomic analysis of cardiac tissue. Consistent with our findings from RNAseq, male *Prdm16^csp1/wt^* cardiac tissue exhibited fewer differences from controls than female samples. Of the 3847 proteins identified in total, 3314 were used for quantitation. Sample identity was confirmed by levels of expression of the male-specific DEAD box helicase 3, Y-linked (Ddx3y) and eukaryotic translation initiation factor 2, subunit 3, structural gene Y-linked (Eif2s3y). The Prdm16 protein was not detected, which can be explained by very low abundance, below the limit of detection. The most significant differences in protein expression were observed between male and female cardiac tissues (165 proteins with FDR < 5%, data not shown). A pairwise evaluation of control and *Prdm16^csp1/wt^* proteome data from male cardiac tissue using a *P*-value cut-off 0.01 showed the up-regulation of fermitin family member 2 (Fermt2), coatomer protein complex, subunit zeta 2 (Copz2), and pyridine nucleotide-disulphide oxidoreductase domain 2 (Pyroxd2) (*Figure 4A*). Substantial down-regulation was observed for c-src tyrosine kinase (Csk), translocase of inner mitochondrial membrane 10B (Timm10b), and collagen type XVIII, alpha 1 (Col18a1) proteins. A pairwise evaluation of proteome data from female cardiac tissue showed the strongest up-regulation for Pyroxd2, pyruvate dehydrogenase kinase, isoenzyme 4 (Pdk4), and pre-B-cell leukaemia transcription factor interacting protein 1 (Pbxip1) in *Prdm16^csp1/wt^* mice (*Figure 4B*). The strongest down-regulation was detected for cystic fibrosis transmembrane conductance regulator (Cftr), WNK lysine deficient protein kinase 1 (Wnk1), and huntingtin interacting protein 1 (Hip1).

To further assess *Prdm16* genotype driven differences, we performed also a pairwise evaluation of combined male and female groups (*P*-value cut-off 0.01). In total, we identified 53 up- or down-regulated proteins (*Figure 4C*, Supplementary material online, *Figure SVI* in the Data Supplement). Pyroxd2, Pbxip1, and ribosomal protein L30 (Rpl30) appeared as the strongest concordantly up-regulated proteins in *Prdm16^csp1/wt^* cardiac tissue. The strongest concordant down-regulation was found for Cftr, Col18a1, and hexokinase 1 (Hk1) in *Prdm16^csp1/wt^* cardiac tissue. By adding an additional filter (abs log2 ratio > 0.5), the top regulated proteins (*n* = 18) were selected (*Figure 4D*). Among these, Pyroxd2 and Pbxip1 represent the two most significantly regulated candidates in *Prdm16^csp1/wt^* cardiac tissue (*P*-value < 0.0001). Pyroxd2 is a critical regulator of hepatic mitochondrial function. Pyroxd2 interacts with mitochondrial complex IV (CIV) and appears consistently up-regulated in *Prdm16^csp1/wt^* hearts on both transcript and protein levels.^22^ Pbxip1 interacts with transport as well as regulatory proteins and indirectly affects transcription.^23^ Global proteome data did not show differential expression of mitochondrial transport proteins and respiratory chain complexes (see Supplementary material online, *Figure SVII* in the Data Supplement). Expression analysis of mitochondrial markers reveals normal expression of heat shock protein 60 (Hsp60), mitochondrial import receptor subunit Tom20 homologue (Tom20), or succinate dehydrogenase a (Sdha) (*Figure 4E* and *F*). Of note, protein levels of cytochrome c oxidase subunit 1 (Mt-Co1) and cytochrome c oxidase subunit 4 isoform 1 (Cox4i1), two components of the mitochondrial CIV, appear significantly diminished. Proteome data for sarcomere components, glycolysis, and amino acid metabolism did not show differential expression (see Supplementary material online, *Figure SVIII* in the Data Supplement). Altogether, the protein expression analysis identifies the specific dysregulation of the mitochondrial CIV proteins Pyroxd2, Mt-Co1, Cox4i1, and the transcriptional regulator Pbxip1.

### 3.4 Altered metabolism in *Prdm16^csp1/wt^* cardiac tissue

**Figure 5:**
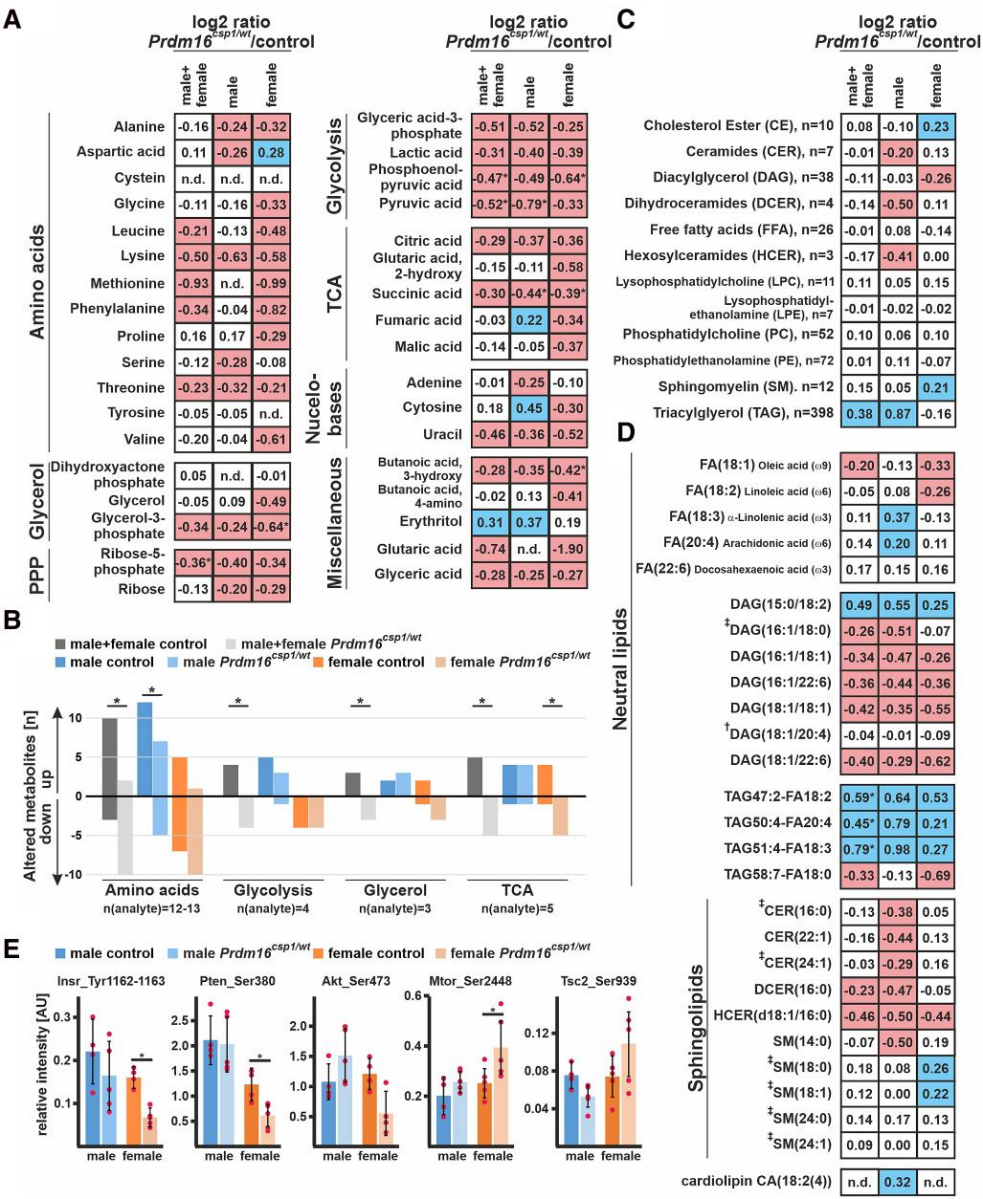
Altered metabolism in *Prdm16^csp1/wt^* cardiac tissue. (*A*) Normalized central carbon metabolite counts are presented as log2 value of the mean of normalized peak area ratio *Prdm16^csp1/wt^*/controls of males, females, and combination of both sexes. In female *Prdm16^csp1/wt^* LV tissue, broad suppression of several metabolic pathways was observed. In male *Prdm16^csp1/wt^* LV tissue, a similar but less pronounced reduction was detected. Statistical analysis of individual metabolites was performed with non-parametric Wilcoxon Rank Sum test, * indicates *P* < 0.05. (*B*) Univariate analysis reveals in cardiac tissue of *Prdm16^csp1/wt^* mice significant reduction of the amino acid, glycolysis, glycerol, and tricarboxylic acid cycle (TCA) metabolism using combined male and female data. Divergent metabolism of male and female animals was observed for amino acid metabolism, glycolysis, and TCA cycle. (*C*) Lipid analysis was performed with LC-MS using the lipidizer kit (Sciex). Global lipid analysis and evaluation as log2 value of the intensity ratio *Prdm16^csp1/wt^*/controls reveal normal levels for most lipid classes. Strongest regulation was observed for triacylglycerol (TAG) in male *Prdm16^csp1/wt^* hearts. The number (*n*) of validly detected lipids per class is indicated. (*D*) Selected neutral lipids and sphingolipids critical for the heart, lipids altered in *Prdm16^csp1/wt^* mice, and lipids previously associated with heart function (^†^Tham *et al.*,^24^  ^‡^Wittenbecher *et al.*^25^) are presented for *Prdm16^csp1/wt^* cardiac tissue of both sexes and in combination. Values for phospholipids are available in Supplementary material online, *Figure SIX* in the Data Supplement. Analysed biological replicates for all metabolic analysis are *n* = 4–6. (*E*) Signalling activity of the mitogen-activated protein kinase (MAPK) and mTOR pathway was assessed with the Milliplex phosphoprotein magnetic bead system and revealed diminished phosphorylation of insulin receptor (Insr_Tyr1162-1163), phosphatase and tensin homologue (Pten_Ser380), and Akt serine/threonine kinase 2 (Akt_Ser473) in female *Prdm16^csp1/wt^* mice. Analysed biological replicates for all metabolic analysis are *n* = 4–6. Statistical analysis of selected lipids was performed with non-parametric Wilcoxon Sum Rank test, * indicates *P* < 0.05. Colouring indicates reduction (red) or increase (blue) of the log2 of ratio by −0.2 or 0.2, respectively.

Because our expression analysis indicated that Prdm16 was likely affecting metabolism in cardiac tissue, we analysed central carbon metabolites in *Prdm16^csp1/wt^* cardiac tissue using gas chromatography-mass spectrometry (GC-MS). The measured values are presented as log2 from ratio of mean of the normalized peak areas *Prdm16^csp1/wt^*/controls (values > 0.2, blue and values < −0.2, red). Overall, we detected a diminishment of several metabolites (log2 ratios *Prdm16^csp1/wt^*/controls) for all of the metabolic processes we assessed (*Figure 5A*). We observed reductions of amino acid, glycerol, pentose phosphate pathway (PPP), glycolysis, tricarboxylic acid cycle (TCA), and nucleobase metabolism in *Prdm16^csp1/wt^* cardiac tissue. An analysis of individual metabolites in female *Prdm16^csp1/wt^* cardiac tissue revealed statistically significant reductions for glycerol-3-phosphate, phosphoenolpyruvic acid, succinic acid, and 3-hydroxy butanoic acid. Combining the analysis of female and male cardiac tissue, we identified significant reductions of phosphoenolpyruvic acid, pyruvic acid, and ribose-5-phosphate in *Prdm16^csp1/wt^* hearts. Because an interpretation of individual metabolites is difficult, we assessed the pathway profiles of central carbon metabolites through univariate analysis. This method counts each increased/diminished metabolite as an ordinary number. Combining the pathway analysis using female and male values identified a significant reduction of amino acid, glycolysis, glycerol, and TCA metabolism in *Prdm16^csp1/wt^* hearts (*Figure 5B*).

The adult heart mainly relies on FA oxidation as its primary substrate, so we next explored lipid metabolism with liquid chromatography-mass spectrometry (LC-MS) in depth. Values are presented as log2 from ratio *Prdm16^csp1/wt^*/controls (values > 0.2, blue and values < −0.2, red). Our evaluation of lipid classes did not reveal any significant dysregulation. The strongest changes were observed in male *Prdm16^csp1/wt^* hearts for triacylglycerol compounds (TAG; increase), dihydroceramides (DCER; reduction), and hexosylceramides (HCER; reduction) (*Figure 5C*). Currently, little is known about the dysregulation of individual lipid classes in early cardiac dysfunction.^24,25^ Individual lipids are presented as log2 of ratio *Prdm16^csp1/wt^*/controls. Evaluation of accumulated lipid classes appears widely normal. However, a few individual lipids were dysregulated (see Supplementary material online, *Figure SIXA* in the Data Supplement). Important FAs such as oleic, linoleic, α-linolenic, and arachidonic acids show moderate changes (*Figure 5D*). Several diacylglycerols (DAG) appear strongly reduced in *Prdm16^csp1/wt^* hearts: DAG(16:1/18:0), for instance, was found to be altered in a study assessing lipid profiles in human heart failure.^25^ In contrast, DAG(18:1/20:4) was significantly altered in lipidomic profiling of murine hearts after exercise or pressure overload, but was normal in *Prdm16^csp1/wt^* hearts.^24^ There was a significant enrichment of TAG47:2-FA18:2, TAG50:4-FA20:4, and TAG51:4-FA18:3 in males and somewhat lower levels in female *Prdm16^csp1/wt^* cardiac tissue.

The sphingolipids sphingomyelin SM(14:0), dihydroceramide DCER(16:0), and ceramide CER(22:1) exhibited the strongest reduction in males. Lower levels of the hexosylceramide HCER(d18:1/16:0) were found in the hearts of both *Prdm16^csp1/wt^* sexes. Several protective and risk predictive sphingolipids identified by Wittenbecher *et al.*^25^ were unaffected in *Prdm16^csp1/wt^* mice. Global levels of the cardiac phospholipids phosphatidylcholine (PC) and phosphatidylethanolamine (PE) were unaffected (*Figure 5C*). Individual PC and PE species were consistently higher [e.g. PC(18:2/18:3), PE(17:0/22:5), and PE(P-18:0/18:0)] or lower [e.g. PC(18:1/16:1), PE(P-18:1/18:1), and PE(P-18:1/18:2)] in *Prdm16^csp1/wt^* hearts of both sexes (see Supplementary material online, *Figure SIXB* in the Data Supplement). The level of cardiolipin [CA(18:2(4)], a phospholipid critical for mitochondrial function, appeared moderately elevated in male *Prdm16^csp1/wt^* hearts (*Figure 5D*). In summary, global lipid metabolism is altered in male *Prdm16^csp1/wt^* hearts, with an accumulation of TAG.

Cardiac metabolism is controlled by the mitogen-activated protein kinase (MAPK) and mechanistic target of Rapamycin (mTOR) pathways. Using the Milliplex phosphoprotein magnetic bead system, we tested the phosphorylation level of key proteins from these pathways. Female *Prdm16^csp1/wt^* hearts exhibited a significant phosphorylation inactivation for insulin receptor (Insr_Tyr1162-1163), phosphatase and tensin homologue (Pten_Ser380), and Akt serine/threonine kinase 2 (Akt_Ser473) (*Figure 5E*). We also found an increase of phosphorylation activation of mammalian Target of Rapamycin (Mtor_Ser2448) and tuberin (Tsc2_Ser939). Thus, diminished phosphorylation of the Insr/Akt pathway activates mTOR signalling and cardiac metabolism adapts accordingly. Altogether, *Prdm16^csp1/wt^* hearts show multiple, substantial metabolic changes suggesting that the inactivation of *Prdm16* affects cardiac metabolism.

### 3.5 Tissue energetics and production of protective lipids in *Prdm16^csp1/wt^* hearts

**Figure 6:**
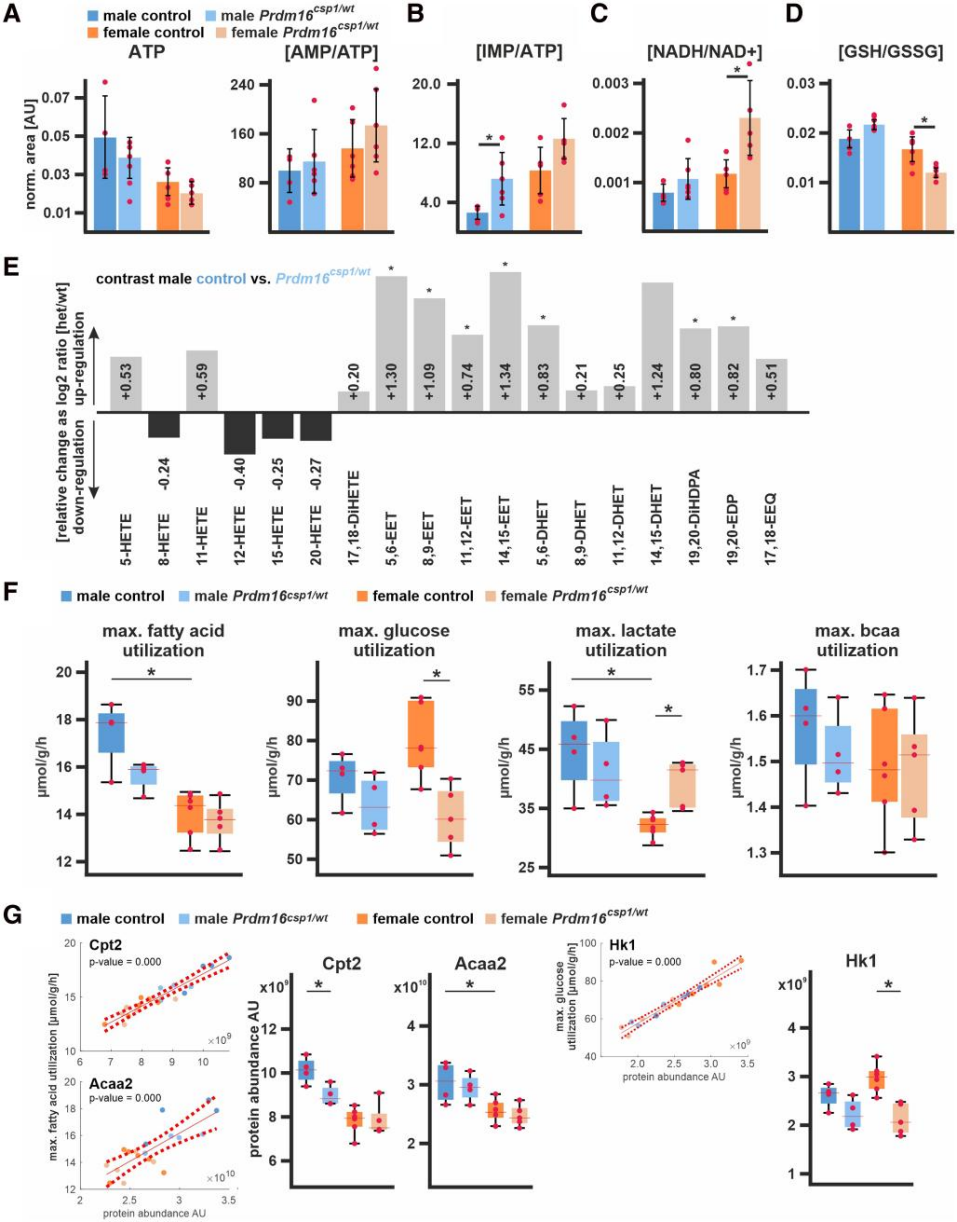
Nutrient metabolism in *Prdm16^csp1/wt^* cardiac tissue. (*A*) Assessment of metabolites critical for energy metabolism using LV tissue and LC-MS. Normalized values are shown for adenosine triphosphate (ATP) and the ratio of adenosine monophosphate (AMP) vs. ATP (AMP/ATP). Analysed biological replicates are *n* = 4–6 for *A* to *D*. (*B*) The ratio of inosine monophosphate (IMP) vs. ATP (IMP/ATP) is increased in *Prdm16^csp1/wt^* hearts. (*C*) The ratio of reduced vs. oxidized nicotinamide adenine dinucleotide (NADH/NAD+) is increased in female *Prdm16^csp1/wt^* hearts. (*D*) Oxidative capacity of cardiac tissues was assessed with the ratio of reduced vs. oxidized glutathione (GSH/GSSG). Female *Prdm16^csp1/wt^* hearts show a significantly reduced GSH/GSSG ratio. (*E*) Eicosanoids were measured in male *Prdm16^csp1/wt^* hearts with LC/ESI-MS-MS. Data are presented as log2 *Prdm16^csp1/wt^*/controls ratio with down- or up-regulation as black or grey bars, respectively. All epoxyeicosatrienoic (EET) and dihydroxyeicosatrienoic (DHET) acids are increased in *Prdm16^csp1/wt^* cardiac tissue. Corresponding absolute measurements are available in Supplementary material online, *Table SVIII* in the Data Supplement. Analysed biological replicates are *n* > 6. (*F*) Modelling of major cardiac metabolic processes occurred with CARDIOKIN1^26^ using protein expression data. Differences in maximal substrate utilization for fatty acids (FA), glucose, lactate, and branched chain amino acids (bcaa). Box plots show median and 25% quartile. Dots depict maximal capacities for individual animals. (*G*) Individual protein impact for FA and glucose metabolism was correlated for carnitine palmitoyltransferase 2 (Cpt2), acetyl-CoA acyltransferase 2 (Acaa2), and hexokinase 1 (Hk1) using linear regression analysis of the maximal substrate utilization vs. protein abundance (dashed lines indicate confidence interval, 95%). Box plots show median and 25% quartile. Dots depict Cpt2, Acaa2, and Hk1 maximal capacities for individual animals. Statistical analysis of individual metabolites and processes was performed with unpaired *t*-test, * indicates *P* < 0.05. Analysed biological replicates are *n* = 4–6.

To further explore the consequences of imbalanced cardiac metabolism in *Prdm16^csp1/wt^* mice, we tested the steady-state levels of important cardiac metabolic intermediates, redox molecules, and eicosanoids. Global adenosine triphosphate (ATP) levels were unaffected in *Prdm16^csp1/wt^* cardiac tissue (*Figure 6A*, Supplementary material online, *Table SVIII* in the Data Supplement). However, the ratio of adenosine monophosphate to ATP (AMP/ATP) was increased in female *Prdm16^csp1/wt^* hearts. AMP is a molecule that is critical for sensing metabolic stress conditions, and the increased AMP/ATP ratio suggests an abnormal energy state in *Prdm16^csp1/wt^* cardiac tissue. We also detected increases in the levels of absolute and relative inosine monophosphate (IMP) in both male and female *Prdm16^csp1/wt^* hearts (*Figure 6B*). IMP is the key molecule in purine metabolism and a sensitive measure of ATP turnover.^27^ The level of hypoxanthine, which serves as precursor and degradation product of IMP, was elevated in male *Prdm16^csp1/wt^* hearts. The ratio of reduced to oxidized nicotinamide adenine dinucleotide (NADH/NAD+), which serves as a central hydride donor for oxidative phosphorylation and many other redox reactions,^28^ was increased in female *Prdm16^csp1/wt^* hearts (*Figure 6C*). The ratio of reduced to oxidized glutathione (GSH/GSSG) was diminished in female *Prdm16^csp1/wt^* hearts, suggesting accelerated cellular GSH consumption and redox imbalance (*Figure 6D*). Levels of creatine, phosphor-creatine/creatine, and acetyl-CoA appeared normal. The level of 4-hydroxynonenal (4-HNE), a marker of lipid peroxidation, was unaffected. These findings suggest accelerated ATP turnover and oxidative stress but no lipid peroxidation after *Prdm16* inactivation, particularly in female *Prdm16^csp1/wt^* hearts.

Aside from their functions as a source of nutrition, FAs serve as reactants for biosynthesis of hydroxyeicosatetraenoic acids (HETE) and epoxyeicosatrienoic acids (EET).^29,30^ All HETE species, which are synthesized from arachidonic acid by lipoxygenases (LOX), were normal in male *Prdm16^csp1/wt^* cardiac tissue (*Figure 6E*, Supplementary material online, *Table SIX* in the Data Supplement). 20-HETE, which is synthesized from arachidonic acid by cytochrome P450 (CYP), was also unaffected. All the individual EETs we tested, which originate from CYP activity, and the total amount of EETs were significantly increased. Consistently, the water soluble dihydroxyeicosatrienoic acids (DHET), which represent the corresponding EET hydrolyzation products, also exhibited significant increase. We also found higher levels of the anti-inflammatory 19,20-epoxydocosapentaenoic acid (19,20-EDP) and 17,18-epoxyeicosatetraenoic acid (17,18-EEQ). These observations suggest an activation of CYP-mediated bioactive, lipid production (EETs and 19,20-EDP) in male *Prdm16^csp1/wt^* cardiac tissue.

To globally assess metabolism in *Prdm16^csp1/wt^* cardiac tissue, we modelled major metabolic pathways using CARDIOKIN1 *in silico*. As input, we used quantitative data regarding the expression of metabolically important proteins as measured with LC-MS/MS (*Figure 4*). The maximal utilization rates calculated for of the given metabolic condition are upper estimates that will probably not be reached under physiological conditions. However, these maximal utilization rates allow for an estimation of the capacity under such conditions. The maximum rates for the utilization of FA and glucose were different in cardiac tissue of male and female control hearts. Females had a lower capacity for FA utilization but higher for glucose compared to male controls (*Figure 6F*). In addition, the maximum rate for the utilization of lactate was lower in female controls. In male *Prdm16^csp1/wt^* cardiac tissue, the maximum use of FA decreased but was unaffected in females. In contrast, maximum use of glucose was diminished in female *Prdm16^csp1/wt^* cardiac tissue, while it was unaffected in the males. The maximum utilization of lactate increased in female *Prdm16^csp1/wt^* cardiac tissue.

We also modelled the maximum ATP production and O_2_ consumption under fasting and post-prandial conditions. In both conditions, the cardiac tissue of female controls showed lower maximum ATP production and O_2_ consumption compared to male controls (see Supplementary material online, *Figure SX* in the Data Supplement).

The maximum utilization rate gives a net measure for several members of the given metabolic pathway. Thus, we aimed to identify enzymes critical for FA and glucose metabolism in *Prdm16^csp1/wt^* cardiac tissue. For this purpose, the metabolic utilization and protein abundance were correlated for each protein and animal. The strongest correlations, indicated by low *P*-values, were found for carnitine palmitoyltransferase 2 (Cpt2), acetyl-CoA acyltransferase 2 (Acaa2), hexokinase 1 (Hk1), and other proteins (see *Figure 6G*, Supplementary material online, *Figure SXI* in the Data Supplement). In controls, Cpt2 and Acaa2, two proteins of the mitochondrial beta-oxidation activity, showed lower maximal capacity in females, supporting our finding that FA metabolism is lower (*Figure 6G*, Supplementary material online, *Figure SXII* in the Data Supplement). Lower Cpt2 capacity in male *Prdm16^csp1/wt^* cardiac tissue partly explains lower FA metabolism in males. Consistent with protein expression data, female and male *Prdm16^csp1/wt^* cardiac tissue showed a lower Hk1 utilization. Hk1 mediates phosphorylation of D-glucose to D-glucose 6-phosphate and represents the initial step of glycolysis. Altogether, these findings indicate an imbalance in substrate metabolism and oxidative stress upon a monoallelic inactivation of *Prdm16*.

## 4. Discussion

**Figure 7:**
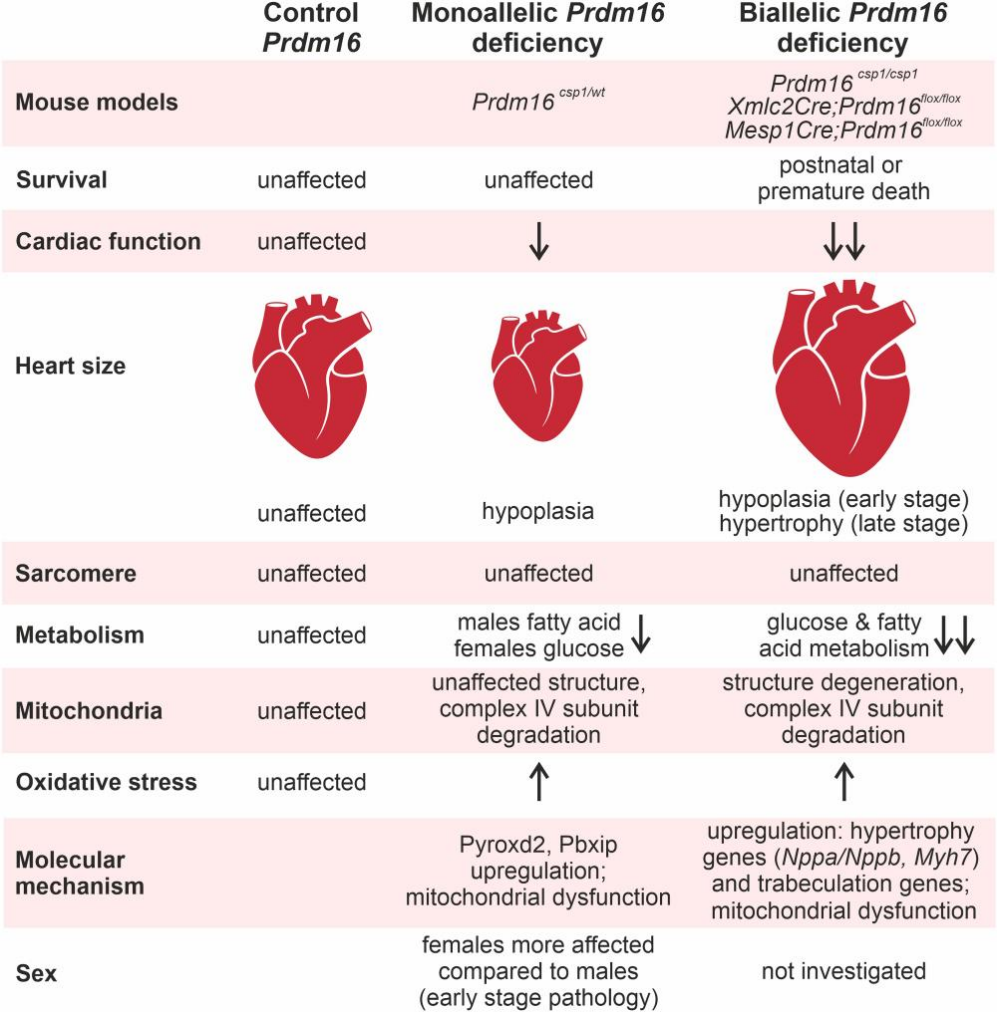
Prdm16 is an early regulator of cardiac metabolism. Assessing different mouse models for the *PRDM16* associated cardiomyopathy reveals distinct cardiac phenotypes after mono or biallelic Prdm16 inactivation.^9,10,31^ Metabolism is differentially affected at early (*Prdm16^csp1/wt^*) and late pathology stages (*Xmlc2Cre;Prdm16^flox/flox^*, *Mesp1Cre;Prdm16^flox/flox^*).^10,31^

Our study suggests that a decisive event in the *Prdm16* associated early cardiac pathology involves an extensive dysregulation of metabolism, without extensive effects on transcription (*Figure 7*). Metabolic changes upon heterozygous *Prdm16* inactivation appear to contribute to the development of early cardiac pathologies, which may result in cardiac hypoplasia^9^ and hypertrophy.^31^ We identify Pyroxd2 and Pbxip1 as novel modulators of cardiac function, which reflect the central role of metabolism in the *Prdm16* associated cardiac phenotype. Moreover, our study reveals that *Prdm16^csp1/wt^* females experience a more pronounced molecular and structural phenotype than males, and that sex has a larger effect than the *Prdm16* genotype. The difference between male and female *Prdm16^csp1/wt^* hearts in the maximum use of FA and glucose suggests that females are more poorly equipped to cope with metabolic challenges.

### 4.1 *PRDM16* cardiomyopathy

Mutation of *PRDM16* is known to cause cardiomyopathy associated with DCM and LVNC.^4^ More recently, truncating variants in *PRDM16* have been identified by *in silico* analysis as one of three specific LVNC variant classes.^8^ These genetic observations and the *Prdm16^csp1/wt^* phenotype reinforce the case that heterozygous *PRDM16* inactivation/truncation in humans is sufficient to cause cardiomyopathy. Functional evidence from *Prdm16^csp1/wt^* mice, which mirrors the genotype of patients with *PRDM16* mutation, will increase the ClinGen evidence from *limited* to *moderate* for the association of *PRDM16* with cardiomyopathy (www.clinicalgenome.org).

In mice, a homozygous inactivation of *Prdm16* induces discordant phenotypes in different genetic backgrounds, resulting in either early post-natal lethality (*Xmlc2Cre;Prdm16^flox/flox^*) or cardiac dysfunction with fibrosis at adult stages (*Mesp1Cre;Prdm16^flox/flox^*, *Myh6Cre;Prdm16^flox/flox^*).^10,31,32^  *Xmlc2Cre* and *Mesp1Cre* strains are expected to inactivate *Prdm16* during the very early development of the heart.^33^ Discrepancies in the penetrance of the phenotypes can be attributed to incomplete spatial Cre-mediated *Prdm16* inactivation and residual Prdm16 levels in early cardiomyocyte progenitor cells, endothelia, or cardiac fibroblasts. Consistent with neuronal studies, it is likely that *Prdm16* has additional effects on cardiac stem and progenitor cell functions compared to differentiated, adult cardiomyocytes.^34^ Indeed, inactivating adult *Prdm16* using a tamoxifen-inducible mouse model (*αMHC-MerCreMer;Prdm16^flox/flox^*) resulted in viable mice without an overt cardiac phenotype.^10^ We conclude that the impact of Prdm16 on cardiomyocyte proliferation and growth depends on the developmental stage of the mice. So far, homozygous *PRDM16* mutations have not been identified in patients. Based on animal studies,^10,31,32^ we believe that biallelic *PRDM16* inactivation induces a highly penetrant, severe cardiac phenotype in humans that compromises post-natal life. In *Prdm16^csp1/wt^* hearts, we did not observe changes in myocardial compaction or the gene circuit involving Tbx5 and Hand1.^10^ This may be due to the heterozygous nature of the *Prdm16* inactivation in our model. Another important feature is that *Prdm16^csp1/wt^* hearts do not show perturbations of sarcomeres at either a structural or a molecular level. This suggests that contractile dysfunction in the *PRDM16* cardiomyopathy originates from other mechanisms such as energy restriction or metabolic stress, which affect contractility and/or ion homeostasis.

### 4.2 *Prdm16* differentially compromises metabolism in early and late-stage cardiac pathology

Studies from adipose tissue show that PRDM16 orchestrates adipocyte differentiation via its interaction with proteins such as PPARA, PPARG, MED1, CEBPD, peroxisome proliferator-activated receptor gamma coactivator 1-alpha, beta (PPARGC1A, PPARGC1B), or uncoupling protein 1 (UCP1).^3,12,13,15–17,35,36^ Consequently, we explored a selection of validated PRDM16 targets in *Prdm16^csp1/wt^* hearts and in a transcriptomic screen of human adult DCM (see Supplementary material online, *Table SVII* in the Data Supplement).^21^ We found a dysregulation of only three PRDM16 targets, namely PPARA, MED1, and CEBPD in this screen. We did not find changes in the regulation of Tbx5 or Hand1, which had been identified as targets in *Xmlc2Cre;Prdm16^flox/flox^* hearts.^10^ This suggests as follows: (i) the regulatory programs steered by PRDM16 in cardiomyocytes differ from those observed in other differentiated cell types, (ii) changes in expression are time sensitive and depend on the progenitor or differentiation stage, and (iii) the early molecular changes or adaptations in PRDM16 cardiac pathology are not associated with broad, significant expression changes. In the absence of major transcriptional effects in our model, we investigated possible metabolic alterations.

The initial evidence that metabolic alterations might be associated with the cardiac *PRDM16* phenotype came from a study analysing homozygous *Mesp1Cre;Prdm16^flox/flox^* mice. These mice develop cardiac hypertrophy, diminished heart function, fibrosis, a reduction in mitochondrial content, and diminished levels of acylcarnitine at 12 months of age.^31^ Diminished mitochondrial content is associated with the strong reduction of a mitochondrial CIV marker, which is in line with a degradation of Mt-co1 and Cox4i1 in *Prdm16^csp1/wt^* hearts. Furthermore, *Mesp1Cre;Prdm16^flox/flox^* hearts exhibit an up-regulation of transcripts involved in glucose metabolism, a transcriptional down-regulation of lipid metabolism, and changes in gene expression associated with oxidative stress. Challenging young *Mesp1Cre;Prdm16^flox/flox^* mice with a high-fat diet induced cardiac dysfunction and hypertrophy already at 3 months of age, compared to an onset at 9 months without the high-fat diet. This suggests that complete *Prdm16* inactivation in the heart leads to a diminished mitochondrial volume and sensitivity to substrate availability, ultimately resulting in cardiomyocyte hypertrophy by activating distinctive gene programs.^31^ An exploration of the heart phenotype in *Xmlc2Cre;Prdm16^flox/flox^* mice also revealed a down-regulation of transcripts associated with mitochondrial biogenesis/function and FA metabolism.^10^ Together, analyses of both models with homozygous *Prdm16* inactivation demonstrate that the cardiac phenotypes are associated with diminished metabolic capacity that eventually lead to cardiac growth and hypertrophy. In contrast, heterozygous *Prdm16^csp1/wt^* hearts are hypoplastic, with no evidence of mitochondrial volume changes. This indicates that metabolic alterations are early events in *Prdm16* inactivation.

Differences in male and female *Prdm16^csp1/wt^* responses with regard to maximal FA or glucose utilization support sex-specific transcriptional dysregulation. In male *Prdm16^csp1/wt^* hearts, the strongest up- and down-regulation was observed for *Ubiad1* and haemoglobins (*Hba-a1*, *Hbb-bs*), respectively. Ubiad1 is a prenyltransferase that is involved in ubiquinone (CoQ10) synthesis; its up-regulation increases redox tolerance and provides cardiovascular protection.^37^ The cellular down-regulation of Hba-a1 and Hbb-ba diminishes the oxygen supply and may blunt cardiac oxidative stress in a setting of restricted FA oxidation. Female *Prdm16^csp1/wt^* hearts show the strongest up- and down-regulation of the metabolism associated transcripts *Nr1d1* and *Aldh3a1*, respectively. NR1D1 is a ligand-regulated transcriptional repressor that affects metabolic regulation, cellular differentiation, and circadian rhythm control.^38^ Upon adipogenesis, *Nr1d1* gene silencing affects brown adipocyte differentiation and attenuates *Prdm16* expression, suggesting an interaction.^38^ A recent study exploring the impact of shift work on cardiac reperfusion injury revealed a drop in the myocardial Nr1d1 expression, suggesting that it might play a more general role in cardiomyocyte function.^39^ Aldh3a1 is involved in the oxidation and detoxification of lipid peroxids. Its genetic inactivation in zebrafish increases 4-HNE levels and impairs glucose homeostasis.^40^ Thus, Aldh3a1 reduction is likely associated with metabolic adaptations in the use of glucose in female *Prdm16^csp1/wt^*. Overall, early transcriptional changes in *Prdm16^csp1/wt^* hearts are distinct from those observed in later stages of pathology.

### 4.3 *Pyroxd2* and *Pbxip1* are novel modulators of cardiac function

In line with a central role of metabolism for PRDM16 associated cardiac phenotypes, we found a concordant up-regulation of Pyroxd2 and Pbxip1. Both have been implicated in the regulation of energy metabolism. PBXIP1 interacts with a number of proteins, including the transcription factor Pre-B-cell leukaemia factor 1 (PBX1),^41^ microtubules,^41^ oestrogen receptors 1 and 2 (ESR1, ESR2),^42^ and AMP-activated protein kinase (AMPK).^43^ Moreover, PBXIP1 regulates the activity of MAPK and mTOR signalling.^23^ The function of Pbxip1 in the heart is largely unknown. In an earlier genetic screen, the inhibition of MAPK signalling pathway proteins increased PBXIP1 phosphorylation.^44^ In contrast, the activation of the MAPK signalling cascade increased PBXIP1 protein expression. PBXIP1 overexpression in mice stimulated cardiac hypertrophy.^44^ In *Prdm16^csp1/wt^* hearts, we found that the up-regulation of Pbxip1 was associated with diminished MAPK signalling activity. Mutation of *PBXIP1* in patients has not been associated with heart disease; however, mutation of the interacting transcription factor PBX1 has been linked to syndromic congenital heart defects.^45^ An interesting aspect of PBXIP1 is its interaction with and activation of ESR1 and ESR2, which represent potential mechanisms for sex-specific effects.^42^ Our data implicate PBXIP1 in cardiac growth, metabolism, and hypertrophy, but this needs to be further characterized under conditions of health and disease. In the context of *Prdm16* heterozygous mutants, Pbxip1 may orchestrate the adaptive responses of important signalling cascades such as AMPK, mTOR, or MAPK.

The cardiac functions of murine Pyroxd2 are unknown. PYROXD2 is an oxidoreductase of the inner mitochondrial membrane/matrix that interacts with the mitochondrial CIV subunit Cox5b.^22^ The mitochondrial subunit Cox4i1, which is degraded in female *Prdm16^csp1/wt^* hearts, interacts with both Cox5b and Mt-co1. This suggests that CIV is critically affected upon *Prdm16* deactivation.^22,46^ A genetic inactivation of PYROXD2 in hepatic cell lines decreased the mitochondrial membrane potential, CIV activity, ATP content, and mitochondrial DNA copy number.^22^ PYROXD2 inactivation also increased amounts of mitochondrial reactive oxygen species and the number of immature mitochondria.^22^ Compound heterozygous genetic variants in *PYROXD2* were detected in a single patient with a severe infantile metabolic disorder.^47^ Molecular workups in patient fibroblasts demonstrated a mito-ribosomal defect characterized by increased mitochondrial superoxide levels, an elevated sensitivity to metabolic stress, a decrease in complex I subunit proteins, and diminished mitochondrial ribosome levels.^47^ Mutation of a related human oxidoreductase, *PYROXD1*, results in early-onset skeletal myopathy.^48^ Another study, which investigated molecular signatures in skeletal muscle from heart failure patients, detected an up-regulation of *PYROXD2* transcripts.^49^ These data suggest that the up-regulation of Pyroxd2 in *Prdm16^csp1/wt^* hearts may compensate for metabolic and oxidative stress conditions.

### 4.4 Sex-specific aspects of *Prdm16* inactivation in cardiac metabolism

Our study detects a more pronounced cardiac and molecular phenotype in *Prdm16^csp1/wt^* females than in males. Originally, the cytogenetic association of *PRDM16* inactivation with cardiomyopathy was described in 18 patients with 1p36 syndrome.^4^ Among these, 16 individuals were females and only two were males.^4^ This may point to a sex-specific penetrance of the *PRDM16* cardiomyopathy. Apparently, female *Prdm16^csp1/wt^* mice compensate for disturbances in cardiac metabolic less efficiently than males. Metabolic modelling with CARDIOKIN1 revealed diminished FA, increases in glucose, and reduced lactate utilization in the hearts of female than male *Prdm16^wt/wt^* mice.^26^ This establishes a clear sexual dimorphism for cardiac metabolism under basal healthy conditions.^50^ Under normal conditions, the heart generates ATP mainly via FA oxidation and glucose utilization (glycolysis, pyruvate supply to TCA).^51^ Female *Prdm16^csp1/wt^* hearts show a lower use of glucose but unaffected FA utilization. The increase in the maximal lactate utilization capacity in female *Prdm16^csp1/wt^* hearts likely reflects an adaptation to metabolic stress conditions. Male *Prdm16^csp1/wt^* hearts show diminished maximal utilization of FA, which is in line with the TAG accumulation we observed. Male *Prdm16^csp1/wt^* hearts exhibit elevated levels of EET, which is in line with a recent study demonstrating an increase in EET levels/turnover in LV biopsies from DCM patients.^52^ This supports distinct mechanisms of lipid metabolism in male hearts.

Whether hormonal differences are the cause of systemically reduced fat content and the more advanced pathology in female *Prdm16^csp1/wt^* mice is unclear. The known association of Pbxip1 and Esr1/Esr2 activity as well as Nr1d1 provides a potential explanation for the sexual dimorphism in the regulation of the use of substrates. *Prdm16^csp1/wt^* hearts demonstrate significant sex-related effects at both the transcript and protein levels. Indeed, sex has a larger effect on metabolic parameters than the *Prdm16* genotype, at least during the early pathological window. The fact that Pbxip1 and other genes are differentially regulated in sex-specific transcription patterns in *Prdm16^csp1/wt^* hearts partially explains the sex-specific phenotype that we observed.^20^ The recent identification of Prdm14 as a determinant of sex-biased expression patterns in early heart development further highlights the critical impact of the *Prdm* gene family as determinants of cardiac function.^53^ Our findings suggest a sexual dimorphism for *PRDM16* associated cardiomyopathy, with a more penetrant phenotype that likely occurs earlier in females.

### 4.5 Study limitations

Female *Prdm16^csp1/wt^* mice have diminished relative total body fat content and increased relative muscle content. This mirrors either the female tissue-specific metabolism and/or points towards a systemic factor affecting tissue development. Identification of such factor would be important to understand the systemic (endocrine) role of Prdm16 and to test therapeutic approaches targeting adipose tissue. Our study does not assess early metabolic changes in cardiac progenitor cells or individual cell types within the myocardium. Analysis of *Prdm16^csp1/wt^* hearts with *in silico* tools and individual metabolite measurements suggests an accelerated metabolism. However, we did not perform kinetic experiments to demonstrate accelerated turnover of selected metabolic pathways.

## Supplementary material

Supplementary material is available at *Cardiovascular Research* online.

## Supplementary Material

cvad154_Supplementary_Data

## Data Availability

The data underlying this article are available in the article, in its online supplementary material, and data repositories (GEO accession No. GSE236791, PRIDE accession No. PXD043601).
