# Supplementary material for: Prdm16 mutation determines sex-specific cardiac metabolism and identifies two novel cardiac metabolic regulators
Source: Cardiovasc Res. 2023 Oct 16;119(18):2902–16. doi: 10.1093/cvr/cvad154 (PMC10874277; doi:10.1093/cvr/cvad154)
Supplement: cvad154_Supplementary_Data [file cvad154_supplementary_data.zip › 190820_Figures_Western-blot.pdf]

Supplement to Figure 4E-F, page 1
Protein analysis Prdm16csp1/wt 8M

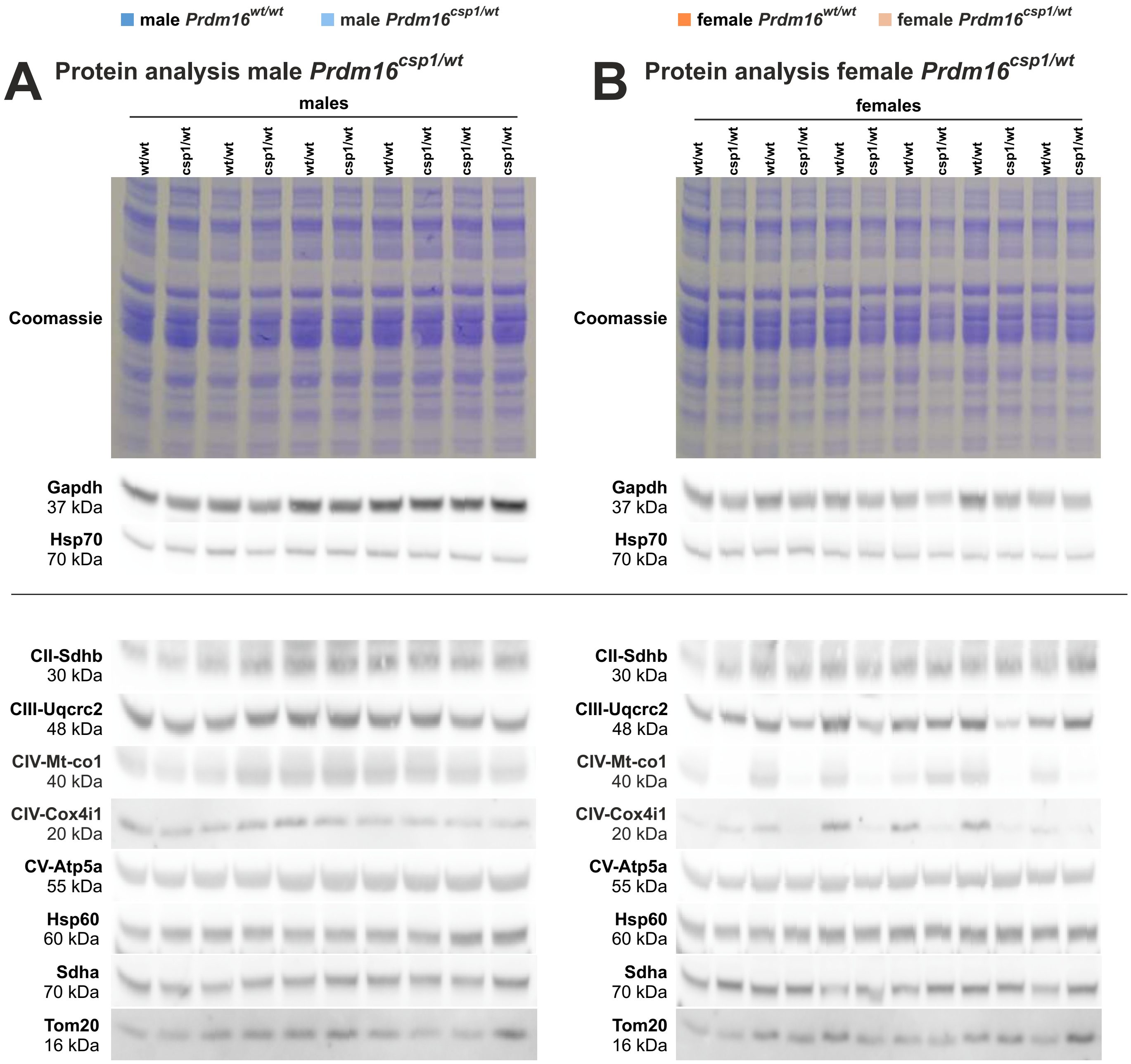

Supplement to Figure 4E-F, page 2
Original Western blots Prdm16csp1/wt, males, 8 months

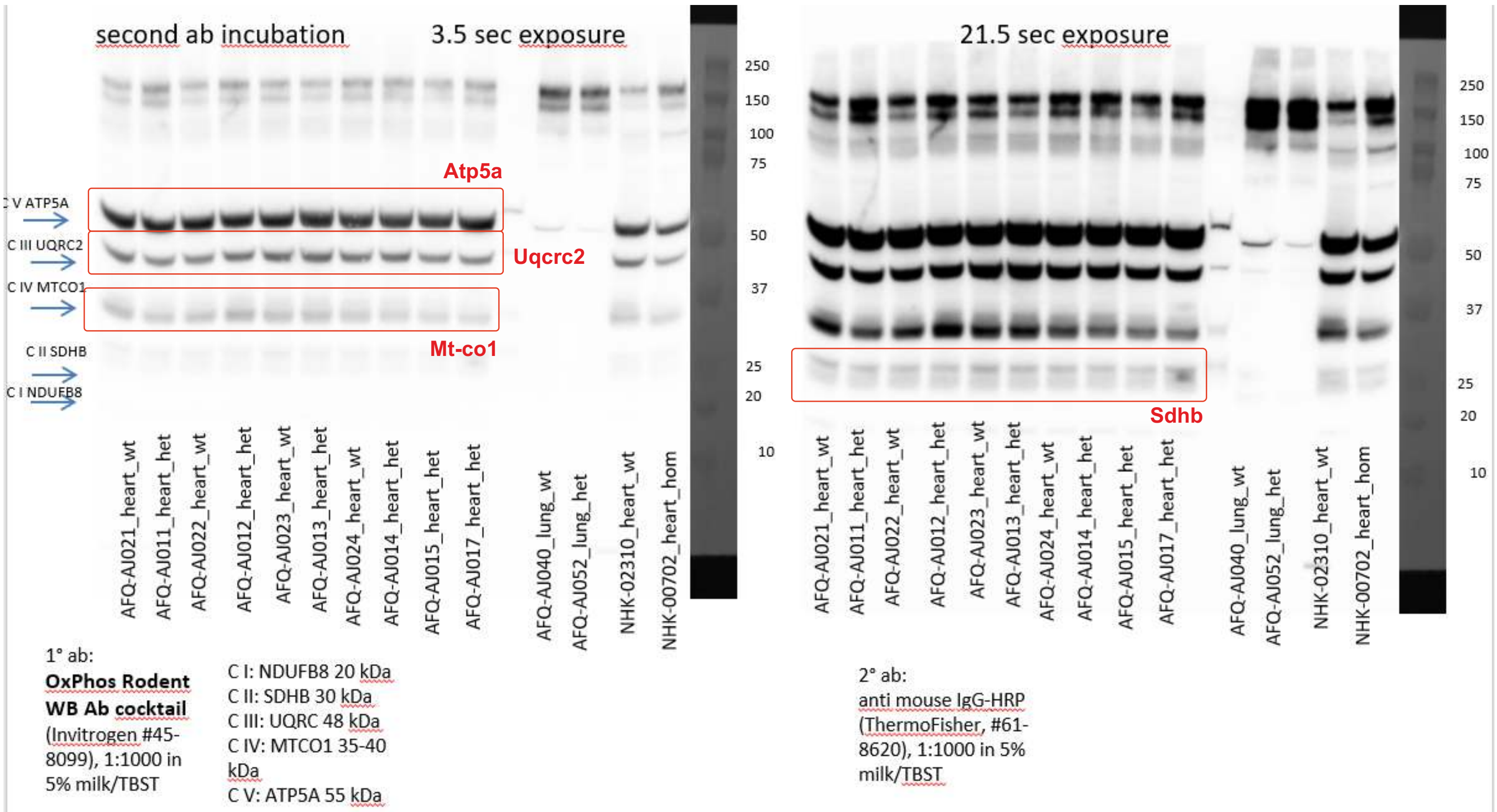

1° ab:
OxPhos Rodent WB Ab cocktail (Invitrogen #45-8099), 1:1000 in 5% milk/TBST

C I: NDUFB8 20 kDa
C II: SDHB 30 kDa
C III: UQRC 48 kDa
C IV: MTCO1 35-40 kDa
C V: ATP5A 55 kDa

2° ab:
anti mouse IgG-HRP (ThermoFisher, #61-8620), 1:1000 in 5% milk/TBST

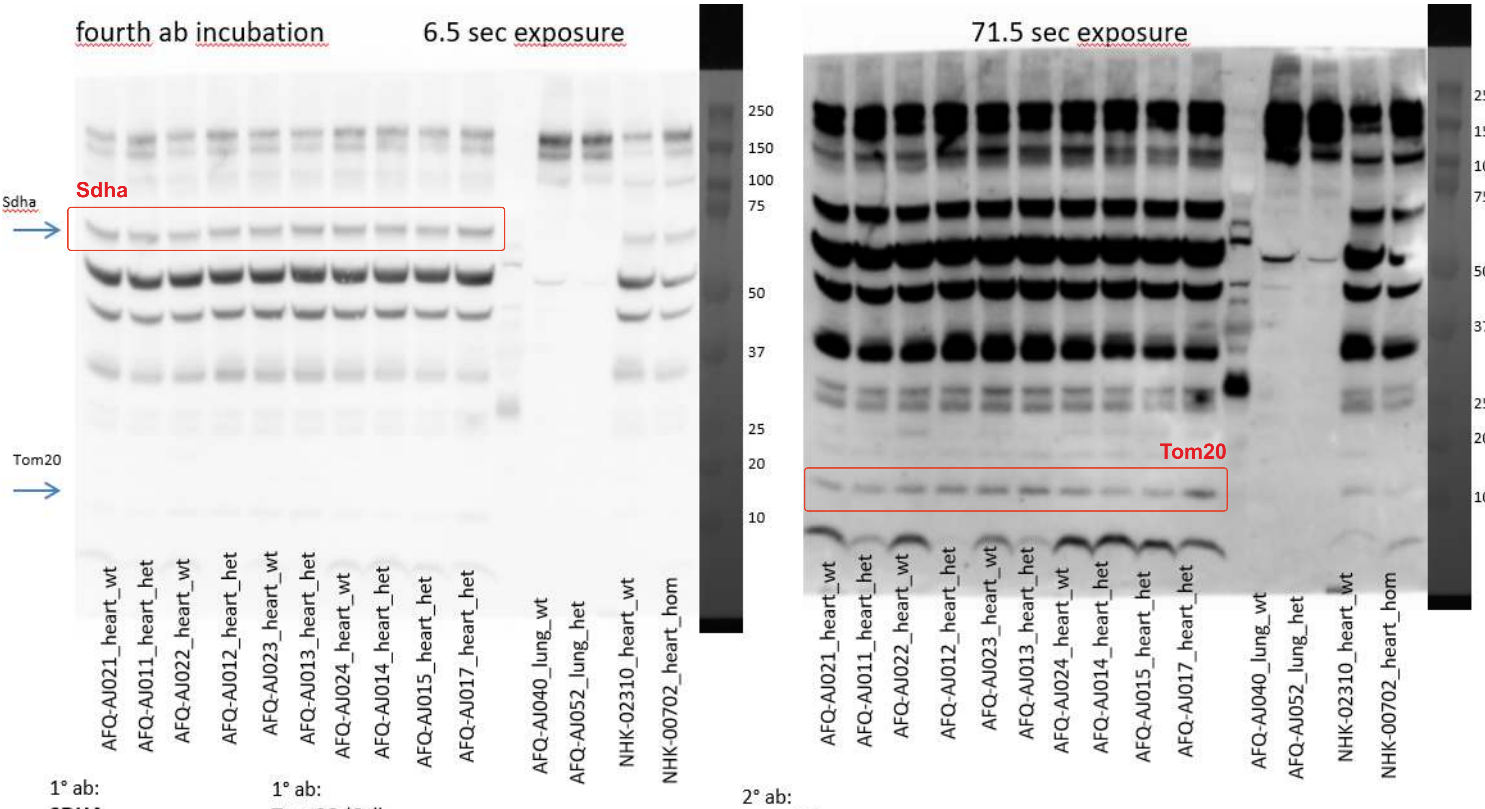

1° ab:
SDHA (Cell Signaling, #11998), 1:1000 in 5% milk/TBST (70 kDa)

1° ab:
Tom20 (Cell Signaling, #42406), 1:1000 in 5% milk/TBST (~16 kDa)

2° ab:
anti-rabbit IgG-HRP (Cell Signaling, #7074P2), 1:1000 in 5% milk/TBST

Supplement Figure 4E-F, page 3
Original Western blots Prdm16csp1/wt, males, 8 months

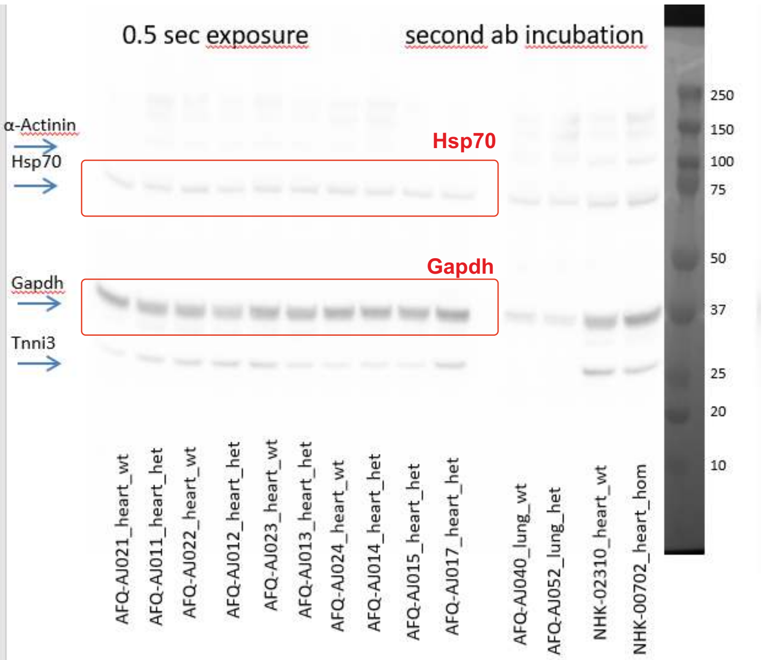

1° ab:
anti-HSP70
(Enzo, ADI-SPA-820), 1:1000 in 5% milk/TBST (72-73 kDa)

1° ab:
anti-GAPDH
(Ambion, AM4300), 1:20000 in 5% milk/TBST (37 kDa)

1° ab:
α-Actinin
(Cell Signaling, #6487), 1:1000 in 5% milk/TBST (100 kDa)

1° ab:
anti-TNNI3
(ThermoFisher, PA[illegible] 28964), 1:1000 in [illegible] milk/TBST (~25 kD[illegible]

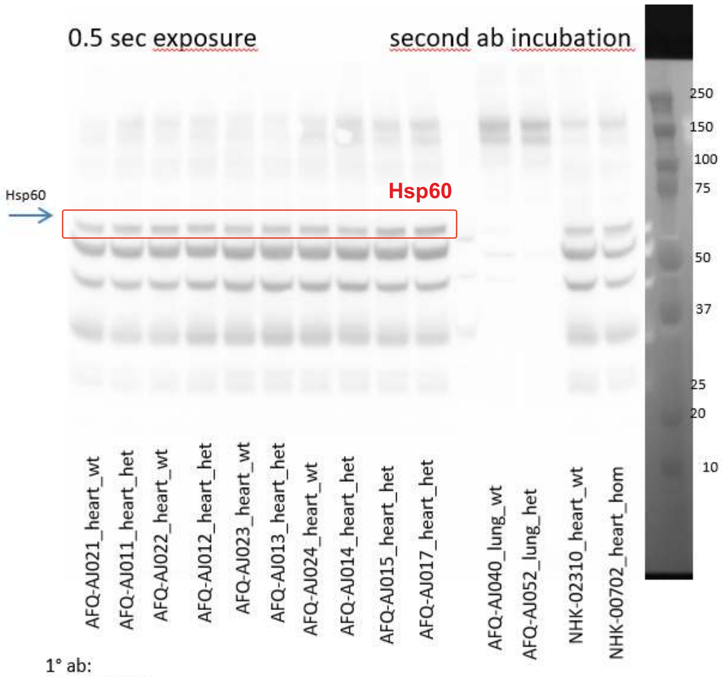

1° ab:
Hsp60 (R&D systems MAB1800) , 62kDa 1:2000 in 5% milk/TBST

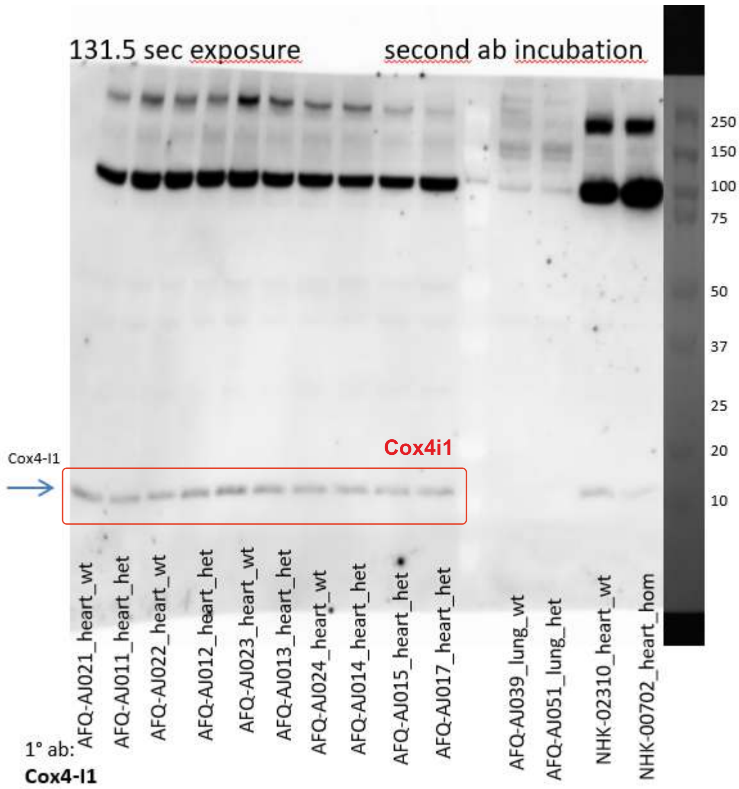

1° ab:
Cox4-I1
(R&D Systems AF5814), 1:200 in 5% milk/TBST (18 kDa)

Supplement Figure 4E-F, page 4
Original Western blots Prdm16csp1/wt, females, 8 months

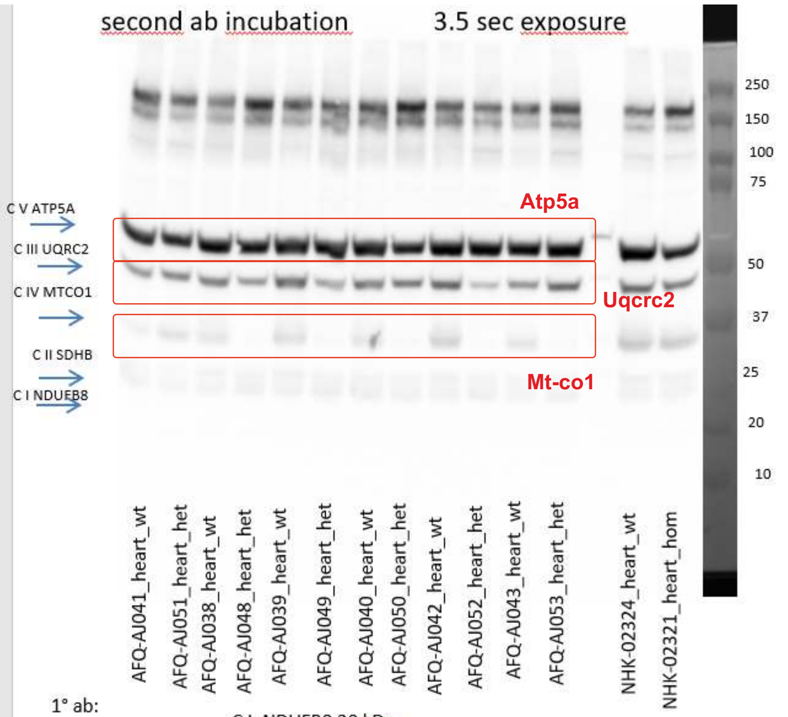

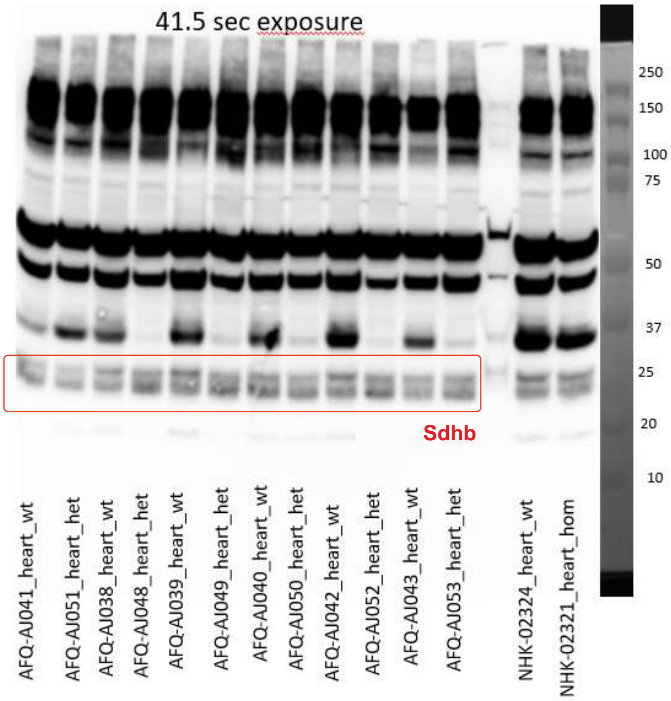

1° ab:
OxPhos Rodent WB Ab cocktail (Invitrogen #45-8099), 1:1000 in 5% milk/TBST

C I: NDUFB8 20 kDa
C II: SDHB 30 kDa
C III: UQRC 48 kDa
C IV: MTCO1 35-40 kDa
C V: ATP5A 55 kDa

2° ab:
anti mouse IgG-HRP (ThermoFisher, #61-8620), 1:1000 in 5% milk/TBST

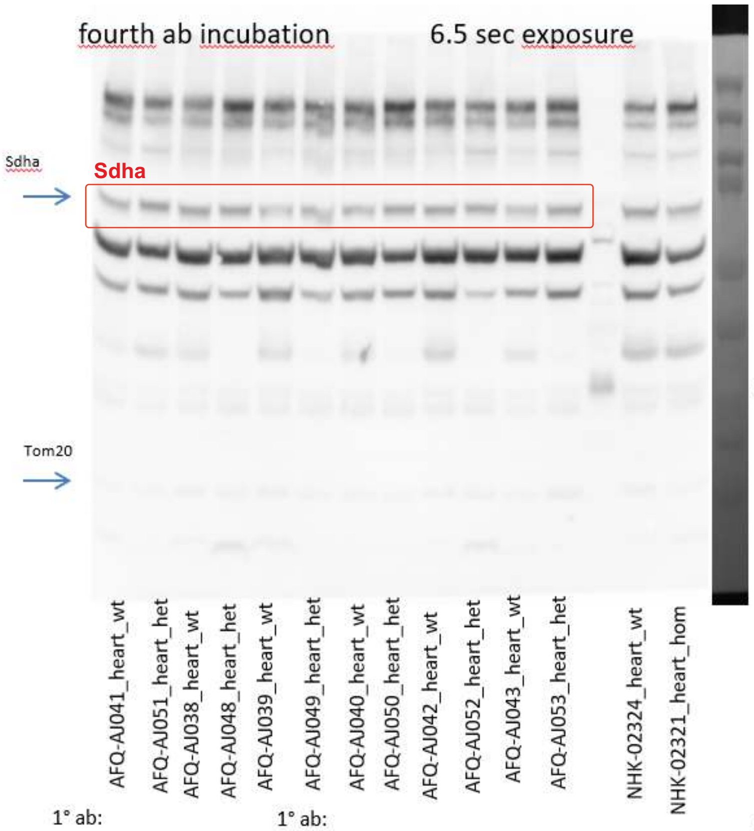

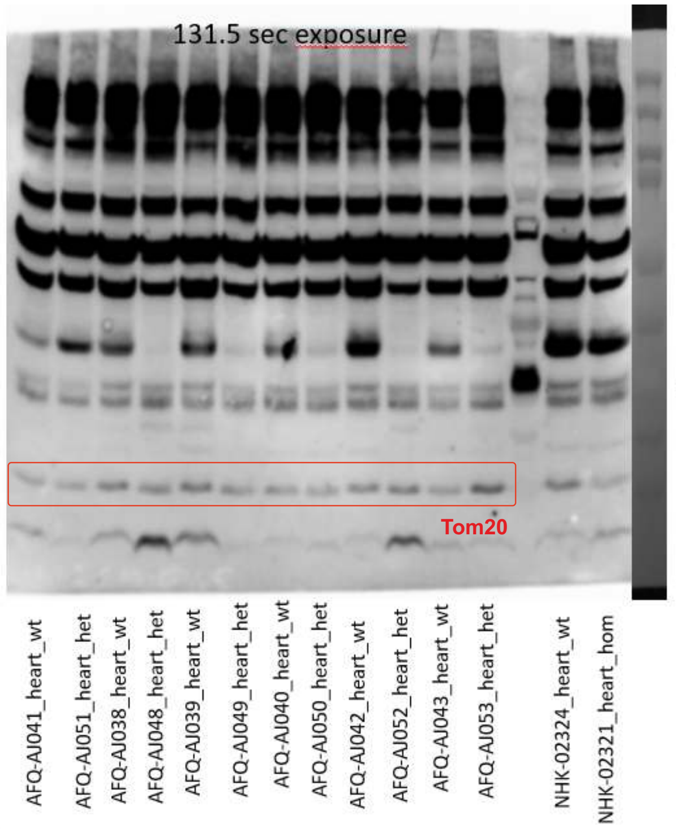

1° ab:
SDHA (Cell Signaling, #11998), 1:1000 in 5% milk/TBST (70 kDa)

1° ab:
Tom20 (Cell Signaling, #42406), 1:1000 in 5% milk/TBST (~16 kDa)

2° ab:
anti rabbit IgG-HRP (Cell Signaling, #7074), 1:1000 in 5% milk/TBST

Supplement Figure 4E-F, page 5
Original Western blots Prdm16csp1/wt, females, 8 months

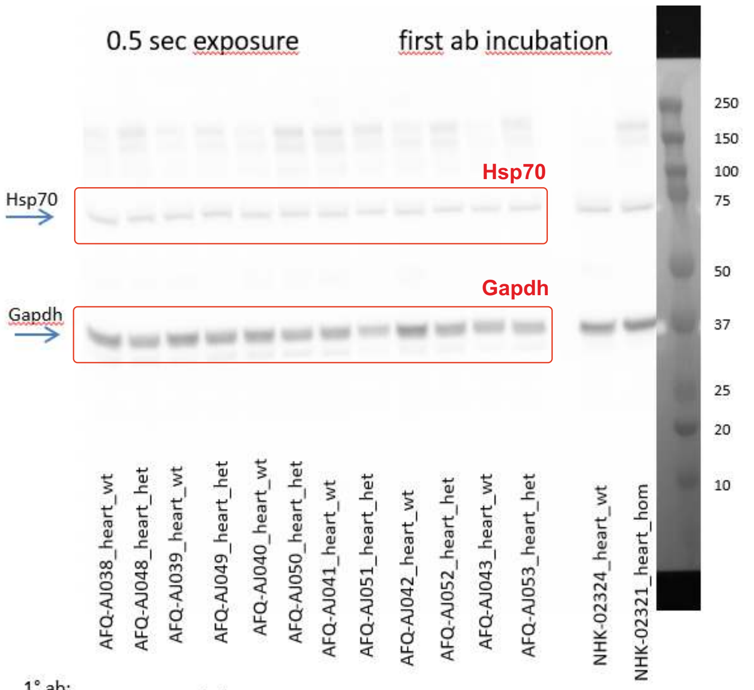

1° ab:
anti-HSP70
(Enzo, ADI-SPA-820), 1:1000 in 5% milk/TBST (72-73 kDa)

1° ab:
anti-GAPDH
(Ambion, AM4300), 1:20000 in 5% milk/TBST (37 kDa)

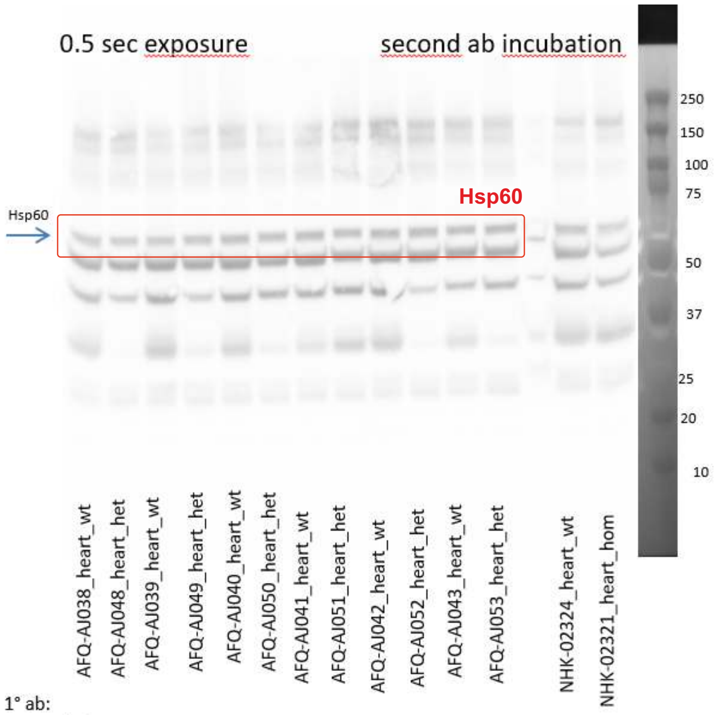

1° ab:
Hsp60 (R&D systems MAB1800) , 62kDa 1:2000 in 5% milk/TBST

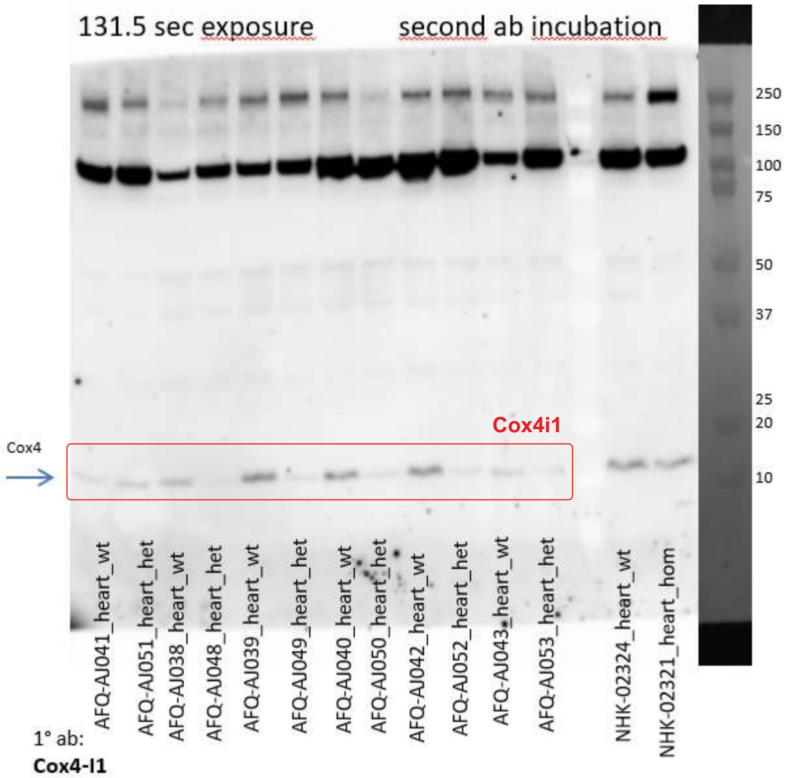

1° ab:
Cox4-I1
(R&D Systems AF5814), 1:200 in 5% milk/TBST (18 kDa)
