# Supplementary material for: Prdm16 mutation determines sex-specific cardiac metabolism and identifies two novel cardiac metabolic regulators
Source: Cardiovasc Res. 2023 Oct 16;119(18):2902–16. doi: 10.1093/cvr/cvad154 (PMC10874277; doi:10.1093/cvr/cvad154)
Supplement: cvad154_Supplementary_Data [file cvad154_supplementary_data.zip › 230704_PRDM16_csp1_supplement_CardiovasRes_revisio_v2.docx]

**Online Data Supplements**

*Prdm16* mutation determines sex-specific cardiac metabolism and identifies two novel cardiac metabolic regulators

Jirko Kühnisch^1,2,3^, Simon Theisen^1,2,3^, Josephine Dartsch^1,2^, Raphaela Fritsche-Guenther^4^, Marieluise Kirchner^5,6^, Benedikt Obermayer^7^, Anna Bauer^4^, Anne-Karin Kahlert^8,9,10^, Michael Rothe^11^, Dieter Beule^2,7^, Arnd Heuser^2^, Philipp Mertins^5,6^, Jennifer A. Kirwan^4^, Nikolaus Berndt^12,13^, Calum A. MacRae^14^, Norbert Hubner^2,3^, Sabine Klaassen^1,2,3,13,15^

*1 Experimental and Clinical Research Center, a cooperation between the Max Delbrück Center for Molecular Medicine in the Helmholtz Association and Charité - Universitätsmedizin Berlin, Germany;*

*2 Max Delbrück Center for Molecular Medicine in the Helmholtz Association (MDC), Berlin, Germany;*

*3 DZHK (German Centre for Cardiovascular Research), partner site Berlin, Berlin, Germany;*

*4 Berlin Institute of Health (BIH) at Charité - Universitätsmedizin Berlin, BIH Metabolomics Platform, Berlin, Germany;*

*5 Max-Delbrück-Center for Molecular Medicine in the Helmholtz Association (MDC), Proteomics Platform, Berlin, Germany;*

*6 Berlin Institute of Health (BIH) at Charité - Universitätsmedizin Berlin, Berlin, Germany;*

*7 Berlin Institute of Health at Charité - Universitätsmedizin Berlin, Core Unit Bioinformatics, Berlin, Germany;*

*8 Department of Congenital Heart Disease and Pediatric Cardiology, University Hospital of Schleswig-*

*Holstein, Kiel, Germany;*

*9 German Center for Cardiovascular Research (DZHK), Kiel, Germany;*

*10 Institute of Immunology and Genetics, Kaiserslautern, Germany;*

*11 Lipidomix GmbH, Berlin, Germany;*

*12 Institute of Computer-Assisted Cardiovascular Medicine, Deutsches Herzzentrum der Charité (DHZC),* *Berlin, Germany*

*13 Charité - Universitätsmedizin Berlin, corporate member of Freie Universität Berlin and Humboldt-Universität zu Berlin, Berlin, Germany*

*14 Harvard Medical School and Cardiovascular Division, Department of Medicine, Brigham and Women’s Hospital, Boston, USA;*

*15 Department of Pediatric Cardiology, Deutsches Herzzentrum der Charité (DHZC),* *Berlin, Germany*

**Methods**

*Mouse breeding and genotyping*

The FVB.C-*Prdm16^csp1^*/J mice were received from Jackson Laboratories, USA (JAX stock #013100). The strain was originally established by N-ethyl-N-nitrosourea (ENU) mutagenesis that generated a missense C>A mutation at the intronic acceptor splice site of exon 7 (DOI: [10.1093/hmg/ddp543](https://doi.org/10.1093/hmg/ddp543)). Viable controls (*Prdm16^wt/wt^*) and heterozygous (*Prdm16^csp1/wt^*) mice were imported from JAX at the age of 2 months and maintained for another 6 months. Maintenance, physiological analysis and organ collection of *Prdm16^csp1/wt^* mice was approved by the Landesamt für Gesundheit und Soziales Berlin (LAGeSo), Germany (G0070/17). All analyses were done at the age of 8 month if not otherwise stated. Body and heart weight were assessed at 8 months. Genotyping of *Prdm16^csp1/wt^* mutant mice occurred according to information at JAX (<https://www.jax.org/strain/013100>). Briefly, a PCR product of 344 bp was generated with an endpoint PCR that was subsequently *HindIII* digested and visualized by agarose gel electrophoresis (Table I in the Data Supplement). Genotyping of *Prdm16^csp1/wt^* mutant mice results, due to generation of a novel *HindIII* cleavage site, in two DNA fragments of 211 bp as well as 133 bp and another DNA fragment of 344 bp from the wild-type allele (Figure I_A-B in the Data Supplement). *Prdm16^wt/wt^* control mice show only the 344 bp fragment.

*Mouse phenotyping*

Phenotyping of *Prdm16^wt/wt^* and *Prdm16^csp1/wt^* mice occurred with echocardiography, electrocardiography, blood pressure measurement, body composition analysis, and respiratory analysis. All animal analyses were approved by the Landesamt für Gesundheit und Soziales Berlin (LAGeSo), Germany (G0070/17). Mice groups were blinded for all phenotyping procedures. Mice with compromised health status were excluded from the analysis according to institutional animal experimental guidelines. We did not exclude mice from analysis. After each analysis, the animals were immediately moved back into the animal facility. The analysis time for all approaches, except for transthoracic echocardiography, was a few minutes. Mice were fed with standard chow composed of crude protein (22.0%), crude fat (4.5%), crude fiber (3.9%), crude ash (6.7%), calcium (1.0%), and phosphorus (0.7%) (V1124-300, ssniff Spezialdiäten GmbH, Germany) and water ad libitum.

For *transthoracic* *echocardiography*, animals were anesthetized by inhaling a 2.5% isoflurane oxygen mixture in a heat-controlled chamber (25x13x12cm) to keep the body temperature stable. The anesthesia chamber was prefilled with the isoflurane oxygen mixture to minimize the time until reaching full anesthesia (approx. 4 min). Chest hair was carefully removed with a clipping machine and depilatory cream. Subsequently, mice were fixed on a heated plate with electrodes on their paws to record the heart rate, respiratory rate, and the electrocardiogram. The body temperature was monitored with a rectal sensor and corrected by a heating lamp if necessary. During analysis, mice continuously inhaled isoflurane oxygen mixture. For ultrasound analysis, the detector MS-550D and the high-resolution imaging system VEVO 2100 was used (Visualsonics Fujifilm, VisualSonics, Canada). The analysis time was 5-10 min. Stroke volume and cardiac output was measured by tracing the endocardium in systole and diastole of a parasternal long axis view of the left ventricle. M-mode and B-mode images were obtained in parasternal short- and long-axis views for measurement of diastolic LV wall thickness (IVSd, LVPWd) and LV systolic function (EF, FS), respectively. The apical 4-chamber view was used to obtain mitral inflow profiles by pulsed wave Doppler (PWD) for estimation of diastolic function (E/A ratio, DTT, IVRT). Diastolic annular velocities (E′, A′) were captured by tissue Doppler imaging (TDI) at the septal mitral annulus. Images were acquired and stored for offline analysis.

*Body composition* analysis was performed to determine the relative amount of body fat, free water, and muscle tissue. Measurement of mice occurred with an NMR-spectroscopic method (Bruker LF90II, Germany) in a shaded, tubular, and tempered analysis chamber (diameter 5.5 cm). Non-invasive measurement of systolic and diastolic *blood pressure* was performed with the CODA tail-cuff system (Kent Scientific, CT, USA). The analysis occurred in a shaded, tubular, and tempered analysis chamber. For *electrocardiography* the ecgTUNNEL system (EMKA Technologies, France) including a shaded chamber was used. The measurement chamber was equipped with four electrodes at the bottom that infer electrocardiographs via the mouse paws.

*Molecular biology and transcriptome analysis*

The primer for generation of PCR products, DNA sequencing, and quantitative PCR (qPCR) are available online (Table I in the Data Supplement).

Transcript levels were measured with *qPCR* from cDNA that was synthesized from total RNA isolates. Total murine RNA was isolated from liquid nitrogen crashed and powdered heart tissue with TRIzol reagent (Invitrogen, USA). Human RNA isolates were purchased (Clontech, USA). For mRNA expression analysis, total RNA was isolated according to standard protocols and transcribed into cDNA with SuperScriptII (Invitrogen, USA). Expression analysis was performed with quantitative PCR (qPCR) on a 7500 Real Time PCR System (Applied Biosystems, USA) using *GAPDH, Gapdh* as endogenous control. The relative expression was calculated according to the ΔΔCT method.

*Sanger sequencing* was performed according to standard laboratory protocols. Nucleotide numbering reflects cDNA position with +1 corresponding to the A of the ATG translation initiation codon in the reference sequence, according to standard guidelines ([www.hgvs.org/mutnomen](https://exchange.charite.de/owa/redir.aspx?C=89ee8ec0056f402ca0e51ed25d435ec5&URL=http%3a%2f%2fwww.hgvs.org%2fmutnomen)). The initiation codon is codon 1. For murine *Prdm16* the following reference gene and transcript sequences were used ENSMUSG00000039410 and [ENSMUST00000030902.12](http://www.ensembl.org/Mus_musculus/Transcript/Sequence_cDNA?db=core;g=ENSMUSG00000039410;r=4:154316125-154636866;t=ENSMUST00000030902), respectively.

*Targeted amplicon high-throughput sequencing* was performed from gel eluted PCR products that underwent Bioanalyzer quantification (Genewiz, Germany). We first took raw sequencing reads and calculated fragment length for overlapping read pairs by pairwise alignment using the globalms method in the pairwise2 python package with penalties 5, -2, 5, 1. Reads were aligned to the mouse genome (GRCm38) using STAR (v2.7.3a) with parameters --outFilterType BySJout --outFilterMultimapNmax 20 --alignSJoverhangMin 8 --outSJfilterOverhangMin 12 12 12 12 --outSJfilterCountUniqueMin 1 1 1 1 --outSJfilterCountTotalMin 1 1 1 1 --outSJfilterDistToOtherSJmin 0 0 0 0 --outFilterMismatchNmax 999 --outFilterMismatchNoverReadLmax 0.04 --scoreGapNoncan -4 --scoreGapATAC -4 --chimOutType WithinBAM HardClip --chimScoreJunctionNonGTAG 0 --alignSJstitchMismatchNmax -1 -1 -1 -1 --alignIntronMin 20 --alignIntronMax 1000000 --alignMatesGapMax 1000000 --outSAMunmapped Within --genomeLoad NoSharedMemory --outSAMattributes NH HI AS NM MD --outSAMtype BAM Unsorted --peOverlapNbasesMin 20 --peOverlapMMp 5. Alignments within the *Prdm16* locus for fragments with length between 235 nt and 255 nt were inspected for gaps of more than 50 nt.

*High-throughput mRNA expression analysis (RNAseq)* was performed from mRNA preparations derived from heart tissue total RNA isolates. Raw reads were aligned with *STAR* (v2.6.1a) against the GRCm38 reference and quantified using featureCounts (v1.6.3) with the Gencode vM12 annotation. Differential expression analysis was performed in R (v3.4.1) using DESeq2 (v1.18.1) (<https://doi.org/10.1186/s13059-014-0550-8>). The technical quality of RNAseq data was assessed for the number of uniquely, multiple and unmapped reads. Scatterplots illustrate the LFC against mean expression for the contrast *Prdm16^csp1/wt^* (het) vs. controls (wt) in male (left) or female (right) animals. The top 10 differentially regulated genes (adj. p < 0.01) are highlighted. Identified transcripts were validated for their cardiac cell specific expression (<https://www.proteinatlas.org/>), protein (<https://www.uniprot.org/>), and gene ontology (quick GO, <https://www.ebi.ac.uk/QuickGO/>). RNAseq data were tested for dysregulation of cardiac disease genes (harmonizome gene set *congenital heart disease*, panel comprises 910 genes important for heart disease and function; DOID 1682; https://maayanlab.cloud/static/hdfs/harmonizome/data/jensendiseasecurated/gene_set_library_crisp.gmt.gz downloaded on Sep 10 2018). A gene ontology (GO) network was constructed using the TOP20 regulated genes from male and female *Prdm16^csp1/wt^* mice using GOnet (<https://tools.dice-database.org/GOnet/>). Construction settings were q-value <0.05, mouse reference data base, GO term enrichment, and a heart transcript expression data base (Bgee) as reference.

*Protein analysis and proteomics*

*Immunostaining* was performed on heart tissues sections fixed with 4% paraformaldehyde, embedded in paraffin and sectioned with a microtome (Leica, Germany). Primary and secondary antibodies used for immunostaining are given online (Table II in the Data Supplement). For immunofluorescence analysis, tissue sections were probed with the designated primary antibodies that were subsequently probed with appropriated secondary antibodies. Nuclei and plasma membranes were stained with DAPI Alexa Fluor 405 and wheat germ agglutinin (WGA) Alexa Fluor 488, respectively. Imaging of immunofluorescence staining occurred with a four-channel laser scanning microscope (LSM700, Zeiss, Germany) under identical conditions.

*A bead-based multiplex immunoassay* was performed with protein preparations generated from heart tissue samples. Heart tissue samples were mechanically crushed following freezing with liquid nitrogen. Protein lysates were obtained by incubation with a cell lysis buffer (Merck Millipore Burlington, MA, USA). Protein phosphorylation was assayed with the AKT/mTOR Phosphoprotein Magnetic Bead Kit (41-611MAG, Merck Millipore Burlington, MA, USA) according to the manufacturer’s recommendations. Pooled quality control (QC) samples were used as a quality control (n=5 spread over the plate) to determine the instrument variance. The xPONENT 4.2 software (Merck Millipore Burlington, MA, USA) was used for acquisition of data. For background substraction, phosphatase treated protein lysates from HeLa cells were used. A minimum mean fluorescent intensity of 30 counts per analyte was applied. Sample values were normalized with β-tubulin (Merck Millipore Burlington, MA, USA) as internal standard. Statistical significance was tested with unpaired Students t-Test, p<0.05. Detection of mouse Brain Natriuretic Peptide (BNP) occurred with ELISA (abbexa, #abx255217) using plasma sample from *Prdm16^csp1/wt^* mice according to manufactures instructions.

*Global proteomes* of *Prdm16^csp1/wt^* and control cardiac heart tissue were measured and analyzed by mass spectrometry-based proteomics, using label-free liquid chromatography-mass spectrometry (LC/MS-MS). The protein containing interphase from lipid extraction was pelleted, air dried, and solubilized in SDC lysis buffer [1% Sodium deoxycholate, 150 mM NaCl, 50 mM Tris-HCl pH 8, 1 mM EDTA, 10 mM DTT (dithiothreitol, Sigma), 40 mM CAA (2-chloroacetamide, Sigma)]. Samples were sonicated for 10 seconds, incubated at 95°C for 10 min, cooled down and treated with Benzonase® (Merck, 50 units) for 15 min at 37°C. Protein concentrations were determined using the Pierce BCA assay. 50 μg of protein extract per sample was digested with 1 μg endopeptidase LysC (Wako) and 1 μg sequence-grade trypsin (Promega) overnight at 37°C. The digestion was stopped by adding trifluoroacetic acid (final concentration 1%) and peptides were desalted and cleaned up using stage tip purification protocol (DOI:[10.1021/ac026117i](https://doi.org/10.1021/ac026117i)). For LC/MS-MS measurements, peptides were reconstituted in 3% acetonitrile with 0.1% formic acid and separated on a reversed-phase column [20 cm fritless silica microcolumns with an inner diameter of 75 µm, packed with ReproSil-Pur C18-AQ 1.9 µm resin (Dr. Maisch GmbH)] using a 202 min gradient with a 250 nl/min flow rate of increasing Buffer B (90% ACN, 0.1% FA) concentration (from 2% to 60%) on a High Performance Liquid Chromatography (HPLC) system (Thermo Fisher Scientific) and analyzed on a Q-Exactive HF-X instrument (Thermo Fisher Scientific). The mass spectrometer was operated in data-dependent acquisition mode using the following settings: full-scan automatic gain control (AGC) target 3e6 at 60,000 resolution; scan range 350–1800 m/z; Orbitrap full-scan maximum injection time 10 ms; MS2 scan AGC target 1e5 at 15,000 resolution; maximum injection time 22 ms; dynamic exclusion time 30 s; isolation window 1.3 m/z; precursor charge state 2–6, 20 MS2 scans per full scan. Each biological sample was analyzed in technical duplicates. Raw data were processed using MaxQuant software package (v1.6.3.4, DOI:[10.1038/nprot.2016.136](https://doi.org/10.1038/nprot.2016.136)) and a decoy human UniProt database (MOUSE.2019-07), containing forward and reverse sequences. The search included variable modifications of oxidation (M), N-terminal acetylation, deamidation (N and Q), and fixed modification of carbamidomethyl cysteine. Minimal peptide length was set to seven amino acids and a maximum of three missed cleavages was allowed. The FDR was set to 1% for peptide and protein identifications. Unique and razor peptides were considered for quantification. MS2 identifications were transferred between runs with the “Match between runs” option. The integrated LFQ (label-free) quantitation algorithm was applied. Reverse hits, contaminants and proteins only identified by site were filtered out and statistical data analysis was performed using the Perseus software (v 1.6.2.1). Biological replicates for each condition were defined as groups and a minimum of three values per group was required. Missing values were imputed with low intensity values simulating the detection limit of the mass spectrometer. Differences in protein abundance between the groups were calculated using the two-sample Student t-test.

*For PRM (Parallel Reaction Monitoring) analysis* of Prdm16, cryo-pulverized lung tissue (higher Prdm16 expression) was used. Protein extraction and tryptic digest was performed as described for heart tissue samples (see above; global proteomics). Peptide were reconstituted in 3% acetonitrile with 0.1% formic acid, isotope labeled TQL peptides (8 fmol, JPT Inc., Berlin, Germany) were spiked in and the samples were separated on a reversed-phase column [20 cm fritless silica microcolumns with an inner diameter of 75 µm, packed with ReproSil-Pur C18-AQ 1.9 µm resin (Dr. Maisch GmbH)] using a 98 min gradient with a 250 nl/min flow rate of increasing Buffer B (90% ACN, 0.1% FA) concentration (from 2% to 60%) on a High Performance Liquid Chromatography (HPLC) system (Thermo Fisher Scientific). The mass spectrometer (Q-Exactive HF-X instrument; Thermo Fisher Scientific) was operated in PRM mode using the following settings: automatic gain control (AGC) target 2e5 at 30,000 resolution; isolation window 1.6m/z, NCE of 28; maximum injection time 100ms. The isolation list contained the light (endogenous) and heavy (isotope-labeled) targets from histone 2a/x (AGLQFPVGR; charge state 2+) and Prdm16 specific control sequence (V875-K883; VADPVGVLK; charge state 2+). Skyline software package (DOI:[10.1093/bioinformatics/btq054](https://doi.org/10.1093/bioinformatics/btq054)) was used for PRM assay development (retention time, charge state, collision energy NCE, and transitions of isotope labeled TQL peptides) and data analysis. Light to heavy peptide ratios were used for the quantitation analysis. Histone 2a/x ratios were used for normalization.

*Metabolic analyses*

For *sample preparation and metabolite extraction* mouse heart tissue samples (left ventricle, 30 mg), were homogenized using a Precellys FastPrep24 Tissue Homogenizer (Bertin Instruments, Montigny-le-Bretonneux, France). The tissue was lysed by the addition of 1 ml per 30 mg tissue methanol (MeOH):chloroform (CHCl_3_):water (H_2_O) (5:2:1, *v/v/v*) mixture, followed by incubation for 60 min at 4°C with shaking. Next, 500µl/30mg of tissue of H_2_O was added, followed by incubation for 10 min at 4°C with shaking. The vials were centrifuged at 18,213 x g at 4°C. After extraction, 300µL of polar phase was dried at 30°C at a speed of 1,550 x g at 0.1mbar using a rotational vacuum concentrator (RVC 2-33 CDplus, Christ, Osterode am Harz, Germany). A representative quality control (QC) sample was made by pooling and aliquot from each of the samples. QC samples were prepared alongside the samples in the same way and were used to test the technical variability of the instrument.

*Central carbon pathway metabolites were measured with gas chromatography-mass spectrometry (GC-MS).* All polar tissue extracts were stored dry at −80°C until analysis. The extracts were removed from the freezer and dried in a rotational vacuum concentrator for 60 min before further processing to ensure there was no residual water which may influence the derivatization efficiency. The dried extracts were dissolved in 15 µL of methoxyamine hydrochloride solution (40 mg/mL in pyridine) and incubated for 90 min at 30°C with constant shaking, followed by the addition of 50 µL of N-methyl-N-[trimethylsilyl]trifluoroacetamide (MSTFA) and incubated at 37°C for 60 min. The extracts were centrifuged for 10 min at 18,213 x *g*, and aliquots of 25 µL were transferred into glass vials for GC-MS measurements. An identification mixture for reliable compound identification was prepared and derivatized in the same way, and an alkane mixture for a reliable retention index calculation was included (DOI:[10.3390/metabo10110457](https://doi.org/10.3390/metabo10110457)). Analysis of the central carbon metabolites was performed on a Pegasus 4D GCxGC TOFMS-System (LECO Corporation, St. Joseph, MN, USA) complemented with an auto-sampler (Gerstel MPS DualHead with CAS4 injector, Mühlheim an der Ruhr, Germany). The samples were injected in split mode (split 1:5, injection volume 1 µL) in a temperature-controlled injector with a baffled glass liner (Gerstel, Mühlheim an der Ruhr, Germany). The following temperature program was applied during the sample injection: for 2 min, the column was allowed to equilibrate at 68°C, then the temperature was increased by 5°C/min until 120°C, then by 7°C/min up to 200°C, then by 12°C/min up to a maximum temperature of 320°C, which was then held for 7.5 min. The gas chromatographic separation was performed on an Agilent 7890 (Agilent Technologies, Santa Clara, CA, USA), equipped with a VF-5ms column (Agilent Technologies) of 30 m length, 250 µm inner diameter, and 0.25 µm film thickness. Helium was used as the carrier gas with a flow rate of 1.2 mL/min. The spectra were recorded in a mass range of 60 to 600 m/z with 10 spectra/second. The GC-MS chromatograms were processed with the ChromaTOF software version 4.72.0.0 (LECO Corporation, St. Joseph, MN, USA) including baseline assessment, peak picking and computation of the area of peaks without a calibration by using an in-house created reference and library containing the top 3 masses by intensity. Data were manually adjusted to assume correct peak annotation and integration.

To *assess the central carbon metabolism*, measured GC-MS raw value were exported and merged by an in-house written R script. 42 targeted metabolites from the central carbon metabolism covering glycolysis, tricarboxylic cycle, amino acids, nucleobases/nucleosides, pentose phosphate pathway, short chain fatty acids, sugar alcohols, carboxylic acids, and glycerol pathway were selected for the reference search. Microsoft Excel (Microsoft Excel 2013 15.0.5327.1000) and R Studio (version 1.3.1056) were used for data analysis and Adobe Illustrator (version 16.0.0) was used for visualization. The metabolites were considered valid when they appeared in minimum of n=3 replicates per condition. The peak area of each metabolite was calculated by normalization to the internal standard cinnamic acid. The measured derivatives were summed up. Outlier analysis was done using Grubbs test (<https://www.graphpad.com/quickcalcs/Grubbs1.cfm>). Using the mean of the biological replicates, the log2 ratio *Prdm16^csp1/wt^*/control mice for metabolites were calculated. Statistical analysis was performed using the non-parametric Wilcoxon Rank Sum test (p ≤0.05).

*Univariate analysis of the central carbon metabolites* was performed to assess global changes in specific metabolite classes, a way of combining results of all metabolites belonging to a specific class. First, univariate scaling of the normalized peak areas was performed for each metabolite. A transformation was then performed on each individual value by subtracting the value (-mean/sd). This value is equivalent to the univariate scaled log2 ratio of a 1:1 ratio such that zero represents a 1:1 ratio on the plot between male and female or control to basal. As each metabolite is measured on the same scale, the results can be pooled and considered together. Metabolites with higher peak areas are positive values on the plot, negative values represent lower concentrations. We used the Pearson`s chi-squared test to perform a chi-squared contingency table test and goodness-of-fit with a threshold of p<0.05 for statistically relevant differences between the metabolic groups and the total number of metabolites.

Measurement of *metabolites reflecting the energy and redox status* in heart tissue was performed with LC-MS (MS-Omics, [www.msomics.com](http://www.msomics.com)). For these metabolites, heart tissue extracts from *central carbon metabolism* were analyzed. The analysis was carried out using a Thermo Scientific Vanquish LC coupled to Thermo Q Exactive HF MS. An electrospray ionization interface was used as ionization source. Standard analysis was performed in positive ionization mode. Adapted analysis of molecules >300 g/mol was performed in positive ionization mode with the m/z range limited to 200-1500 amu. The UPLC was performed using a slightly modified version of the protocol described Hsiao et al. 2018 (DOI:[10.1021/acs.analchem.8b02100](https://doi.org/10.1021/acs.analchem.8b02100)). Peak areas were extracted using Compound Discoverer 3.1 (Thermo Scientific). Identification of compounds were performed at four levels; Level 1: identification by retention times (compared against in-house authentic standards), accurate mass (with an accepted deviation of 3ppm), and MS/MS spectra, Level 2a: identification by retention times (compared against in-house authentic standards), accurate mass (with an accepted deviation of 3ppm). Level 2b: identification by accurate mass (with an accepted deviation of 3ppm), and MS/MS spectra, Level 3: identification by accurate mass alone (with an accepted deviation of 3ppm). Data processing occurred with Skyline by manual integration of compounds (DOI:[10.1021/acs.jproteome.9b00640](https://doi.org/10.1021/acs.jproteome.9b00640)).

*Lipid profiling* of heart tissue was performed with liquid chromatography-mass spectrometry (LC-MS) using the Lipidyzer kit from Sciex (AB Sciex LLC, Framingham, MA, USA). The following lipid classes were analyzed: cholesterol Ester (CE), ceramides (CER), diacylglycerol (DAG), dihydroceramides (DCER), free Fatty Acids (FFA), hexosylceramides (HCER), lactosylceramide (LCER), lysophosphatidylcholine (LPC), lysophosphatidylethanolamine (LPE), phosphatidylcholine (PC), phosphatidylethanolamine (PE), sphingomyelin (SM), and triacylglycerol (TAG). Overall, 1070 lipids including 58 internal standards were measured. Sample preparation was carried out according to a modified manufacturer’s Lipidyzer protocol adapted for tissues and which substituted dichloromethane with chloroform. Briefly, 25 mg of tissue sample were homogenized with a tissuelyser under liquid nitrogen in ice-cold 4 mL methanol:water mixture with cinnamic acid as internal standard for further polar metabolite analysis. Two step extraction was carried out with 2 mL chloroform after adding internal standards. After incubation at 4°C for 30 min, a centrifugation step and a second extraction with chloroform, polar phase (1 mL) and lipid phase (2 mL) were transferred respectively into new Eppendorf and glass tubes and dried in a rotational vacuum concentrator (RVC 2-33 CDplus, Christ, Osterode am Harz, Germany) at 30°C. Afterwards, the lipid phase was reconstituted in 560 µL running solvent (50:50, v/v methanol:dichloromethane with 10 mM ammonium acetate) and centrifuged just prior to analysis.

The LC-MS system for lipid detection was composed of a Shimdazu Nexera X2 UHPLC-System (Schimdazu, Kyoto, Japan) coupled to a QTRAP®System with SelexION® Differential Mobility Spectrometry (DMS) Technology (AB Sciex LLC, Framingham, MA, USA). Two methods were used covering thirteen lipid classes using a flow injection analysis (FIA); one injection with SelexION Technology ON and another with the SelexION Technology turned OFF. A flow injection analysis (FIA) setup is employed by using the LC to flow at an isocratic rate of 7 μL/min with a ramp up to 30 μL/min for the last 2 min of the experiment to allow washing. Data acquisition is around 20 min per sample. 50 μL of each reconstituted sample was injected and analyzed with the recorded peak intensities normalized to the appropriate internal standard. 20 spectral scans were collected for each lipid per run. The lipid molecular species were measured using multiple reaction monitoring (MRM) and positive/negative switching. Positive ion mode detected the following lipid classes SM, DAG, CE, CER, and TAG. Negative ion mode detected the following lipid classes LPE, LPC, PC, PE, and FFA. Samples were quantified using the Lipidomics Workflow Manager (LWM). The kits include unlabeled internal standards for the compensation voltage (COV) tuning of the SelexION® device, control lyophilized plasma used as a QC sample, and QC spike samples (defined lipid amount added to control plasma). A pooled QC sample was prepared by pooling an aliquot from each of the test samples after extraction (five samples were not represented in the pooled QC due to low sample volumes). The QC samples were analyzed separately as technical replicates to remove lipids with large technical variation from the analysis. Blank (water) samples were included to monitor contamination/carry over and solvent blank (mobile phase) for equilibration of the system before analysis. A system suitability test for system performance measurement was carried out as a comprehensive test according to Sciex instructions prior to the analysis of the samples. Samples are quantified using the LWM software which reports all the detected lipids in nmol/g. Data analysis and statistics were conducted using Microsoft Excel 2016, Metabonalyst (https://www.metaboanalyst.ca), Matlab 2018a (The Mathworks, USA) with PLStoolbox v. 8.9 (Eigenvector Research Inc., USA).

The Lipidyzer workflow manager was used for the quantification and identification of the detected lipids. A cut off for relative standard deviation (RSD) was determined (mean RSD ± SD) at 15 %. Lipids with RSD above 15 % in QC pool samples were excluded from further analysis. Lipids were valid when they appeared in a minimum of 3 out of 4 pooled QC samples. Lipids with over 20% missing values were excluded. 640 lipids (60% of the target species) remained following this quality control step. The following lipids were validly detected: CE n=10 (38%), CER n=7 (58%), DAG n=38 (64%), DCER n=4 (33%), FFA n=26 (100%), HCER n=3 (25%), LCER n=0, LPC n=11 (42%), LPE n=7 (27%), PC n=52 (37%), PE n=72 (33%), SM n=12 (100%), and TAG n=398 (81%). The Lipidomics Workflow Manager automatically groups lipids into lipid classes. Wilcoxon Rank Sum tests were conducted on the individual data points to calculate the significance of any changes in lipid classes using this class-based data. Using the mean value of the biological replicates, the log2 ratio *Prdm16^csp1/wt^*/control mice for accumulated lipid classes were calculated. Differential analysis for male and female mice was performed. Statistical analysis was performed using the non-parametric statistical Wilcoxon Rank Sum test (p≤0.05) with a Benjamini-Hochberg FDR correction (q≤0.1). Individual lipids were statistically analyzed using the non-parametric Wilcoxon Rank Sum test (p≤0.05).

*Eicosanoid analysis* was performed using a standard extraction and analysis protocol (Lipidomix, Berlin, Germany). Profiling of eicosanoids was performed as reported (DOI:[10.1194/jlr.M047357](https://doi.org/10.1194/jlr.m047357)). Briefly, heart tissue was mechanically crashed using liquid nitrogen. The pulverized tissue was extracted using isopropanol. The extract was subsequently hydrolyzed with alkaline hydrolysis with 300 µl of 10 M sodium hydroxide (NaOH) at 60°C for 30 min. The sample was then adjusted to pH 6 using 300 µl 58% acetic acid. The prepared samples were then subjected to solid phase extraction (SPE) using a Varian Bond Elut Certify II column. Specific experimental steps were described as previously (DOI:[10.1194/jlr.M047357](https://doi.org/10.1194/jlr.m047357)). The extracted metabolites were evaluated by LC-MS/MS using an Agilent 6495 Triple Quad mass spectrometer (Agilent Technologies, Santa Clara, CA, USA) and an Agilent 1290 high-performance liquid chromatography (HPLC) system (binary pump, multi sampler, thermostatic column oven). A Zorbax Eclipse plus column (150 mm, 2.1 mm, 1.8 m; Agilent Technologies, Santa Clara, CA, USA) was used in the HPLC system. The specific analysis process has been described previously (DOI:[10.1194/jlr.M047357](https://doi.org/10.1194/jlr.m047357)).

*Evaluation of metabolic capacities was performed with CARDIOKIN1.* CARDIOKIN1 is a fully kinetic model of cardiac energy metabolism (DOI:[10.1161/CIRCULATIONAHA.121.055646](https://doi.org/10.1161/circulationaha.121.055646)). It comprises the pathways involved in the catabolism of the energy-delivering substrates glucose, lactate, fatty acids, ketone bodies (kb), and branched chain amino acids (bcaa). The model considers the regulation of metabolic enzymes and transporters by substrate affinities, allosteric regulations as well as interconversion (phosphorylation) in response to insulin and catecholamines. The interdependence between plasma glucose and insulin concentration was considered by using a sigmoid Hill-type function describing the experimentally determined glucose-insulin relations (DOI:[10.1186/s12915-016-0237-6](https://doi.org/10.1186/s12915-016-0237-6)). Individual instantiations of the model were obtained by using the protein intensity profiles delivered by quantitative shotgun proteomics to scale the maximal activities of enzymes and transporters, as described in Berndt et al. (DOI:[10.1038/s41467-018-04720-9](https://doi.org/10.1038/s41467-018-04720-9)), exploiting the fact that the maximal activity of an enzyme is proportional to the abundance of the enzyme protein according to the relation:

$$v_{max}^{sample}=v_{mx}^{mean control}\frac{E^{sample}}{E^{mean control}}$$

The maximal activities $v_{mx}^{mean control}$for the normal were obtained from (DOI:[10.1161/CIRCULATIONAHA.121.055646](https://doi.org/10.1161/circulationaha.121.055646)). $E^{mean control}$ denotes the mean protein abundance in the control group, and $E^{sample}$ denotes the protein abundance of enzyme E in

the other metabolites constant. The external conditions for the different metabolic functions sample.

Metabolic capacities were assessed by monitoring the magnitude of the different metabolic functions in response to changes in the concentration of the respective metabolites, while keeping are given in Berndt et al. (DOI:[10.1161/CIRCULATIONAHA.121.055646](https://doi.org/10.1161/circulationaha.121.055646)). Energetic capacities were assessed by successively increasing the ATP consumption rate under two physiological conditions, a postprandial state with high glucose and low fatty acid concentrations and a fasted state with low glucose and high fatty acid concentrations (DOI:[10.1161/CIRCULATIONAHA.121.055646](https://doi.org/10.1161/circulationaha.121.055646)).

Validation was performed by comparing simulation results with experimental data covering the central metabolic functions of utilization of carbohydrates (glucose, lactate), fatty acids, ketone bodies and branched chain amino acids at varying substrate concentrations and hormonal stimulation. In addition to correct capitulation of metabolic fluxes, the concentration values of internal metabolites were checked to be in the experimentally determined range. For a detailed overview including reference to the experimental data used see:

<https://icm.charite.de/fileadmin/user_upload/microsites/m_cc11/icic/AG_Berndt/Supplemental_Materials_CARDIOKIN_v3_full_ref.pdf>

*Histology and electron microscopy*

For *histological analysis and immunostaining* heart tissue was fixed in buffered 4% paraformaldehyde and subsequently dehydrated in ascending ethanol steps and cleared in xylene. Embedding occurred in paraffin and 4 µm sections were generated with a rotary microtome. Picro-Sirius Red staining was used for the detection of collagen depositions and heart tissue structure was assessed using hematoxylin/eosin (H&E) staining. Imaging of stained sections occurred with a bright light Pannoramic MIDI II slide scanner (Sysmex, Europe) followed by qualitative analysis for fibrotic plaques and structural alterations. For immunofluorescence analysis, tissue sections were probed with primary antibodies (Table II in the Data Supplement). Nuclei and plasma membranes were stained with 4′,6-diamidino-2-phenylindole (DAPI) Alexa Fluor 405 and wheat germ agglutinin (WGA) Alexa Fluor 488, respectively. Imaging of immunofluorescence staining occurred with a four-channel laser scanning microscope (LSM700, Zeiss, Germany) under identical conditions. Qualitative and quantitative analysis of confocal images occurred with ZEN 3.0 (Zeiss, Germany).

For *transmission* *electron microscopy* (TEM) heart tissue was fixed for at least 2 h at 4°C in 3% glutaraldehyde solution in 0.1 M cacodylate buffer, pH 7.4. Tissue was washed in buffer, postfixed for 1 h at 4°C in 1% osmium tetroxide, rinsed in water, and dehydrated through graded ethanol solutions. After transfer into propylene oxide and embedding in epoxy resin (glycidether100), ultrathin sections were cut with an ultramicrotome (Reichert Ultracut E). TEM images were achieved with an electron microscope (Zeiss, Leo EM 906). A macro for AxioVison (Zeiss, Germany) was used to quantify sarcomere and mitochondrial areas in TEM images. Measurement of the sarcomere and mitochondria area/number was normalized to the defined region of interest (ROI).

*Statistical analysis*

Statistical analysis was performed with unpaired t-test or Welch-test. Graphs show the mean and standard deviation. Statistical analysis performed for individual techniques are described there.

**Results**

*Molecular analysis of the genetic defect of mice*

We reassessed the genetic mechanism in *Prdm16^csp1/wt^* mice leading to *Prdm16* inactivation on genetic and transcriptome level. PCR genotyping and Sanger sequencing confirmed presence of the c.888-3C>A (ENSMUSG00000039410) variant on genetic level (Figure 1D, Figure I_A-B in the Data Supplement). To further validate the impact of the *Prdm16* acceptor splice site variant c.888-3C>A ([ENSMUST00000030902.12](http://www.ensembl.org/Mus_musculus/Transcript/Sequence_cDNA?db=core;g=ENSMUSG00000039410;r=4:154316125-154636866;t=ENSMUST00000030902)) on transcriptional level, we performed PCR and targeted high throughput sequencing of total RNA isolated from different *Prdm16^wt/wt^* and *Prdm16^csp1/wt^* tissues. By endpoint PCR (exon_3-9) using cDNA from heart and lung we observed in heterozygous *Prdm16^csp1/wt^* mice three PCR products: the control allele of 807 bp (a), the known exon 7 skipping mutant allele of 658 bp (c), and an unknown amplicon of ~780 bp (b) (Figure 1E, Figure I_C in the Data Supplement). This PCR gained in *Prdm16^wt/wt^* mice only one product with 807 bp. To identify the molecular nature of the unexpected ~780 bp amplicon we performed a nested PCR (exon_6-8) from agarose gel eluted bands. The nested PCR gained three bands including the control allele of ~300 bp (a), the known exon 7 skipping mutant allele of ~150 bp (c) and an unknown amplicon of ~250 bp (Figure I_D in the Data Supplement). In order to elucidate the nature of the unknown amplicon, probably comprising so far unrecognized transcripts due to altered differential splicing, we performed targeted high throughput sequencing. A histogram of raw sequencing reads demonstrates major peaks at about 150 bp and 300 bp representing the known exon 7 skipping mutant and the control allele, respectively (Figure I_E in the Data Supplement). At ~250 bp a further accumulation of sequencing reads was detected.

In depth analysis of all high throughput sequencing reads identified four differential splice transcripts due to the *Prdm16* acceptor splice site variant c.888-3C>A: Prdm16_mut1 (complete exon 7 skip mutant allele), Prdm16_mut2 (exon 7 skip using an earlier donor splice site), Prdm16_mut3 (using an exon6 donor site and a later exon7 acceptor site), Prdm16_mut4 (using an exon 6 donor and acceptor splice site) (Figure 1F, Figure II in the Data Supplement). The most abundant transcripts Prdm16_mut1 and Prdm16_mut2 result in premature stop of Prdm16 translation. In contrast, Prdm16_mut3 and Prdm16_mut4 result in proteins of 1256 amino acid length lacking a region of 19 amino acids. Overall this suggests that the *Prdm16* acceptor splice site variant c.888-3C>A affects mRNA splicing, produces several splice products, and the two most abundant splice products truncate Prdm16 proteins after approx. 340 amino acids.

*Normal cardiac sarcomere organization in Prdm16^csp1/wt^* *mice*

The impact of Prdm16 on the sarcomere organization is unclear. Cardiac tissue organization assessed with histology, hematoxylin/eosin staining appeared normal in *Prdm16^csp1/wt^* hearts (Figure 2E). Analysis of fibrosis, which may result from myocardial dysfunction, with Picro-Sirius Red staining and immunostaining of collagen 1 (Col1) as well as alpha smooth muscle actin (αSma) did not reveal fibrosis (Figure 2F, Figure III in the Data Supplement). Cardiomyocyte area was assessed with histomorphometry on paraffin embedded heart tissue sections of comparable cross-sectional level that were visualized with wheat germ agglutinin (WGA). Cardiomyocytes of female *Prdm16^csp1/wt^* mice demonstrate significant reduction of cross-sectional area explaining heart hypoplasia (Figure 2G). Electron microscopy did not reveal obvious structural damage of cardiomyocytes, sarcomere, or mitochondria (Figure IV_A in the Data Supplement). Histomorphometry confirmed normal relative sarcomere area, relative mitochondrial area, relative mitochondrial number, and individual mitochondrial area (Figure IV_B-E in the Data Supplement). Also, mitochondria substructure appeared regular and inconspicuous (Figure IV_F in the Data Supplement). Next, transcript levels of sarcomere marker genes were assessed using quantitative PCR (qPCR). Most genes were not differentially expressed but titin-cap (*Tcap*), regulating titin organization in the sarcomere, showed in *Prdm16^csp1/wt^* mice moderate up-regulation (Figure IV_G in the Data Supplement). Sarcomere organization was further assessed with confocal microscopy of tissue sections stained with cardiac troponin T2 (Tnnt2). *Prdm16^csp1/wt^* cardiac tissue revealed regular sarcomeric staining pattern (Figure IV_H in the Data Supplement). Overall, cardiac tissue of *Prdm16^csp1/wt^* mice shows no structural defects on tissue level, mitochondria ultrastructure, and sarcomere organization suggesting normal tissue structure and lack of progressed pathogenic processes.

**Figures Data Supplement**

**Online Figure I**


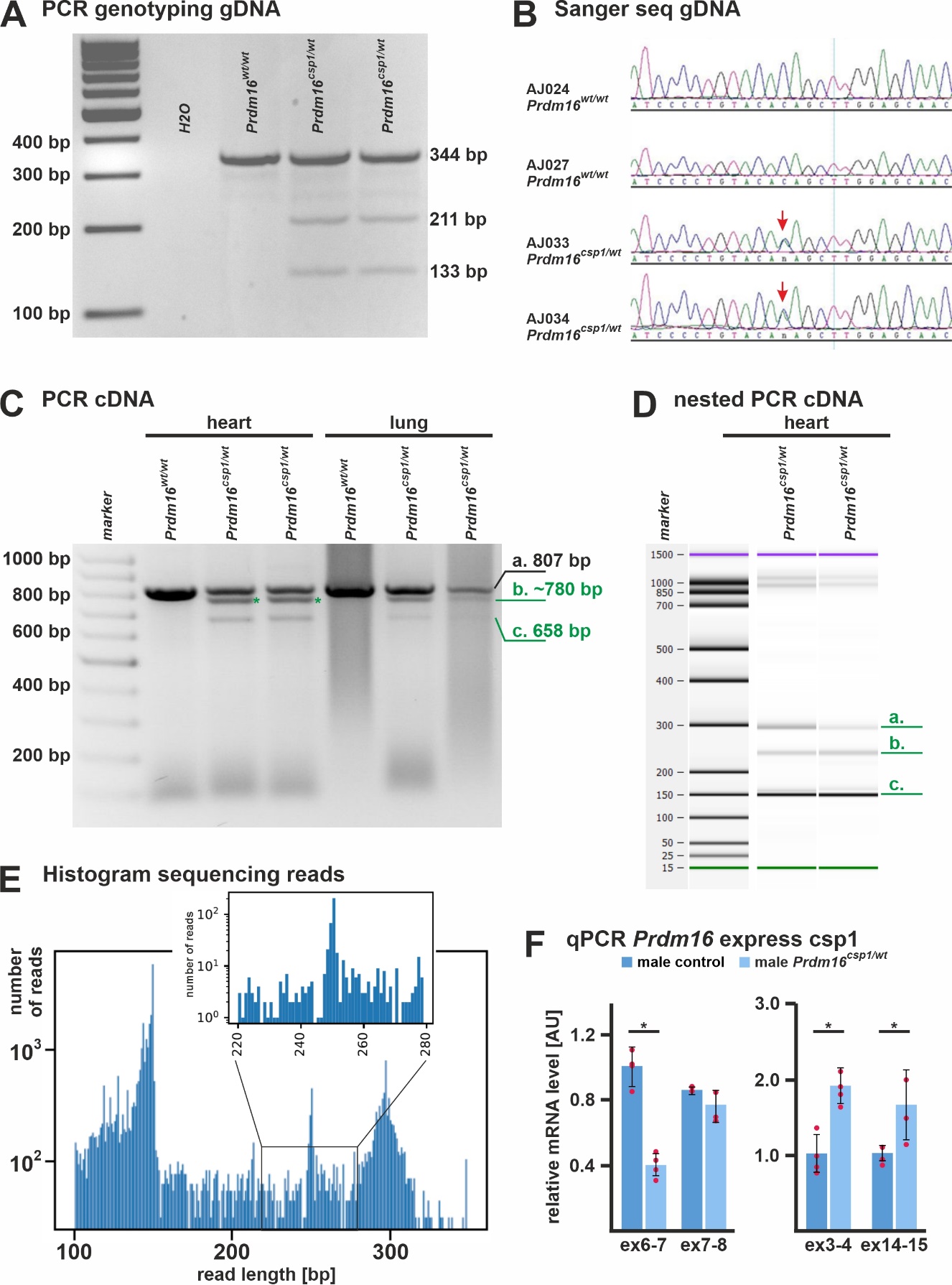


**Online Figure I. Genotyping of *Prdm16^csp1/wt^* mice. (A)** Genotyping occurred from genomic DNA with an endpoint PCR and subsequent *HindIII* digest. Heterozygous *Prdm16^csp1/wt^* mice show three bands with 133 bp, 211 bp, and 344 bp. Control *Prdm16^wt/wt^* mice show one band with 344 bp. **(B)** Sanger sequencing confirmed the presence of the heterozygous genomic mutation c.888-3C>A (red arrows) (ENSMUSG00000039410, ENSMUST00000030902.12). **(C)** Endpoint PCR of complementary DNA (cDNA), generated from lung and heart RNA, revealed three main splice forms in *Prdm16^csp1/wt^* mice with 658 bp (a), ~780 bp (b, green stars), and 807 bp (c). The unexpected amplicon b was gel eluted and further investigated. **(D)** Gel eluted PCR product b (~780 bp) underwent a nested PCR to amplify a region including exon 6-8 (see Table I). The generated PCR product underwent Bioanalyzer analysis and gained three main bands at 150 bp (c), ~250 bp (b), and 300 bp (a). **(E)** Targeted amplicon high-throughput sequencing generated product peaks at ~150 bp, ~250 bp, and ~300 bp. **(F)** Quantitative analysis on male *Prdm16^csp1/wt^* heart tissue detected diminished *Prdm16* transcript levels with detectors targeting the exon 7 deletion region. PCR detectors targeting exon3-4 and exon14-15 identifies increased *Prdm16* levels. Statistical analysis was performed with unpaired t-test, * indicates p<0.05.

**Online Figure II**

**Prdm16_wt transcript, control**

WT / Exon6-Exon7-Exon8 (Ensemble Prdm16-201 ENSMUST00000030902.12)

AGGACCCCACATTCCGCTGTGATGAGTGTGATGAGCTCTTCCAGTGCAGGCTGGACCTGA

GGCGCCACAAGAAGTACGCGTGCAG[C](http://www.ensembl.org/Mus_musculus/ZMenu/TextSequence?db=core;factorytype=Location;g=ENSMUSG00000039410;r=4:154316125-154636873;t=ENSMUST00000030902;v=rs216896689;vf=20397323)TCTGCAGGAGCCCAGCTCTACGAGGGCCTAGGGG

AGGAACTCAAGCCCGAGGGCCTTGGCGTGGGCAGCGACGGGCAAGCGCATGAGTGCAAGG

A[T](http://www.ensembl.org/Mus_musculus/ZMenu/TextSequence?db=core;factorytype=Location;g=ENSMUSG00000039410;r=4:154316125-154636873;t=ENSMUST00000030902;v=rs253441656;vf=56863680)TG[C](http://www.ensembl.org/Mus_musculus/ZMenu/TextSequence?db=core;factorytype=Location;g=ENSMUSG00000039410;r=4:154316125-154636873;t=ENSMUST00000030902;v=rs33056468;vf=5667955)GAGCGGATGTTCCCCAACAAGTACAGCTTGGAGCAACACATGATCGTCCACACGG

AAGAGCGTGAGTACAAATGTGACCAGTGTCCCAAGGCCTTCAACTGGAAGTCCAACCTCA

TCCGCCACCAGATGTCTCACGACAGTGGCAAGCGCTTCGAATGTGAAAACTGTGTCAAGG

TGTTCACGGACCCCAGCAACCTCCAGCGTCACATCCGCTCACAGCATGTCGGTGCCCGGG

CCCATGCCTGCCCTGACTGTGGCAAGACCTTCGCCACATCCTCTGGCCTCAAACAGCACA

AGCATATCCACAGCACGGTGAAGCCATTCATAT

The primer for amplification are underlined.

size PCR product after splicing – 297 bp

Genomic positions for exon-exon junctions

Exon6: 154,348,134-154,347,924

Exon7: 154,346,142-154,345,995

Exon8: 154,345,475-154,345,322

**Amino acid sequence**

EDPTFRCDECDELFQCRLDLRRHKKYACSSAGAQLYEGLGEELKPEGLGVGSDGQAHECK

DCERMFPNKYSLEQHMIVHTEEREYKCDQCPKAFNWKSNLIRHQMSHDSGKRFECENCVK

VFTDPSNLQRHIRSQHVGARAHACPDCGKTFATSSGLKQHKHIHSTVKPFIC

Red amino acids indicate triplets overlapping exon-exon junctions.

**Prdm16 *mus musculus* control**

MRSKARARKLAKSDGDVVNNMYEPDPDLLAGQSAEEETEDGILSPIPMGPPSPFPTSEDF

TPKEGSPYEAPVYIPEDIPIPPDFELRESSIPGAGLGIWAKRKMEIGERFGPYVVTPRAA

LKEADFGWEMLTDTEVSSQESCIKKQISEDLGSEKFCVDANQAGSGSWLKYIRVACSCDD

QNLAMCQINEQIYYKVIKDIEPGEELLVHVKEGAYSLGVMAPSLDEDPTFRCDECDELFQ

CRLDLRRHKKYACSSAGAQLYEGLGEELKPEGLGVGSDGQAHECKDCERMFPNKYSLEQH

MIVHTEEREYKCDQCPKAFNWKSNLIRHQMSHDSGKRFECENCVKVFTDPSNLQRHIRSQ

HVGARAHACPDCGKTFATSSGLKQHKHIHSTVKPFICEVCHKSYTQFSNLCRHKRMHADC

RTQIKCKDCGQMFSTTSSLNKHRRFCEGKNHYTPGSIFTPGLPLTPSPMMDKTKPSPTLN

HGGLGFSEYFPSRPHPGSLPFSAAPPAFPALTPGFPGIFPPSLYPRPPLLPPTPLLKSPL

NHAQDAKLPSPLGNPALPLVSAVSNSSQGATAATGSEEKFDGRLEDAYAEKVKNRSPDMS

DGSDFEDINTTTGTDLDTTTGTGSDLDSDLDSDRDKGKDKGKPVESKPEFGGASVPPGAM

NSVAEVPAFYSQHSFFPPPEEQLLTASGAAGDSIKAIASIAEKYFGPGFMSMQEKKLGSL

PYHSVFPFQFLPNFPHSLYPFTDRALAHNLLVKAEPKSPRDALKVGGPSAECPFDLTTKP

KEAKPALLAPKVPLIPSSGEEQPLDLSIGSRARASQNGGGREPRKNHVYGERKPGVSEGL

PKVCPAQLPQQPSLHYAKPSPFFMDPIYRVEKRKVADPVGVLKEKYLRPSPLLFHPQMSA

IETMTEKLESFAAMKADSGSSLQPLPHHPFNFRSPPPTLSDPILRKGKERYTCRYCGKIF

PRSANLTRHLRTHTGEQPYRCKYCDRSFSISSNLQRHVRNIHNKEKPFKCHLCNRCFGQQ

TNLDRHLKKHEHEGAPVSQHSGVLTNHLGTSASSPTSESDNHALLDEKEDSYFSEIRNFI

ANSEMNQASTRMDKRPEIQDLDSNPPCPGSASAKPEDVEEEEEEELEEEDDDSLAGKSQE

DTVSPTPEPQGVYEDEEDEEPPSLTMGFDHTRRCVEERGGGLLALEPTPTFGKGLDLRRA

AEEAFEVKDVLNSTLDSEVLKQTLYRQAKNQAYAMMLSLSEDTPLHAPSQSSLDAWLNIT

GPSSESGAFNPINHL*

**Prdm16_mut1 transcript, exon7 skip splicing**

mut1 / Exon6--Exon8 (Ensemble Prdm16-201 ENSMUST00000030902.12)

AGGACCCCACATTCCGCTGTGATGAGTGTGATGAGCTCTTCCAGTGCAGGCTGGACCTGA

GGCGCCACAAGAAGTACGCGTGCAG[C](http://www.ensembl.org/Mus_musculus/ZMenu/TextSequence?db=core;factorytype=Location;g=ENSMUSG00000039410;r=4:154316125-154636873;t=ENSMUST00000030902;v=rs216896689;vf=20397323)TCTGCAGGAGCCCAGCTCTACGAGGGCCTAGGGG

AGGAACTCAAGCCCGAGGGCCTTGGCGTGGGCAGCGACGGGCAAGCGCATGAGTGCAAGG

A[T](http://www.ensembl.org/Mus_musculus/ZMenu/TextSequence?db=core;factorytype=Location;g=ENSMUSG00000039410;r=4:154316125-154636873;t=ENSMUST00000030902;v=rs253441656;vf=56863680)TG[C](http://www.ensembl.org/Mus_musculus/ZMenu/TextSequence?db=core;factorytype=Location;g=ENSMUSG00000039410;r=4:154316125-154636873;t=ENSMUST00000030902;v=rs33056468;vf=5667955)GAGCGGATGTTCCCCAACAAGTACAGGTGTTCACGGACCCCAGCAACCTCCAGCG

TCACATCCGCTCACAGCATGTCGGTGCCCGGGCCCATGCCTGCCCTGACTGTGGCAAGAC

CTTCGCCACATCCTCTGGCCTCAAACAGCACAAGCATATCCACAGCACGGTGAAGCCATT

CATAT

The primer for amplification are underlined.

size PCR product after splicing – 149 bp

Genomic positions for exon-exon junctions

Exon6: 154,348,134-154,347,924

Exon7: -

Exon8: 154,345,475-154,345,322

**Amino acid sequence**

EDPTFRCDECDELFQCRLDLRRHKKYACSSAGAQLYEGLGEELKPEGLGVGSDGQAHECK

DCERMFPNKYRCSRTPATSSVTSAHSMSVPGPMPALTVARPSPHPLASNSTSISTAR*

Red amino acids indicate triplets overlapping exon-exon junctions.

Magenta amino acids indicate novel peptide sequence and stop codon.

**Complete Prdm16 *mus musculus* Exon7 skip splicing**

MRSKARARKLAKSDGDVVNNMYEPDPDLLAGQSAEEETEDGILSPIPMGPPSPFPTSEDF

TPKEGSPYEAPVYIPEDIPIPPDFELRESSIPGAGLGIWAKRKMEIGERFGPYVVTPRAA

LKEADFGWEMLTDTEVSSQESCIKKQISEDLGSEKFCVDANQAGSGSWLKYIRVACSCDD

QNLAMCQINEQIYYKVIKDIEPGEELLVHVKEGAYSLGVMAPSLDEDPTFRCDECDELFQ

CRLDLRRHKKYACSSAGAQLYEGLGEELKPEGLGVGSDGQAHECKDCERMFPNKYRCSRT

PATSSVTSAHSMSVPGPMPALTVARPSPHPLASNSTSISTAR*

**Prdm16_mut2 transcript, exon7 skip earlier donor site splicing**

mut2 / Exon6--Exon8 (Ensemble Prdm16-201 ENSMUST00000030902.12)

AGGACCCCACATTCCGCTGTGATGAGTGTGATGAGCTCTTCCAGTGCAGGCTGGACCTGA

GGCGCCACAAGAAGTACGCGTGCAG[C](http://www.ensembl.org/Mus_musculus/ZMenu/TextSequence?db=core;factorytype=Location;g=ENSMUSG00000039410;r=4:154316125-154636873;t=ENSMUST00000030902;v=rs216896689;vf=20397323)TCTGCAGGAGCCCAGCTCTACGAGGGCCTAGGGG

AGGAACTCAAGCCCGAGGGCCTTGGCGTGGGCAGCGACGGGCAAGCGCATGAGTGCAAGG

A[T](http://www.ensembl.org/Mus_musculus/ZMenu/TextSequence?db=core;factorytype=Location;g=ENSMUSG00000039410;r=4:154316125-154636873;t=ENSMUST00000030902;v=rs253441656;vf=56863680)TG[C](http://www.ensembl.org/Mus_musculus/ZMenu/TextSequence?db=core;factorytype=Location;g=ENSMUSG00000039410;r=4:154316125-154636873;t=ENSMUST00000030902;v=rs33056468;vf=5667955)GAGCGGATGTTCCCCAACAAGTGTTCACGGACCCCAGCAACCTCCAGCGTCACAT

CCGCTCACAGCATGTCGGTGCCCGGGCCCATGCCTGCCCTGACTGTGGCAAGACCTTCGC

CACATCCTCTGGCCTCAAACAGCACAAGCATATCCACAGCACGGTGAAGCCATTCATAT

The primer for amplification are underlined.

size PCR product after splicing: 143 bp

Exon6: 154,348,134-154,347,930

Exon7: -

Exon8: 154,345,475-154,345,322

**Amino acid sequence**

EDPTFRCDECDELFQCRLDLRRHKKYACSSAGAQLYEGLGEELKPEGLGVGSDGQAHECK

DCERMFPNKCSRTPATSSVTSAHSMSVPGPMPALTVARPSPHPLASNSTSISTAR*

Red amino acids indicate triplets overlapping exon-exon junctions.

Magenta amino acids indicate novel peptide sequence and stop codon.

**Complete Prdm16 *mus musculus* Exon7 skip earlier donor site splicing**

MRSKARARKLAKSDGDVVNNMYEPDPDLLAGQSAEEETEDGILSPIPMGPPSPFPTSEDF

TPKEGSPYEAPVYIPEDIPIPPDFELRESSIPGAGLGIWAKRKMEIGERFGPYVVTPRAA

LKEADFGWEMLTDTEVSSQESCIKKQISEDLGSEKFCVDANQAGSGSWLKYIRVACSCDD

QNLAMCQINEQIYYKVIKDIEPGEELLVHVKEGAYSLGVMAPSLDEDPTFRCDECDELFQ

CRLDLRRHKKYACSSAGAQLYEGLGEELKPEGLGVGSDGQAHECKDCERMFPNKCSRTPA

TSSVTSAHSMSVPGPMPALTVARPSPHPLASNSTSISTAR*

**Prdm16_mut3 transcript, exon6 earlier donor site & exon7 later acceptor site splicing**

Frequent transcript in range 235-255 nt

mut3 / Exon6partially-Exon7partially-Exon8 (Ensemble Prdm16-201 ENSMUST00000030902.12)

AGGACCCCACATTCCGCTGTGATGAGTGTGATGAGCTCTTCCAGTGCAGGCTGGACCTGA

GGCGCCACAAGAAGTACGCGTGCAG[C](http://www.ensembl.org/Mus_musculus/ZMenu/TextSequence?db=core;factorytype=Location;g=ENSMUSG00000039410;r=4:154316125-154636873;t=ENSMUST00000030902;v=rs216896689;vf=20397323)TCTGCAGGAGCCCAGCTCTACGAGGGCCTAGGGG

AGGAACTCAAGCCCGAGGGCCTTGGCGTGGGCAGCGACGAGCAACACATGATCGTCCACA

CGGAAGAGCGTGAGTACAAATGTGACCAGTGTCCCAAGGCCTTCAACTGGAAGTCCAACC

TCATCCGCCACCAGATGTCTCACGACAGTGGCAAGCGCTTCGAATGTGAAAACTGTGTCA

AGGTGTTCACGGACCCCAGCAACCTCCAGCGTCACATCCGCTCACAGCATGTCGGTGCCC

GGGCCCATGCCTGCCCTGACTGTGGCAAGACCTTCGCCACATCCTCTGGCCTCAAACAGC

ACAAGCATATCCACAGCACGGTGAAGCCATTCATAT

The primer for amplification are underlined.

size PCR product after splicing: 240 bp

Exon6: 154,348,134-154,347,976

Exon7: 154,346,137-154,345,995

Exon8: 154,345,475-154,345,322

**Amino acid sequence**

EDPTFRCDECDELFQCRLDLRRHKKYACSSAGAQLYEGLGEELKPEGLGVGSDEQHMIVH

TEEREYKCDQCPKAFNWKSNLIRHQMSHDSGKRFECENCVKVFTDPSNLQRHIRSQHVGA

RAHACPDCGKTFATSSGLKQHKHIHSTVKPFI - inframe deletion, no truncation

Red amino acids indicate triplets overlapping exon-exon junctions.

Magenta amino acids indicate novel peptide sequence. A Prdm16 protein with a 19aa deletion and total size of 1256 aa is generated.

**Complete Prdm16 *mus musculus* Exon6 earlier donor site & Exon7**

**later acceptor site splicing**

MRSKARARKLAKSDGDVVNNMYEPDPDLLAGQSAEEETEDGILSPIPMGPPSPFPTSEDF

TPKEGSPYEAPVYIPEDIPIPPDFELRESSIPGAGLGIWAKRKMEIGERFGPYVVTPRAA

LKEADFGWEMLTDTEVSSQESCIKKQISEDLGSEKFCVDANQAGSGSWLKYIRVACSCDD

QNLAMCQINEQIYYKVIKDIEPGEELLVHVKEGAYSLGVMAPSLDEDPTFRCDECDELFQ

CRLDLRRHKKYACSSAGAQLYEGLGEELKPEGLGVGSDEQHMIVHTEEREYKCDQCPKAF

NWKSNLIRHQMSHDSGKRFECENCVKVFTDPSNLQRHIRSQHVGARAHACPDCGKTFATS

SGLKQHKHIHSTVKPFICEVCHKSYTQFSNLCRHKRMHADCRTQIKCKDCGQMFSTTSSL

NKHRRFCEGKNHYTPGSIFTPGLPLTPSPMMDKTKPSPTLNHGGLGFSEYFPSRPHPGSL

PFSAAPPAFPALTPGFPGIFPPSLYPRPPLLPPTPLLKSPLNHAQDAKLPSPLGNPALPL

VSAVSNSSQGATAATGSEEKFDGRLEDAYAEKVKNRSPDMSDGSDFEDINTTTGTDLDTT

TGTGSDLDSDLDSDRDKGKDKGKPVESKPEFGGASVPPGAMNSVAEVPAFYSQHSFFPPP

EEQLLTASGAAGDSIKAIASIAEKYFGPGFMSMQEKKLGSLPYHSVFPFQFLPNFPHSLY

PFTDRALAHNLLVKAEPKSPRDALKVGGPSAECPFDLTTKPKEAKPALLAPKVPLIPSSG

EEQPLDLSIGSRARASQNGGGREPRKNHVYGERKPGVSEGLPKVCPAQLPQQPSLHYAKP

SPFFMDPIYRVEKRKVADPVGVLKEKYLRPSPLLFHPQMSAIETMTEKLESFAAMKADSG

SSLQPLPHHPFNFRSPPPTLSDPILRKGKERYTCRYCGKIFPRSANLTRHLRTHTGEQPY

RCKYCDRSFSISSNLQRHVRNIHNKEKPFKCHLCNRCFGQQTNLDRHLKKHEHEGAPVSQ

HSGVLTNHLGTSASSPTSESDNHALLDEKEDSYFSEIRNFIANSEMNQASTRMDKRPEIQ

DLDSNPPCPGSASAKPEDVEEEEEEELEEEDDDSLAGKSQEDTVSPTPEPQGVYEDEEDE

EPPSLTMGFDHTRRCVEERGGGLLALEPTPTFGKGLDLRRAAEEAFEVKDVLNSTLDSEV

LKQTLYRQAKNQAYAMMLSLSEDTPLHAPSQSSLDAWLNITGPSSESGAFNPINHL*

**Prdm16_mut4 transcript, exon6 donor & exon6 acceptor site splicing**

Frequent transcript in range 235-255 nt

mut4 / Exon6a-Exon6b-Exon7-Exon8

(Ensemble Prdm16-201 ENSMUST00000030902.12)

AGGACCCCACATTCCGCTGTGATGAGTGTGATGAGCTCTTCCAGTGCAGGCTGGACCTGA

GGCGCCACAAGAAGTACGCGTGCAG[C](http://www.ensembl.org/Mus_musculus/ZMenu/TextSequence?db=core;factorytype=Location;g=ENSMUSG00000039410;r=4:154316125-154636873;t=ENSMUST00000030902;v=rs216896689;vf=20397323)TCTGCAGGAGCCCAGCTCTACGAGGGCCTAGGGG

AGGA[T](http://www.ensembl.org/Mus_musculus/ZMenu/TextSequence?db=core;factorytype=Location;g=ENSMUSG00000039410;r=4:154316125-154636873;t=ENSMUST00000030902;v=rs253441656;vf=56863680)TG[C](http://www.ensembl.org/Mus_musculus/ZMenu/TextSequence?db=core;factorytype=Location;g=ENSMUSG00000039410;r=4:154316125-154636873;t=ENSMUST00000030902;v=rs33056468;vf=5667955)GAGCGGATGTTCCCCAACAAGTACAGCTTGGAGCAACACATGATCGTCCACA

CGGAAGAGCGTGAGTACAAATGTGACCAGTGTCCCAAGGCCTTCAACTGGAAGTCCAACC

TCATCCGCCACCAGATGTCTCACGACAGTGGCAAGCGCTTCGAATGTGAAAACTGTGTCA

AGGTGTTCACGGACCCCAGCAACCTCCAGCGTCACATCCGCTCACAGCATGTCGGTGCCC

GGGCCCATGCCTGCCCTGACTGTGGCAAGACCTTCGCCACATCCTCTGGCCTCAAACAGC

ACAAGCATATCCACAGCACGGTGAAGCCATTCATAT

The primer for amplification are underlined.

size PCR product after splicing: 240 bp

Exon6: 154,348,134-154.348.011;154.347.953-154.347.924

Exon7: 154.346.142-154,345,995

Exon8: 154,345,475-154,345,322

**Amino acid sequence**

EDPTFRCDECDELFQCRLDLRRHKKYACSSAGAQLYEGLGEDCERMFPNKYSLEQHMIVH

TEEREYKCDQCPKAFNWKSNLIRHQMSHDSGKRFECENCVKVFTDPSNLQRHIRSQHVGA

RAHACPDCGKTFATSSGLKQHKHIHSTVKPFI - inframe deletion, no truncation

Red amino acids indicate triplets overlapping exon-exon junctions.

Magenta amino acids indicate novel peptide sequence. A PRDM16 protein with a 19aa deletion and total size of 1256 aa is generated.

**Complete Prdm16 *mus musculus* Exon6 donor & acceptor site**

MRSKARARKLAKSDGDVVNNMYEPDPDLLAGQSAEEETEDGILSPIPMGPPSPFPTSEDF

TPKEGSPYEAPVYIPEDIPIPPDFELRESSIPGAGLGIWAKRKMEIGERFGPYVVTPRAA

LKEADFGWEMLTDTEVSSQESCIKKQISEDLGSEKFCVDANQAGSGSWLKYIRVACSCDD

QNLAMCQINEQIYYKVIKDIEPGEELLVHVKEGAYSLGVMAPSLDEDPTFRCDECDELFQ

CRLDLRRHKKYACSSAGAQLYEGLGEDCERMFPNKYSLEQHMIVHTEEREYKCDQCPKAF

NWKSNLIRHQMSHDSGKRFECENCVKVFTDPSNLQRHIRSQHVGARAHACPDCGKTFATS

SGLKQHKHIHSTVKPFICEVCHKSYTQFSNLCRHKRMHADCRTQIKCKDCGQMFSTTSSL

NKHRRFCEGKNHYTPGSIFTPGLPLTPSPMMDKTKPSPTLNHGGLGFSEYFPSRPHPGSL

PFSAAPPAFPALTPGFPGIFPPSLYPRPPLLPPTPLLKSPLNHAQDAKLPSPLGNPALPL

VSAVSNSSQGATAATGSEEKFDGRLEDAYAEKVKNRSPDMSDGSDFEDINTTTGTDLDTT

TGTGSDLDSDLDSDRDKGKDKGKPVESKPEFGGASVPPGAMNSVAEVPAFYSQHSFFPPP

EEQLLTASGAAGDSIKAIASIAEKYFGPGFMSMQEKKLGSLPYHSVFPFQFLPNFPHSLY

PFTDRALAHNLLVKAEPKSPRDALKVGGPSAECPFDLTTKPKEAKPALLAPKVPLIPSSG

EEQPLDLSIGSRARASQNGGGREPRKNHVYGERKPGVSEGLPKVCPAQLPQQPSLHYAKP

SPFFMDPIYRVEKRKVADPVGVLKEKYLRPSPLLFHPQMSAIETMTEKLESFAAMKADSG

SSLQPLPHHPFNFRSPPPTLSDPILRKGKERYTCRYCGKIFPRSANLTRHLRTHTGEQPY

RCKYCDRSFSISSNLQRHVRNIHNKEKPFKCHLCNRCFGQQTNLDRHLKKHEHEGAPVSQ

HSGVLTNHLGTSASSPTSESDNHALLDEKEDSYFSEIRNFIANSEMNQASTRMDKRPEIQ

DLDSNPPCPGSASAKPEDVEEEEEEELEEEDDDSLAGKSQEDTVSPTPEPQGVYEDEEDE

EPPSLTMGFDHTRRCVEERGGGLLALEPTPTFGKGLDLRRAAEEAFEVKDVLNSTLDSEV

LKQTLYRQAKNQAYAMMLSLSEDTPLHAPSQSSLDAWLNITGPSSESGAFNPINHL*

**Online Figure II. Splicing products in *Prdm16^csp1/wt^* mice.** Differential splicing in heterozygous *Prdm16^csp1/wt^* mice was assessed with targeted NGS and bioinformatically evaluation revealed several splice products. Most frequently the splice product Prdm16_mut1, generated from exon 7 skipping, was detected. The remaining splice products Prdm16_mut2, Prdm16_mut3, and Prdm16_mut4 were detected with lower abundance.

**Online Figure III**


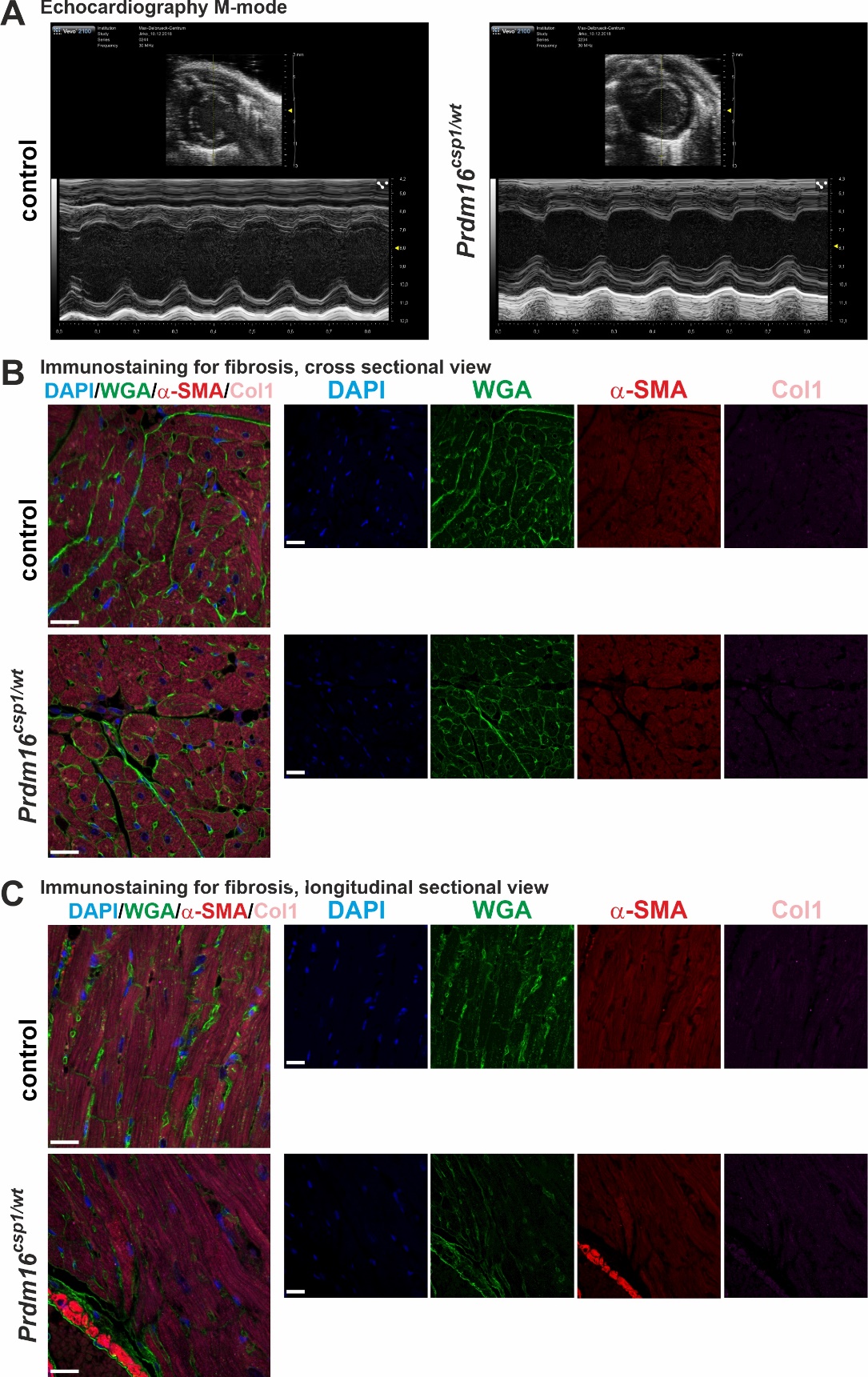


**Online Figure III. Echocardiography and absence of fibrosis in *Prdm16^csp1/wt^* heart tissue. (A)** These images represent the original echocardiography data with time stamp and scale bar shown in Figure 2C. **(B-C)** Presence of cardiac fibrosis was tested on paraformaldehyde fixed, paraffin embedded heart sections with immunostaining of alpha-smooth muscle actin (α-Sma) and collagen (Col1). Subsequently, immunostaining was imaged with confocal microscopy in a multi-channel mode DAPI (blue, nuclei), wheat-germ agglutinin (WGA, green), α-Sma (red), and Col1 (pink). **(B)** Images show the cross-sectional view of cardiomyocytes in heart tissue. **(C)** Images show the longitudinal view of cardiomyocytes in heart tissue. Scale bar is 20 µm.

**Online Figure IV**


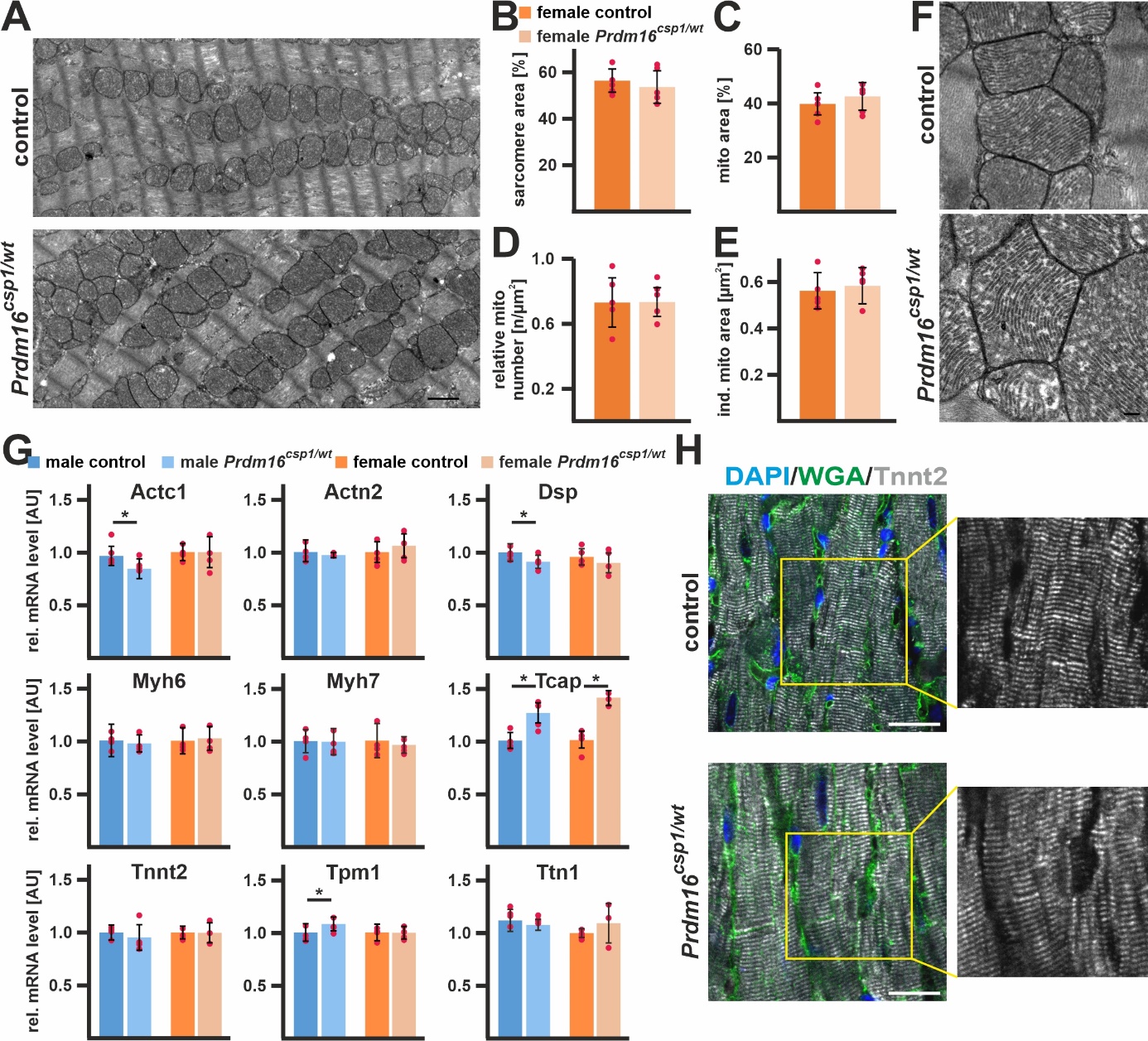


**Online Figure IV. Normal heart tissue organization in *Prdm16^csp1/wt^* mice. (A)** Electron microscopy reveals normal myocardial organization. Scale bar is 1000 nm. Detailed information is provided in the Results of the Data Supplement. **(B-C)** Morphometry of electron microscopy images demonstrate normal relative sarcomere and mitochondrial area in female *Prdm16^csp1/wt^* myocardium compared to controls. **(D-E)** The relative mitochondrial number and individual mitochondrial area are normal in *Prdm16^csp1/wt^* myocardium. **(F)** Mitochondrial ultrastructure is normal in *Prdm16^csp1/wt^* myocardium. Scale bar is 200 nm. **(G)** Expression analysis of sarcomere marker transcripts with qPCR shows unaffected expression. Individual markers such as titin-cap (*Tcap*) show altered expression in males and/or females. **(H)** Normal sarcomere organization was visualized with immunostaining of Troponin T2, cardiac type (Tnnt2, grey). Counterstaining occurred for nuclei (DAPI, blue) and cell membranes (WGA, green). Scale bar is 20 µm. Statistical analysis was performed with unpaired t-test (p<0.05).

**Online Figure V**


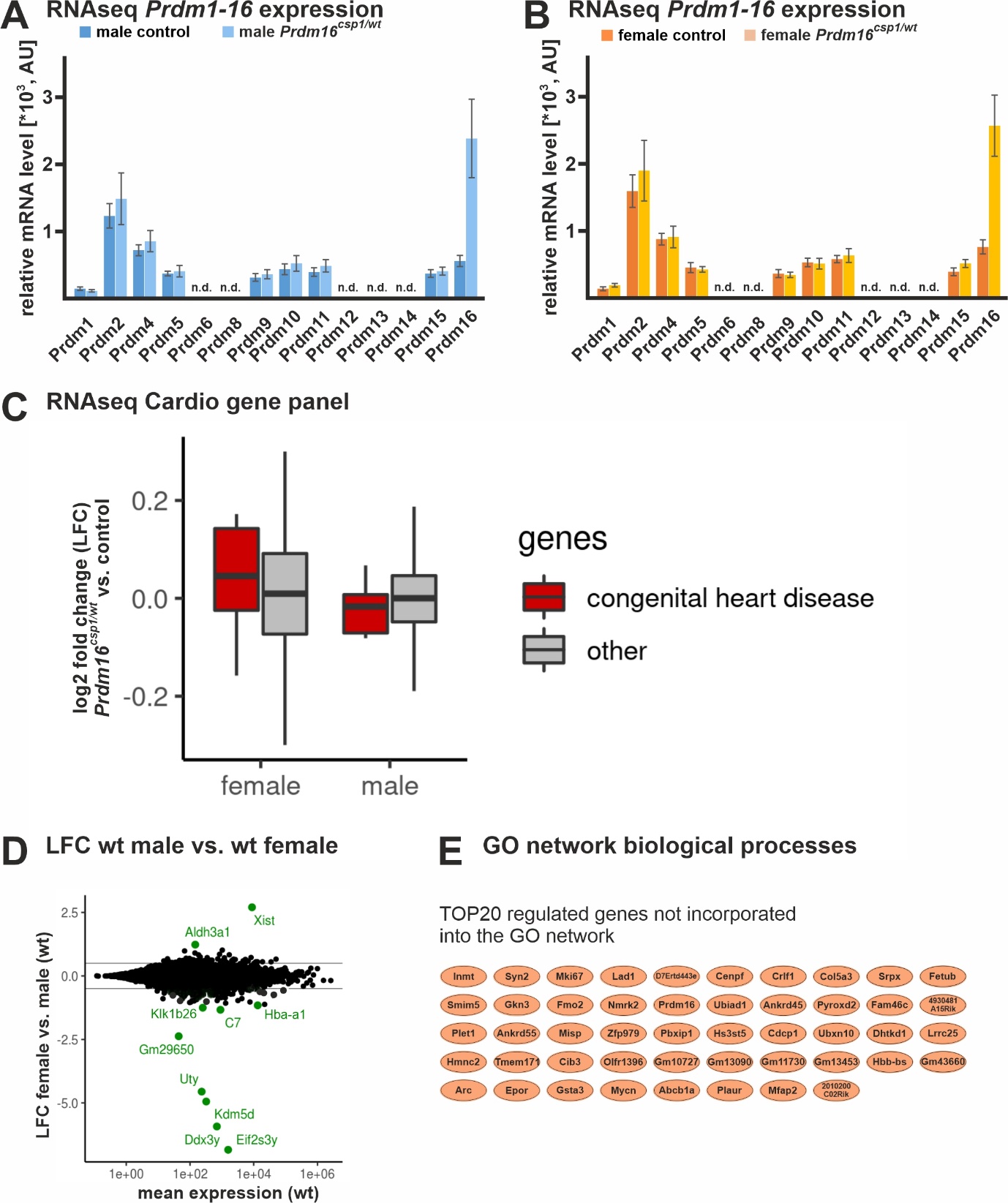


**Online Figure V. Expanded transcriptome, RNAseq analysis. (A)** Expression of different PRDM transcripts was analyzed with RNAseq. Normalized read counts are presented for *Prdm1* to *Prdm16* transcripts from male heart tissue. **(B)** Normalized read counts are presented for *Prdm1* to *Prdm16* transcripts from female heart tissue. **(C)** Expanded analysis of RNAseq data for dysregulation of a cardiac disease gene panel comprising 910 genes relevant for heart disease/function of the harmonizome gene set *congenital heart disease*. Box plot indicates 25th and 75th percentile with a black line at the median, whiskers extend to 1.5 times the interquartile range (IQR). **(D)** Scatterplots show the LFC against mean expression for the sex-specific contrast female (wt) vs. male controls (wt). **(E)** Genes of the TOP20 regulated targets that were not incorporated into the GO network shown in Figure 5F.

**Online Figure VI**

**Figure VI. Regulated proteins between in *Prdm16^csp1/wt^* and control heart tissue.** Heat map illustrating expression of all differentially expressed proteins with a p-value <0.01 of male, female, and male+female *Prdm16^csp1/wt^* /control contrasts. Z-scored values of group median intensity was used. In total 53 differentially expressed proteins were identified.

**Online Figure VII**


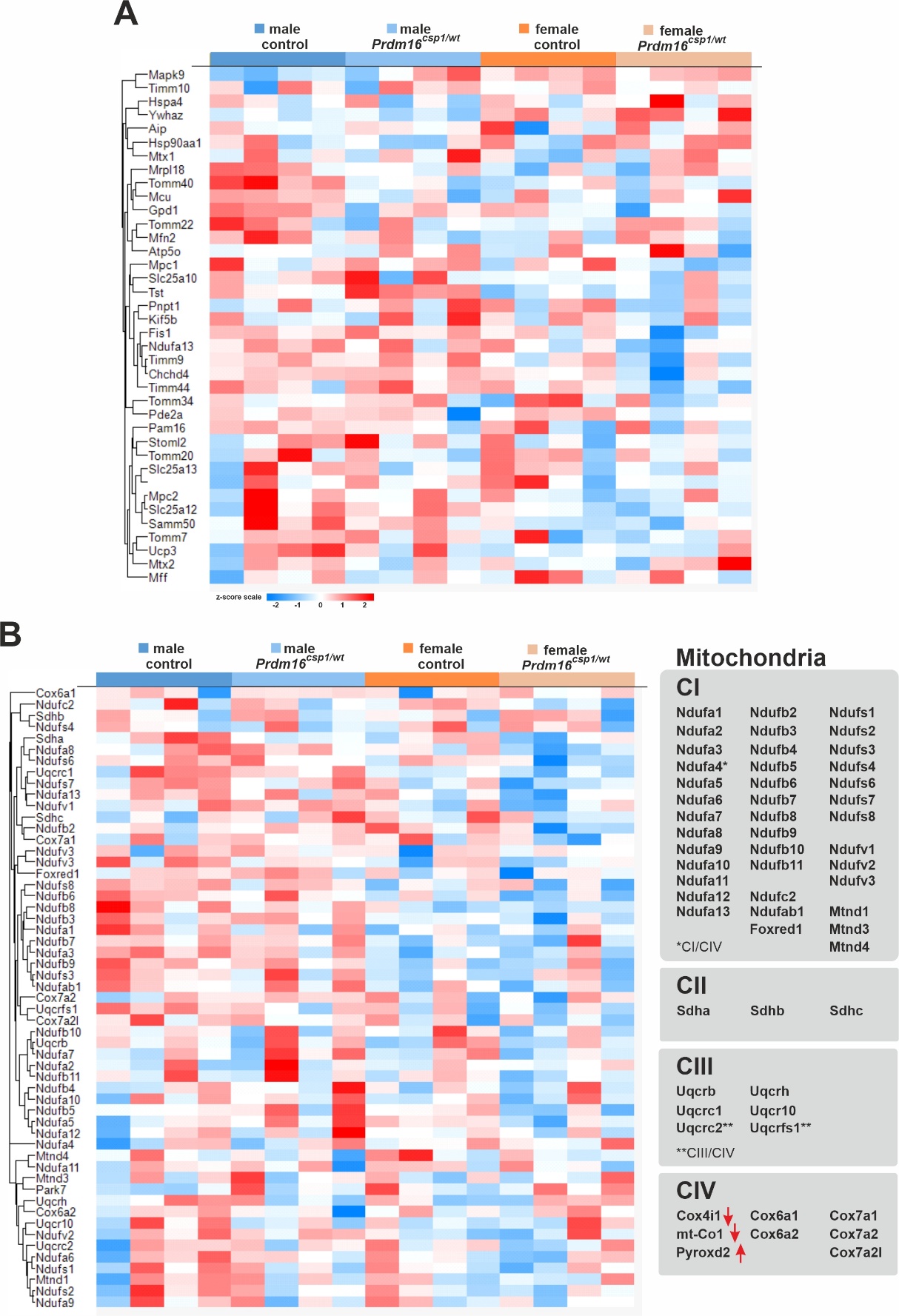


**Figure VII. Proteome profile of mitochondrial proteins in *Prdm16^csp1/wt^* heart tissue.** Heat map of z-scored expression values from **(A)** mitochondrial transport proteins and **(B)** from mitochondrial respiratory chain complexes. **(B)** The detected proteins were ordered to mitochondrial respiratory chain complex I-IV. Pyroxd2 was upregulated in proteome screening. Cox4i1 and mt-Co1 were decreased in *Prdm16^csp1/wt^* hearts using Western blot (see Figure 4).

**Online Figure VIII**


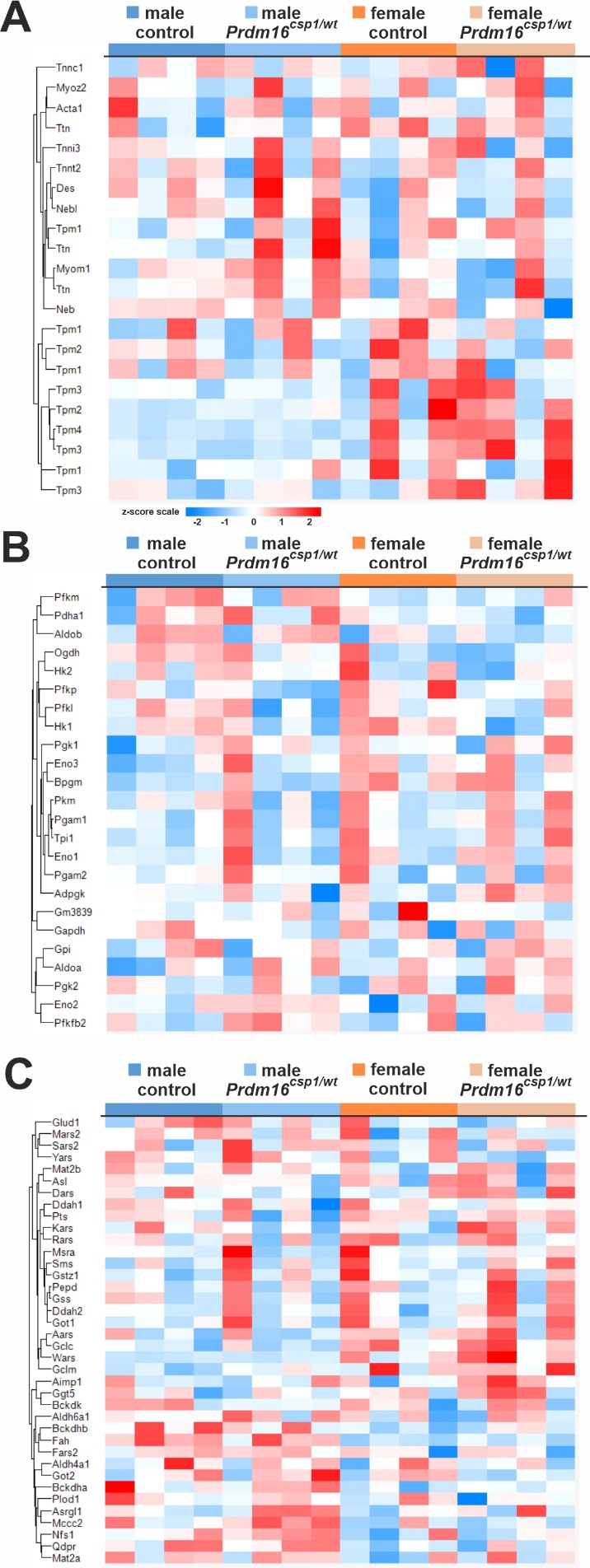


**Figure VIII. Proteome profile of sarcomere and metabolic proteins in *Prdm16^csp1/wt^* heart tissue.** Heat map of z-scored expression values from **(A)** sarcomeric proteins (Tpm1, Tpm2, Tpm3, and Tpm4 proteins originate from independently detected peptides), **(B)** metabolic process glycolysis, and **(C)** amino acid metabolism.

**Online Figure IX**
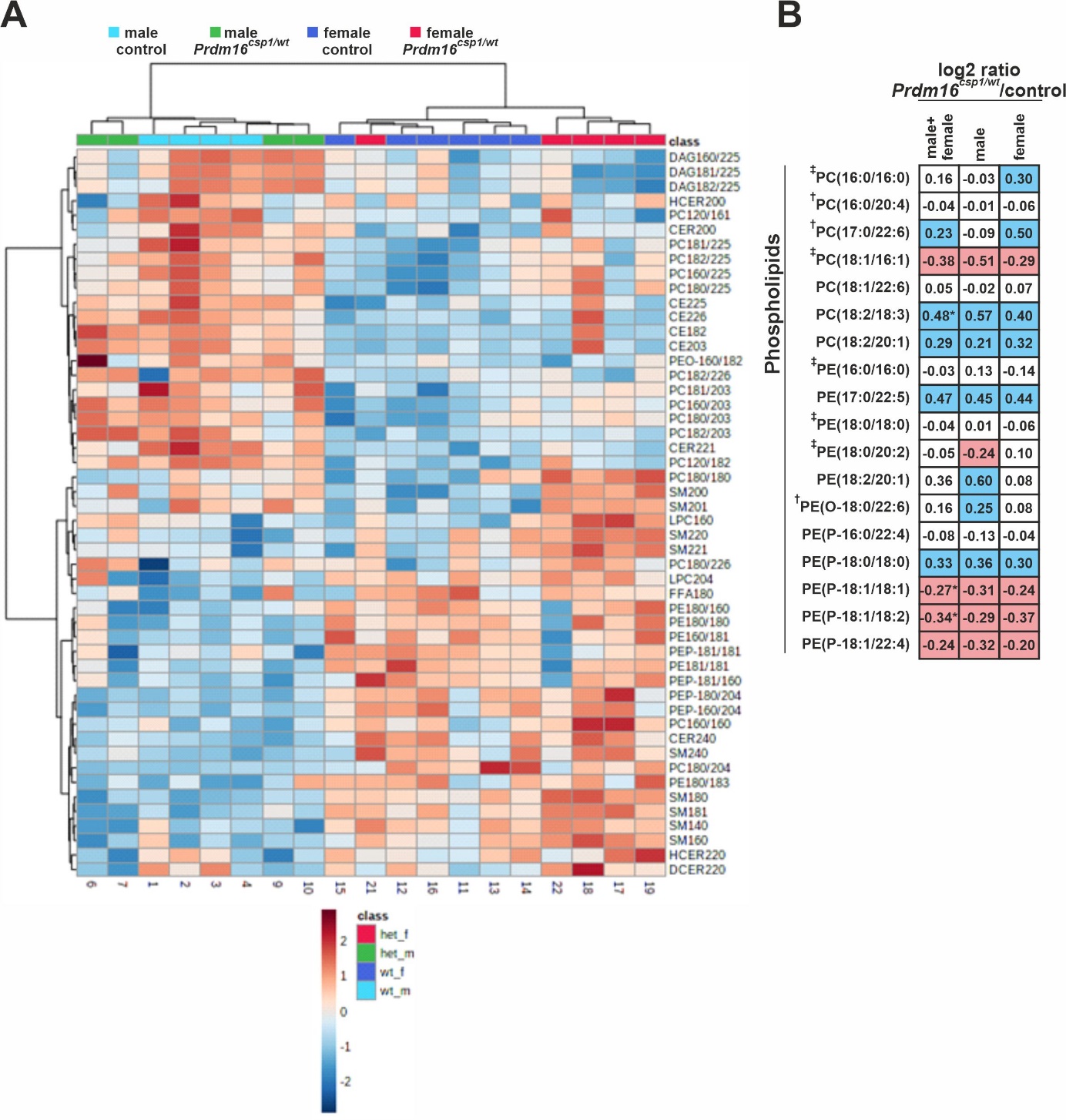


**Figure IX. Lipid profiling of *Prdm16^csp1/wt^* heart tissue. (A)** Lipid analysis was performed with LC-MS using the lipidizer kit (Sciex) and data are shown as log2 value of the intensity ratio *Prdm16^csp1/wt^*/controls. Heat map comprises TOP50 features ranked by t-test in male and female *Prdm16^csp1/wt^* hearts. TAGs were not implemented in the heat map. **(B)** Selected phospholipids critical for the heart, phospholipids altered in *Prdm16^csp1/wt^* mice, and phospholipids previously associated with heart function (^†^*Tham* et al., DOI: [10.1016/j.celrep.2018.08.017](https://doi.org/10.1016/j.celrep.2018.08.017); ^‡^*Wittenbecher* et al., DOI:[10.1161/CIRCRESAHA.120.317883](https://doi.org/10.1161/circresaha.120.317883)) are presented for *Prdm16^csp1/wt^* heart tissue of both sexes and in combination. Statistical analysis of selected lipids was performed with Wilcoxon Sum Rank test, * indicates p<0.05. Coloring indicates reduction (red) or increase (blue) of the log2 ratio by -0.2 or 0.2, respectively.

**Online Figure X**


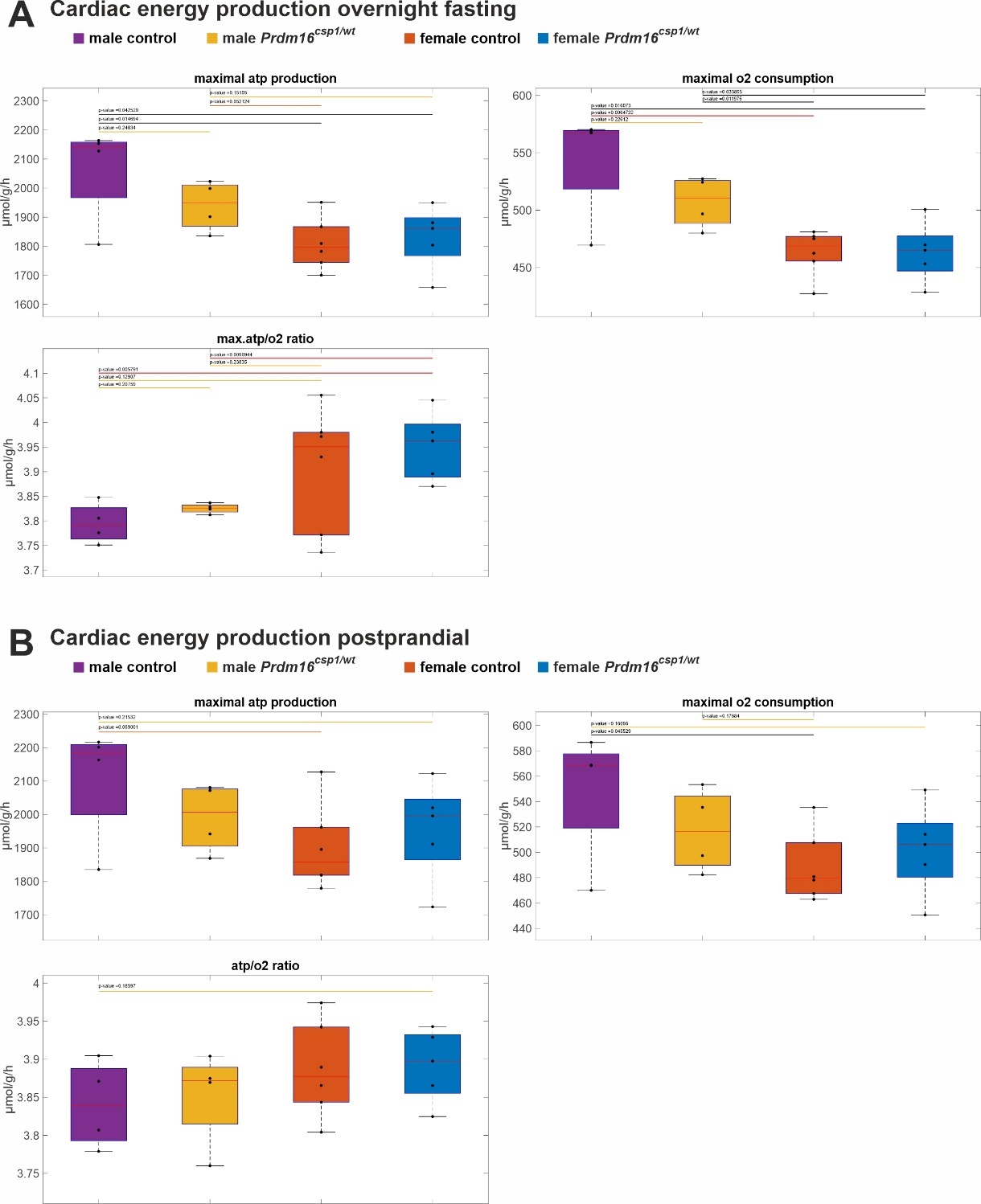


**Figure X. Metabolic modeling of *Prdm16^csp1/wt^* heart tissue using CARDIOKIN1.** Maximal ATP production capacity and associated oxygen consumption rate as well as ATP/O2 ratio for overnight fasted conditions **(A)** and postprandial conditions **(B)**. The ATP consumption rate was modelled by a generic hyperbolic rate law $v_{ATP}=k_{load}\cdot\frac{ATP}{ATP+K_{m}}$. The parameter $k_{load}$ was stepwise increased until the ATP production rate converged to its maximal value. For further details see Berndt et al. 2021. Box plots depict mean values, upper and lower quartiles. Black dots give maximal capacities for individual animals. Significance level between groups, evaluated by two-sided t-test, is indicated by bracket on top.

**Online Figure XI**


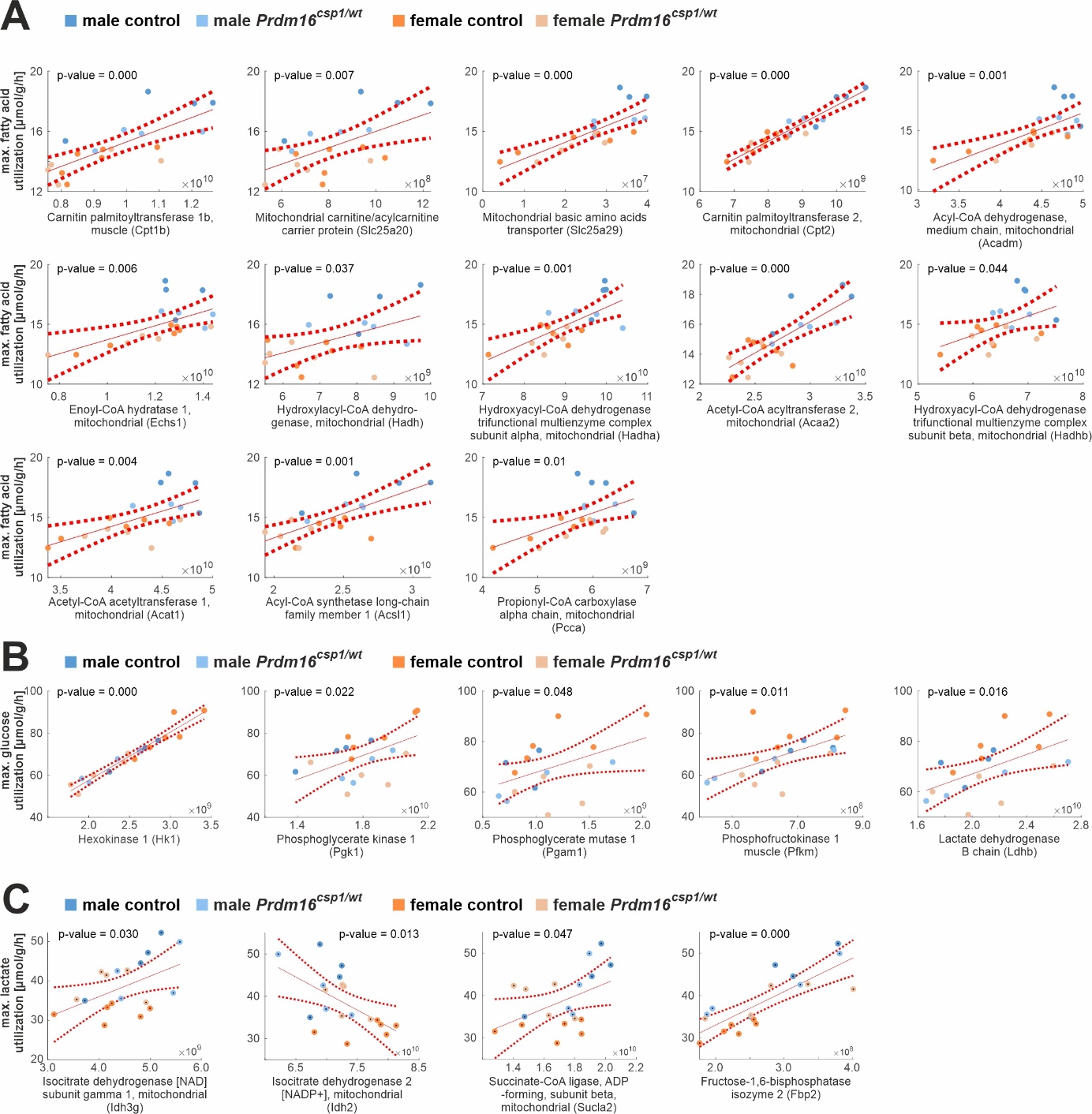


**Figure XI. Correlation analysis of the individual protein impact in *Prdm16^csp1/wt^* heart tissue using CARDIOKIN1.** The impact of individual proteins for **(A)** fatty acid, **(B)** glucose, and **(C)** lactate metabolism was correlated for all analyzed proteins using linear regression analysis of the maximal substrate utilization vs. protein abundance (red dashed line indicates confidence interval, 95%). The following significantly altered proteins are presented: Acetyl-CoA acyltransferase 2, mitochondrial (Acaa2); Acyl-CoA dehydrogenase, medium chain, mitochondrial (Acadm); Acetyl-CoA acetyltransferase 1, mitochondrial (Acat1); Acyl-CoA synthetase long-chain family member 1 (Acsl1); Carnitine palmitoyltransferase 1b, muscle (Cpt1b); Carnitine palmitoyltransferase 2, mitochondrial (Cpt2); Enoyl-CoA hydratase, short chain, 1, mitochondrial (Echs1); Fructose-1,6-bisphosphatase isozyme 2 (Fbp2); Hydroxyacyl-CoA dehydrogenase, mitochondrial (Hadh); Hydroxyacyl-CoA dehydrogenase trifunctional multienzyme complex subunit alpha, mitochondrial (Hadha); Hydroxyacyl-CoA dehydrogenase trifunctional multienzyme complex subunit beta (Hadhb); Hexokinase 1 (Hk1); Isocitrate dehydrogenase 2 (NADP+), mitochondrial (Idh2); Isocitrate dehydrogenase (NAD+), subunit gamma 1, mitochondrial (Idh3g); Lactate dehydrogenase B chain (Ldhb); Propionyl-CoA carboxylase, alpha chain, mitochondrial (Pcca); Phosphofructokinase 1, muscle (Pfkm); Phosphoglycerate mutase 1 (Pgam1); Phosphoglycerate kinase 1 (Pgk1); Mitochondrial carnitine/acylcarnitine carrier protein (Slc25a20); Mitochondrial basic amino acids transporter (Slc25a29); Succinate-CoA ligase, ADP-forming, beta subunit (Sucla2)

**Online Figure XII**


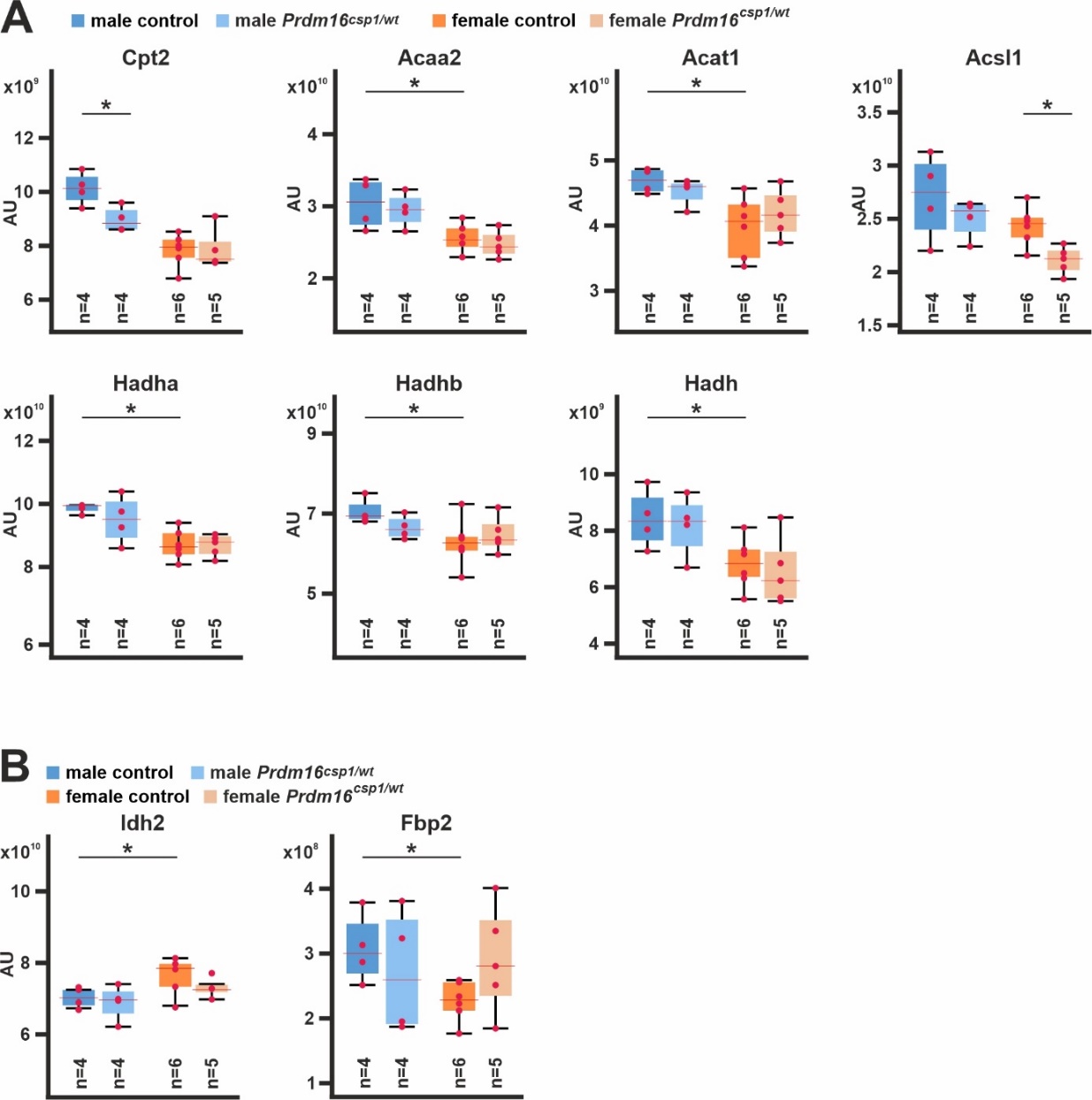


**Figure XII. Maximal capacities of the individual proteins in *Prdm16^csp1/wt^* heart tissue using CARDIOKIN1.** Individual protein impact for **(A)** fatty acid and **(B)** lactate metabolism was calculated for male, female *Prdm16^csp1/wt^* as well as control hearts. Box plots show median values of maximal capacities with 25% quartile. Red dots depict maximal capacities of individual animals. The following significantly altered proteins are presented: Acetyl-CoA acyltransferase 2, mitochondrial (Acaa2); Acetyl-CoA acetyltransferase 1, mitochondrial (Acat1); Acyl-CoA synthetase long-chain family member 1 (Acsl1); Carnitine palmitoyltransferase 2, mitochondrial (Cpt2); Fructose-1,6-bisphosphatase isozyme 2 (Fbp2); Hydroxyacyl-CoA dehydrogenase, mitochondrial (Hadh); Hydroxyacyl-CoA dehydrogenase trifunctional multienzyme complex subunit alpha, mitochondrial (Hadha); Hydroxyacyl-CoA dehydrogenase trifunctional multienzyme complex subunit beta (Hadhb); Isocitrate dehydrogenase 2 (NADP+), mitochondrial (Idh2). Statistical analysis of individual proteins was performed with unpaired t-test, * indicates p<0.05.

Table I: Primers used in this study

| **Gene** | **Transcript** | **Primer** | **Primer sequence** | **Length (nucleotides)** |  |
| --- | --- | --- | --- | --- | --- |
|  |  |  |  |  |  |
| **Primer for genotyping of *Prdm16^csp1/wt^* mice** | | | |  | |
| *Prdm16* | ENSMUSG00000039410 | Prdm16_csp1_ex7_f | TGCACGCATTCCAATCTC | 18 |  |
|  |  | Prdm16_csp1_ex7_r | ATTTCACAGCAGGGTGGAG | 19 |  |
|  |  |  |  |  |  |
| **Primer for molecular genetic analysis of *Prdm16^csp1/wt^* mice** | | | |  | |
| *Prdm16* | ENSMUST00000105638.8 | qmPrdm16_ex3-9_f, qmPrdm16_ex3-4_f | CCCAGGAGAGCTGCATCAAA | 20 |  |
|  |  | qmPrdm16_ex3-9_r | ACTGCGTGTAGGACTTGTGG | 20 |  |
|  |  | qmPrdm16_ex6-8_f | AGCTCTACGAGGGCCTAGG | 19 |  |
|  |  | qmPrdm16_ex6-8_r | GGATGTGACGCTGGAGGTT | 19 |  |
|  |  |  |  |  |  |
| **Primer for cDNA amplification with quantitative PCR** | | | |  | |
| *Actc1* | ENSMUST00000090269.6 | qmActc1_ex2-3_f | CTGGTGAAGGCCGGCTTTGC | 20 |  |
|  |  | qmActc1_ex2-3_r | TCCTTCTGTCCCATACCCACCA | 22 |  |
| *Actn2* | ENSMUST00000064204.13 | qmActn2_ex11-12_f | GCCTCCACGCACGAGACATG | 20 |  |
|  |  | qmActn2_ex11-12_r | TGCTTCCGAAGTAGGGCTCGA | 21 |  |
| *Dsp* | ENSMUST00000124830.2 | qmDsp_ex11-12_f | CGGAACCCAGACTACAGGAGCA | 22 |  |
|  |  | qmDsp_ex11-12_r | GCGCTCGTTGTTGTCCTTCAGA | 22 |  |
| *Gapdh* | [ENSMUST00000118875.7](https://www.ensembl.org/Mus_musculus/Transcript/Summary?db=core;g=ENSMUSG00000057666;r=6:125161715-125166467;t=ENSMUST00000118875) | qmGapdh_f | GGGAAGCCCATCACCATCTT | 20 |  |
|  |  | qmGapdh_r | CGGCCTCACCCCATTTG | 17 |  |
| *Gja5* | ENSMUST00000072600.6 | qmGja5_ex1-2_f | CAGTTGAACAGCAGCCAGAG | 20 |  |
|  |  | qmGja5_ex1-2_r | CTCCAGTCACCCATCTTGCC | 20 |  |
| *Hba-a1* | ENSMUST00000093209.4 | qmHba-a1_ex1-2_f | AGCCCTGGAAAGGATGTTTGCT | 22 |  |
|  |  | qmHba-a1_ex1-2_r | GCGACCTTCTTGCCGTGACC | 20 |  |
| *Hbb-bs* | ENSMUST00000023934.8 | qmHbb-bs_ex2-3_f | GGCCTGAATCACTTGGACAGCC | 22 |  |
|  |  | qmHbb-bs_ex2-3_r | CACGATCATATTGCCCAGGAGCC | 23 |  |
| *Irx4* | ENSMUST00000022095.9 | qmIrx4_ex4-5_f | GCCTACTACCCGTATGAGCC | 20 |  |
|  |  | qmIrx4_ex4-5_r | GTGTGCTGGTGGTCTCCC | 18 |  |
| *Kcna4* | ENSMUSG00000042604 | qmKcna4_ex2_f | AGAAGAAACTCCACCACAGGC | 21 |  |
|  |  | qmKcna4_ex2_r | CTCTTCCTCGCTTAGCTCCC | 20 |  |
| *Myh6* | ENSMUST00000081857.13 | qmMyh6_ex3-4_f | GTTAAGGCCAAGGTCGTGTCCC | 22 |  |
|  |  | qmMyh6_ex3-4_r | ACTTGGGTGGGTTCTGCTGC | 20 |  |
| *Myh7* | ENSMUST00000168485.7 | qmMyh7_ex25-26_f | CCAAGGCCAAGGTCAAGCTGG | 21 |  |
|  |  | qmMyh7_ex25-26_r | GCTTCCGCTTGGCTCGCTC | 19 |  |
| *Myl2* | ENSMUSG00000013936 | qmMyl2_ex3-4_f | ATGGACCAGAACAGAGACGG | 20 |  |
|  |  | qmMyl2_ex3-4_r | CGGTGAAGTTAATTGGACCTGG | 22 |  |
| *Myl3* | ENSMUST00000079784.11 | qmMyl3_ex1-3_f | GGAAGCCGAGTTTGATGCCTCC | 22 |  |
|  |  | qmMyl3_ex1-3_r | TCCCCACACTGCCCGTATGT | 20 |  |
| *Prdm16* | [ENSMUST00000030902.12](https://www.ensembl.org/Mus_musculus/Transcript/Summary?db=core;g=ENSMUSG00000039410;r=4:154316125-154636866;t=ENSMUST00000030902) | qmPrdm16_ex3-4_f | CCCAGGAGAGCTGCATCAAA | 20 |  |
|  |  | qmPrdm16_ex3-4_r | ACGGATGTACTTGAGCCAGC | 20 |  |
|  |  | qmPrdm16_ex6-7_f | AGGATTGCGAGCGGATGTTC | 20 |  |
|  |  | qmPrdm16_ex6-7_r | CCAGTTGAAGGCCTTGGGAC | 20 |  |
|  |  | qmPrdm16_ex7-8_f | GAAGTCCAACCTCATCCGCC | 20 |  |
|  |  | qmPrdm16_ex7-8_r | ATGTGACGCTGGAGGTTGC | 19 |  |
|  |  | qmPrdm16_ex14-15_f | GAACCAGGCATCCACTCGAA | 20 |  |
|  |  | qmPrdm16_ex14-15_r | CTACGTCCTCTGGCTTTGCA | 20 |  |
| *PRDM16* | [ENST00000270722.10](http://www.ensembl.org/Homo_sapiens/Transcript/Summary?db=core;g=ENSG00000142611;r=1:3069168-3438621;t=ENST00000270722) | qhPRDM16_ex3-4_f | CCAGGAAGGCTGCATCACAA | 20 |  |
|  |  | qhPRDM16_ex3-4_r | ACGGATGTACTTGAGCCAGC | 20 |  |
| *Pbxip1* | ENSMUST00000038942.10 | qmPbxip1_ex10-11_f | GGCCCTGAAGAAGAGGTCACGA | 22 |  |
|  |  | qmPbxip1_ex10-11_r | TGGTGGTAGTGGTGCGGATGG | 21 |  |
| *Pyroxd2* | ENSMUST00000076505.4 | qmPyroxd2_ex1-3_f | GAGCAGGACACAATGGGCTGG | 21 |  |
|  |  | qmPyroxd2_ex1-3_r | TCTCCTCCGTGACAGCTGCG | 20 |  |
| *Tcap* | ENSMUST00000008021.2 | qmTcap_ex1-2_f | AACGCAGGGAAGCCTTCTGG | 20 |  |
|  |  | qmTcap_ex1-2_f | GCCGGTGGTAGGTCTCATGC | 20 |  |
| *Tnnt2* | ENSMUST00000112087.8 | qmTnnt2_ex3-4_f | ACTGGAGTGAAGAAGAGGAGGACG | 24 |  |
|  |  | qmTnnt2_ex3-4_fr | GCCTCCTCTGTCTCAGCCTCA | 21 |  |
| *Tpm1* | ENSMUST00000113707.8 | qmTpm1_ex2-3_f | GAAACTGGAGCTGGCGGAGAAA | 22 |  |
|  |  | qmTpm1_ex2-3_r | TCCTGAGCACGATCCAACTCCT | 22 |  |
| *Ttn* | ENSMUST00000111846.8 | qmTtn_ex5-6_f | CACCAACCCCATCTCCAGTCAG | 22 |  |
|  |  | qmTtn_ex5-6_r | GTGGTGGTCTGAGTCTTGCGAAT | 23 |  |

Table II: Primary antibodies used in this study

| **Target protein** | **Producer** | **Antibody ID** | **Origin** |  |
| --- | --- | --- | --- | --- |
|  |  |  |  |  |
| Collagen 1 (Col1) | Abcam | ab21286 | Rabbit IgG |  |
| alpha-smooth muscle actin  (α-SMA) | Sigma-Aldrich | A2547 | Mouse IgG2a |  |
| Tnnt2 | ThermoFisher | MA5-12960 | Mouse IgG1 |  |
|  |  |  |  |  |
|  |  |  |  |  |
|  |  |  |  |  |
|  |  |  |  |  |
|  |  |  |  |  |

Table III: Body composition of *Prdm16^csp1/wt^* mice

|  |  | **male - 8 months** | | |  | **female - 8 months** | | |
| --- | --- | --- | --- | --- | --- | --- | --- | --- |
| **value** | **unit** | *Prdm16^wt/wt^* | *Prdm16^csp1/wt^* | **t-test** |  | *Prdm16^wt/wt^* | *Prdm16^csp1/wt^* | **t-test** |
| **n** |  | 13 | 16 |  |  | 14 | 16 |  |
| **Body weight** | g | 39.26 ± 3.69 | 34.78 ± 1.91 | p ≤ 0.01 |  | 33.59 ± 3.39 | 29.32 ± 2.43 | p ≤ 0.01 |
| **Fat** | % | 26.80 ± 4.79 | 25.64 ± 3.14 | n.s. |  | 30.43 ± 6.27 | 25.42 ± 3.82 | p ≤ 0.01 |
| **Free water** | % | 7.33 ± 0.44 | 7.17 ± 0.41 | n.s. |  | 6.92 ± 0.30 | 6.83 ± 0.40 | n.s. |
| **Muscle tissue** | % | 64.30 ± 5.63 | 64.92 ± 4.26 | n.s. |  | 60.44 ± 6.32 | 65.49 ± 3.83 | p ≤ 0.01 |
|  |  |  |  |  |  |  |  |  |

Statistical significance was calculated by unpaired t-test of *Prdm16^wt/wt^* vs. *Prdm16^csp1/wt^* mice for each sex condition. All values are given as mean ± standard deviation. n.s. - not significant.

Table IV: Body and main echocardiographic parameter of *Prdm16^csp1/wt^* mice.

|  |  | ***Prdm16^csp1/wt^* - male - 8 months** | | |  | ***Prdm16^csp1/wt^* - female - 8 months** | | |
| --- | --- | --- | --- | --- | --- | --- | --- | --- |
| **value** | **unit** | **controls** | ***Prdm16^csp1/wt^*** | **t-test** |  | **controls** | ***Prdm16^csp1/wt^*** | **t-test** |
| **n** |  | 13 | 16 |  |  | 14 | 16 |  |
| **BW** | g | 38.78 ± 2.65 | 34.47 ± 2.12 | p ≤ 0.01 |  | 32.89 ± 3.40 | 28.43 ± 2.46 | p ≤ 0.01 |
| **HW** | g | 0.204 ± 0.029 | 0.169 ± 0.026 | p ≤ 0.01 |  | 0.141 ± 0.009 | 0.128 ± 0.013 | p ≤ 0.01 |
| **TL** | mm | 18.72 ± 0.53 | 18.89 ± 0.52 | n.s. |  | 19.01 ± 0.34 | 19.01 ± 0.25 | n.s. |
| **HW/TL** | g/mm | 0.0109 ± 0.0016 | 0.0090 ± 0.0011 | p ≤ 0.01 |  | 0.0074 ± 0.0004 | 0.0068 ± 0.0006 | p ≤ 0.01 |
| **LVW*** | mg | 155.94 ± 23.12 | 155.16 ± 22.57 | n.s. |  | 132.04 ± 20.25 | 111.43 ± 19.32 | p ≤ 0.01 |
| **LVW/TL*** | mg/mm | 8.35 ± 1.30 | 8.04 ± 0.92 | n.s. |  | 7.08 ± 1.00 | 5.71 ± 0.83 | p ≤ 0.01 |
| **LVIDd*** | mm | 4.57 ± 0.40 | 4.35 ± 0.27 | n.s |  | 3.95 ± 0.28 | 3.86 ± 0.22 | n.s |
| **LVIDs*** | mm | 3.20 ± 0.42 | 2.99 ± 0.32 | n.s. |  | 2.62 ± 0.30 | 2.60 ± 0.21 | n.s |
| **HR*** | bpm | 454.38 ± 33.24 | 443.19 ± 41.45 | n.s. |  | 415.36 ± 58.92 | 402.75 ± 49.05 | n.s |
| **CO*** | ml/min | 17.60 ± 3.27 | 15.05 ± 2.32 | p ≤ 0.05 |  | 13.17 ± 1.50 | 11.19 ± 2.49 | p ≤ 0.05 |
| **FS*** | % | 30.25 ± 3.94 | 31.30 ± 4.64 | n.s. |  | 33.73 ± 3.70 | 32.73 ± 3.07 | n.s |
| **EF*** | % | 60.40 ± 6.66 | 59.74 ± 6.41 | n.s. |  | 65.33 ± 5.09 | 61.55 ± 4.14 | p ≤ 0.05 |
| **FAC*** | % | 53.66 ± 5.13 | 53.49 ± 4.22 | n.s. |  | 59.35 ± 5.65 | 56.67 ± 3.49 | n.s |
| **SV*** | µl | 38.10 ± 7.59 | 34.72 ± 5.42 | n.s. |  | 32.07 ± 4.31 | 27.76 ± 4.88 | p ≤ 0.05 |
|  |  |  |  |  |  |  |  |  |
|  |  |  |  |  |  |  |  |  |

Statistical significance was calculated by unpaired t-test of controls vs. *Prdm16^csp1/wt^* for each sex condition. All values are given as mean ± standard deviation. n.s. - not significant, * - echocardiography. Abbreviations: BW - body weight, HW - heart weight, TL - tibia length, HW/TL - relative heart weight, LVW - left ventricular weight, LVW/BW - relative left ventricular weight normalized to body weight, LVW/TL – relative left ventricular weight normalized to tibia length, LVIDd - left ventricular internal diameter at diastole, LVIDs - left ventricular internal diameter at systole, HR - heart rate, CO - cardiac output, FS - fractional shortening, EF - left ventricle ejection fraction, FAC - fractional area change, SV - stroke volume.

Table V: Echocardiographic characterization of *Prdm16^csp1/wt^* mice

|  |  | ***csp1* - male - 8 months** | | |  | ***csp1* - female - 8 months** | | |
| --- | --- | --- | --- | --- | --- | --- | --- | --- |
| **value** | **unit** | *Prdm16^wt/wt^* | *Prdm16^csp1/wt^* | **t-test** |  | *Prdm16^wt/wt^* | *Prdm16^csp1/wt^* | **t-test** |
| **n** |  | 13 | 16 |  |  | 14 | 16 |  |
| **Vd** | µl | 63.28 ± 11.93 | 58.89 ± 8.28 | n.s. |  | 49.15 ± 4.41 | 45.56 ± 7.15 | n.s. |
| **Vs** | µl | 25.18 ± 7.14 | 23.61 ± 5.85 | n.s. |  | 17.08 ± 2.86 | 17.79 ± 3.50 | n.s. |
| **IVSd** | mm | 0.85 ± 0.10 | 0.89 ± 0.10 | n.s. |  | 0.88 ± 0.12 | 0.81 ± 0.12 | n.s. |
| **IVSs** | mm | 1.19 ± 0.14 | 1.31 ± 0.16 | p ≤ 0.05 |  | 1.22 ± 0.16 | 1.15 ± 0.16 | n.s. |
| **LVPWd** | mm | 0.83 ± 0.10 | 0.88 ± 0.10 | n.s. |  | 0.89 ± 0.11 | 0.80 ± 0.12 | p ≤ 0.05 |
| **LVPWs** | mm | 1.18 ± 0.14 | 1.25 ± 0.14 | n.s. |  | 1.25 ± 0.09 | 1.13 ± 0.15 | p ≤ 0.05 |
| **E** | mm/sec | 829.29 ± 75.25 | 887.11 ± 137.18 | n.s. |  | 782.66 ± 80.69 | 751.03 ± 73.43 | n.s. |
| **A** | mm/sec | 374.00 ± 107.78 | 409.31 ±160.12 | n.s. |  | 306.42 ± 73.53 | 258.34 ± 58.35 | n.s. |
| **E/A** |  | 2.24 ± 0.62 | 2.33 ± 0.67 | n.s. |  | 2.55 ± 0.62 | 2.97 ± 0.50 | n.s. |
| **IVRT** | msec | 16.45 ± 1.74 | 16.64 ± 2.01 | n.s. |  | 16.01 ± 1.86 | 16.93 ± 2.77 | n.s. |
| **IVCT** | msec | 16.88 ± 3.54 | 17.47 ± 3.75 | n.s. |  | 13.40 ± 1.39 | 14.83 ± 1.91 | p ≤ 0.05 |
| **MVD** | msec | 25.60 ± 4.55 | 20.40 ± 4.79 | p ≤ 0.05 |  | 24.72 ± 3.44 | 25.42 ± 3.98 | n.s. |
| **MVET** | msec | 45.85 ± 5.44 | 48.16 ± 4.94 | n.s. |  | 50.40 ± 5.32 | 47.40 ± 5.04 | n.s. |
| **E´** | mm/sec | 30.86 ± 7.42 | 27.54 ± 3.38 | n.s. |  | 29.58 ± 4.06 | 29.88 ± 3.12 | n.s. |
| **A´** | mm/sec | 20.66 ± 3.42 | 15.14 ± 1.78 | p ≤ 0.01 |  | 18.21 ± 3.98 | 16.07 ± 2.46 | n.s. |
| **E´/A´** |  | 1.55 ± 0.41 | 1.90 ± 0.27 | p ≤ 0.05 |  | 1.64 ± 0.28 | 1.88 ± 0.27 | p ≤ 0.05 |
| **E/E´** |  | 28.62 ± 7.06 | 32.54 ± 6.02 | n.s. |  | 26.56 ± 4.13 | 25.39 ± 2.82 | n.s. |
|  |  |  |  |  |  |  |  |  |

Statistical significance was calculated by unpaired t-test of *Prdm16^wt/wt^* vs. *Prdm16^csp1/wt^* mice for each sex condition. All values are given as mean ± standard deviation. n.s. - not significant.

Abbreviations: Vd - volume diastole (trace long axis), Vs - volume systole (trace long axis), IVSd - interventricular septum at diastole, IVSs - interventricular septum at systole, LVPWd - left ventricular posterior wall at diastole, LVPWs - left ventricular posterior wall at systole, E - mitral valve early wave peak velocity flow, A - mitral valve wave peak velocity flow in late, E/A - ratio, IVRT - isovolumic relaxation time, IVCT - isovolumic contraction time, MVD - mitral valve deceleration, MVET - mitral valve ejection time, E´- tissue early diastolic mitral annular velocity, A´ - tissue late diastolic mitral annular velocity

Table VI: Electrocardiography and blood pressure measurements of *Prdm16^csp1/wt^* mice

|  |  | ***csp1* - male - 8 months** | | |  | ***csp1* - female - 8 months** | | |
| --- | --- | --- | --- | --- | --- | --- | --- | --- |
| **value** | **unit** | *Prdm16^wt/wt^* | *Prdm16^csp1/wt^* | **t-test** |  | *Prdm16^wt/wt^* | *Prdm16^csp1/wt^* | **t-test** |
| **n** |  | 13 | 16 |  |  | 14 | 16 |  |
| **HR** | bpm | 664.65 ± 38.49 | 680.12 ± 31.16 | n.s. |  | 667.55 ± 44.51 | 661.53 ± 39.54 | n.s. |
| **RR interval** | msec | 90.60 ± 5.68 | 88.42 ± 3.99 | n.s. |  | 90.46 ± 7.19 | 91.04 ± 5.51 | n.s. |
| **PR interval** | msec | 29.17 ± 2.71 | 29.01 ± 2.39 | n.s. |  | 28.77 ± 1.75 | 28.66 ± 1.29 | n.s. |
| **P interval** | msec | 10.75 ± 1.19 | 11.30 ± 2.18 | n.s. |  | 11.20 ± 1.02 | 10.42 ± 0.54 | p ≤ 0.05 |
| **QRS interval** | msec | 12.35 ± 0.60 | 12.33 ± 0.64 | n.s. |  | 11.91 ± 0.54 | 12.32 ± 0.44 | p ≤ 0.05 |
| **QT interval** | msec | 24.59 ± 1.57 | 25.66 ± 1.45 | n.s. |  | 24.30 ± 1.99 | 25.49 ± 1.34 | n.s. |
| **QT interval (bazett corr)** | msec | 81.82 ± 5.79 | 86.36 ± 5.04 | p ≤ 0.05 |  | 80.90 ± 5.87 | 84.58 ± 4.72 | p ≤ 0.05 |
| **HR*** | bpm | 622.53 ± 36.30 | 613.89 ± 45.69 | n.s. |  | 613.19 ± 43.92 | 599.07 ± 33.41 | n.s. |
| **sBP*** | mmHg | 131.94 ± 11.49 | 128.85 ± 13.53 | n.s. |  | 112.80 ± 11.93 | 116.59 ± 13.96 | n.s. |
| **dBP*** | mmHg | 103.97 ± 10.07 | 99.73 ± 11.37 | n.s. |  | 88.03 ± 8.96 | 89.13 ± 14.46 | n.s. |
| **MAP*** | mmHg | 112.96 ± 10.20 | 109.15 ± 11.78 | n.s. |  | 96.01 ± 9.84 | 97.98 ± 13.93 | n.s. |
|  |  |  |  |  |  |  |  |  |

Statistical significance was calculated by unpaired t-test of *Prdm16^wt/wt^* vs. *Prdm16^csp1/wt^* mice for each sex condition. All values are given as mean ± standard deviation. n.s. - not significant.

Abbreviations: HR - heart rate, * - blood pressure measurements with tail-cuff system, sBP - systolic blood pressure, dBP - diastolic blood pressure, MAP - mean arterial pressure

Table VII: Differential transcript expression of PRDM16 targets in a screen of human DCM heart samples

| **Ensemble gene ID** | **Gene name** | **Adjusted p value** | **Controls RPKM value** | **DCM RPKM value** | **Fold change** | **Differential expression** | **Pubmed ID** | **Type of PRDM16 association** |
| --- | --- | --- | --- | --- | --- | --- | --- | --- |
|  |  |  |  |  |  |  |  |  |
| ENSG00000108602 | *ALDH3A1* | n.d. | n.d. | n.d. | n.d. | n.d. | - | regulated RNAseq *Prdm16^csp1/wt^* |
| ENSG00000108691.5 | *CCL2* | 0,005 | 1,393 | 1,186 | -0,206 | YES | - | regulated RNAseq *Prdm16^csp1/wt^* |
| ENSG00000163814 | *CDCP1* | n.d. | n.d. | n.d. | n.d. | n.d. | - | regulated RNAseq *Prdm16^csp1/wt^* |
| ENSG00000141977 | *CIB3* | n.d. | n.d. | n.d. | n.d. | n.d. | - | regulated RNAseq *Prdm16^csp1/wt^* |
| ENSG00000001626 | *CFTR* | n.d. | n.d. | n.d. | n.d. | n.d. | - | regulated proteome *Prdm16^csp1/wt^* |
| ENSG00000159692.11 | *CTBP1* | 6,36E-14 | 1,182 | 1,129 | -0,053 | NO | 18483224 | physical interaction |
| ENSG00000175029.12 | *CTBP2* | 0,672 | 0,759 | 0,763 | 0,003 | NO | 18483224 | physical interaction |
| ENSG00000245848 | *CEBPA* | n.d. | n.d. | n.d. | n.d. | n.d. | 19641492 | physical interaction |
| ENSG00000172216.4 | *CEBPB* | 2,78E-06 | 1,505 | 1,375 | -0,130 | NO | 19641492 | physical interaction |
| ENSG00000221869.4 | *CEBPD* | 7,18E-05 | 1,582 | 1,393 | -0,190 | YES | 19641492 | physical interaction |
| ENSG00000176194.13 | *CIDEA* | 0,020 | 0,218 | 0,184 | -0,034 | NO | 21123942 | transcriptional target |
| not present in human | *COX8B* | n.d. | n.d. | n.d. | n.d. | n.d. | 21123942 | transcriptional target |
| ENSG00000094963.9 | *FMO2* | 0,249 | 1,100 | 1,146 | 0,046 | NO | - | regulated RNAseq *Prdm16^csp1/wt^* |
| ENSG00000181090.13 | *EHMT1* | 0,004 | 0,539 | 0,558 | 0,019 | NO | 29320702 | physical interaction |
| ENSG00000204371.7 | *EHMT2* | 0,564 | 0,619 | 0,625 | 0,005 | NO | 29320702 | transcriptional complex component |
| ENSG00000119915 | *ELOVL3* | n.d. | n.d. | n.d. | n.d. | n.d. | 21123942 | transcriptional target |
| ENSG00000073712.9 | *FERMT2* | 0,034 | 1,227 | 1,243 | 0,016 | NO | - | regulated RNAseq *Prdm16^csp1/wt^* |
| ENSG00000100116.12 | *GCAT* | 7,22E-11 | 1,279 | 1,157 | -0,122 | NO | - | regulated RNAseq *Prdm16^csp1/wt^* |
| not present in human | *GKN3* | n.d. | n.d. | n.d. | n.d. | n.d. | - | regulated RNAseq *Prdm16^csp1/wt^* |
| ENSG00000174156 | *GSTA3* | n.d. | n.d. | n.d. | n.d. | n.d. | - | regulated RNAseq *Prdm16^csp1/wt^* |
| ENSG00000161509 | *GRIN2C* | n.d. | n.d. | n.d. | n.d. | n.d. | - | regulated RNAseq *Prdm16^csp1/wt^* |
| ENSG00000169181 | *GSG1L* | n.d. | n.d. | n.d. | n.d. | n.d. | - | regulated RNAseq *Prdm16^csp1/wt^* |
| ENSG00000006704.6 | *GTF2IRD1* | 0,026 | 0,651 | 0,611 | -0,040 | NO | 29320702 | physical interaction |
| ENSG00000206172.4 | *HBA1 / Hba-a1* | 0,481 | 0,797 | 0,733 | -0,063 | NO | - | regulated RNAseq *Prdm16^csp1/wt^* |
| not present in human | *HBB-BS* | n.d. | n.d. | n.d. | n.d. | n.d. | - | regulated RNAseq *Prdm16^csp1/wt^* |
| ENSG00000116478.7 | *HDAC1* | 0,220 | 1,099 | 1,092 | -0,007 | NO | 29320702 | transcriptional complex component |
| ENSG00000196591.7 | *HDAC2* | 8,05E-07 | 0,637 | 0,662 | 0,025 | NO | 21516122 | physical interaction |
| ENSG00000171720.5 | *HDAC3* | 0,015 | 0,966 | 0,952 | -0,014 | NO | 29320702 | transcriptional complex component |
| ENSG00000159166.9 | *LAD1* | 0,260 | 0,446 | 0,348 | -0,098 | NO | - | regulated RNAseq *Prdm16^csp1/wt^* |
| ENSG00000009724 | *MASP2* | 4,86E-21 | 0,173 | 0,280 | 0,107 | NO | - | regulated RNAseq *Prdm16^csp1/wt^* |
| ENSG00000125686.7 | *MED1* | 2,48E-25 | 0,606 | 0,794 | 0,189 | YES | 25644604 | physical interaction |
| ENSG00000184634.11 | *MED12* | 3,90E-17 | 0,728 | 0,831 | 0,102 | NO | 29320702 | transcriptional complex component |
| ENSG00000141027.16 | *NCOR1* | 2,48E-25 | 0,701 | 0,847 | 0,147 | NO | 29320702 | transcriptional complex component |
| ENSG00000196498.9 | *NCOR2* | 7,70E-05 | 1,043 | 1,082 | 0,039 | NO | 29320702 | transcriptional complex component |
| ENSG00000077009.9 | *NMRK2* | 0,017 | 2,542 | 2,466 | -0,076 | NO | - | regulated RNAseq *Prdm16^csp1/wt^* |
| ENSG00000126368.5 | *NR1D1* | 0,414 | 1,050 | 1,022 | -0,028 | NO | - | regulated RNAseq *Prdm16^csp1/wt^* |
| ENSG00000163346.12 | *PBXIP1* | 0,087 | 1,399 | 1,388 | -0,012 | NO | - | regulated RNAseq, proteome *Prdm16^csp1/wt^* |
| ENSG00000085276.13 | *PRDM3 (MECOM)* | 1,20E-06 | 0,542 | 0,618 | 0,075 | NO | 30462309 | physical interaction |
| ENSG00000142611.12 | *PRDM16 (MEL1)* | 9,98E-05 | 0,500 | 0,552 | 0,052 | NO | - | physical interaction, regulated RNAseq, |
| ENSG00000186951.12 | *PPARA* | 3,55E-21 | 0,897 | 1,087 | 0,191 | YES | 18719582 | physical interaction |
| ENSG00000132170.15 | *PPARG* | 0,002 | 0,659 | 0,618 | -0,041 | NO | 18719582 | physical interaction |
| ENSG00000109819.4 | *PPARGC1A* | 1,09E-12 | 0,971 | 1,116 | 0,145 | NO | 18483224 17618855 | physical interaction |
| ENSG00000155846.12 | *PPARGC1B* | 5,61E-11 | 0,553 | 0,706 | 0,152 | NO | 18483224 17618855 | physical interaction |
| ENSG00000119943.6 | *PYROXD2* | 0,266 | 0,735 | 0,755 | 0,019 | NO | - | regulated RNAseq, proteome *Prdm16^csp1/wt^* |
| ENSG00000162521.14 | *RBBP4* | 1,99E-07 | 0,881 | 0,931 | 0,049 | NO | 30462309 | physical interaction |
| ENSG00000080839.7 | *RBL1* | 4,28E-09 | 0,240 | 0,288 | 0,048 | NO | 29320702 | transcriptional complex component |
| ENSG00000089902.8 | *RCOR1* | 7,74E-17 | 0,648 | 0,720 | 0,072 | NO | 29320702 | transcriptional complex component |
| ENSG00000167771.5 | *RCOR2* | 0,178 | 0,432 | 0,413 | -0,018 | NO | 29320702 | transcriptional complex component |
| ENSG00000149489.4 | *ROM1* | 0,0004 | 0,253 | 0,219 | -0,035 | NO | - | regulated RNAseq *Prdm16^csp1/wt^* |
| not present in human | *SCD4* | n.d. | n.d. | n.d. | n.d. | n.d. | - | regulated RNAseq *Prdm16^csp1/wt^* |
| ENSG00000157933.9 | *SKI* | 0,528 | 1,117 | 1,125 | 0,008 | NO | 19049980 | physical interaction |
| ENSG00000175387.11 | *SMAD2* | 3,02E-14 | 0,690 | 0,748 | 0,057 | NO | 19049980 | physical interaction |
| ENSG00000166949.11 | *SMAD3* | 0,115 | 0,755 | 0,760 | 0,005 | NO | 17467076 19049980 | physical interaction |
| ENSG00000124664 | *SPDEF* | n.d. | n.d. | n.d. | n.d. | n.d. | 26186194 28514442 | physical interaction |
| ENSG00000159674.7 | *SPON2* | 2,95E-06 | 0,681 | 0,595 | -0,086 | NO | - | regulated RNAseq *Prdm16^csp1/wt^* |
| ENSG00000099956.13 | *SMARCB1 (SNF5)* | 1,22E-05 | 1,230 | 1,191 | -0,040 | NO | 29320702 | transcriptional complex component |
| ENSG00000145335.11 | *SNCA* | 2,99E-10 | 0,385 | 0,512 | 0,127 | NO | - | regulated RNAseq *Prdm16^csp1/wt^* |
| ENSG00000157152 | *SYN2* | n.d. | n.d. | n.d. | n.d. | n.d. | - | regulated RNAseq *Prdm16^csp1/wt^* |
| ENSG00000198270.8 | *TMEM116* | 0,017 | 0,766 | 0,808 | 0,042 | NO | - | regulated RNAseq *Prdm16^csp1/wt^* |
| ENSG00000090447 | *TFAP4* | n.d. | n.d. | n.d. | n.d. | n.d. | 19505873 | physical interaction |
| ENSG00000120942.9 | *UBIAD1* | 1,94E-05 | 0,605 | 0,627 | 0,0223 | NO | - | regulated RNAseq *Prdm16^csp1/wt^* |
| ENSG00000162543 | *UBXN10* | n.d. | n.d. | n.d. | n.d. | n.d. | - | regulated RNAseq *Prdm16^csp1/wt^* |
| ENSG00000109424 | *UCP1* | n.d. | n.d. | n.d. | n.d. | n.d. | 21123942 25644605 | transcriptional target |
| ENSG00000101493.6 | *ZNF516* | 3,88E-13 | 0,499 | 0,570 | 0,071 | NO | 25578880 | physical interaction |

RPKM values - reads per kilobase of transcript per million mapped reads, n.d. - not determined. Data originate from a Heinig et al. 2017. The Top5 regulated genes from *Prdm16^csp1/wt^* hearts identified by the RNAseq and the proteome screen were analyzed in the transcriptome data of Heinig et al. 2017. In the study by Heinig et al. 2007 differential expression was not observed (NO) or differential expression was observed (YES).

Table VIII: Metabolite analysis of *Prdm16^csp1/wt^* heart tissue

|  |  | ***csp1* - male - 8 months** | | |  | ***csp1* - female - 8 months** | | |
| --- | --- | --- | --- | --- | --- | --- | --- | --- |
| **value** | **unit** | *Prdm16^wt/wt^* | *Prdm16^csp1/wt^* | **t-test** |  | *Prdm16^wt/wt^* | *Prdm16^csp1/wt^* | **t-test** |
| **n** |  | 4 | 6 |  |  | 6 | 6 |  |
| **ATP** | nAr. | 0.038 ± 0.010 | 0.039 ± 0.007 | n.s. |  | 0.026 ± 0.007 | 0.020 ± 0.006 | n.s. |
| **ADP** | nAr. | 0.317 ± 0.065 | 0.224 ± 0.043 | n.s. |  | 0.205 ± 0.019 | 0.179 ± 0.017 | p ≤ 0.05 |
| **AMP** | nAr. | 4.22 ± 0.08 | 3.57 ± 0.39 | p ≤ 0.05 |  | 3.55 ± 0.27 | 3.46 ± 0.20 | n.s. |
| **IMP** | nAr. | 0.113 ± 0.019 | 0.231 ± 0.040 | p ≤ 0.05 |  | 0.242 ± 0.039 | 0.326 ± 0.054 | p ≤ 0.05 |
| **Hypoxanthine** | nAr. | 0.719 ± 0.045 | 1.002 ± 0.132 | p ≤ 0.05 |  | 1.018 ± 0.084 | 0.993 ± 0.144 | n.s. |
| **[ADP/ATP]** |  | 7.05 ± 1.61 | 6.23 ± 0.88 | n.s. |  | 7.69 ± 2.03 | 8.86 ± 1.65 | n.s. |
| **[AMP/ATP]** |  | 116.5 ± 25.3 | 100.5 ± 11.1 | n.s. |  | 123.4 ± 39.6 | 174.3 ± 59.7 | n.s. |
| **[IMP/ATP]** |  | 2.61 ± 0.89 | 6.85 ± 2.12 | p ≤ 0.05 |  | 8.32 ± 3.14 | 12.60 ± 2.73 | n.s. |
| **Creatine** | nAr. | 43.97 ± 0.96 | 48.65 ± 3.06 | p ≤ 0.05 |  | 46.32 ± 2.47 | 46.74 ± 2.99 | n.s. |
| **Creatine-P** | nAr. | n.d. | n.d. |  |  | 0.053 ± 0.017 | 0.041 ± 0.014 | n.s. |
| **[Creatine-P/ Creatine]** |  | n.d. | n.d. |  |  | 0.0011 ± 0.0004 | 0.0007 ± 0.0001 | n.s. |
| **[Creatine-P/ATP]** |  | n.d. | n.d. |  |  | 1.722 ± 0.332 | 1.758 ± 0.434 | n.s. |
| **Acetyl-CoA** | nAr. | 0.0087 ± 0.0008 | 0.0070 ± 0.0008 | p ≤ 0.05 |  | 0.0062 ± 0.0014 | 0.0068 ± 0.0008 | n.s. |
| ***NAD+** | nAr. | 1.47 ± 0.15 | 1.54 ± 0.17 | n.s. |  | 1.38 ± 0.12 | 1.29 ± 0.08 | n.s. |
| ***NADH** | nAr. | 0.0012 ± 0.0003 | 0.0017 ± 0.0007 | n.s. |  | 0.0017 ± 0.0004 | 0.0030 ± 0.0011 | p ≤ 0.05 |
| ***[NADH/NAD+]** |  | 0.0008 ± 0.0002 | 0.0011 ± 0.0004 | n.s. |  | 0.0012 ± 0.0003 | 0.0023 ± 0.0008 | p ≤ 0.05 |
|  |  |  |  |  |  |  |  |  |
|  |  |  |  |  |  |  |  |  |
| ***NADP+** | nAr. | 0.0966 ± 0.0090 | 0.1020 ± 0.0144 | n.s. |  | 0.0979 ± 0.0149 | 0.0927 ± 0.0043 | n.s. |
| ***NADPH** | nAr. | 0.00005 ± 0.00002 | 0.00093 ± 0.00043 | p ≤ 0.05 |  | 0.00052 ± 0.00033 | 0.00030 ± 0.00012 | n.s. |
| ***[NADPH/NADP+]** |  | 0.00054 ± 0.00021 | 0.00877 ± 0.00390 | p ≤ 0.05 |  | 0.00521 ± 0.00319 | 0.00332 ± 0.00134 | n.s. |
| ***FAD+** | nAr. | n.d. | n.d. |  |  | 0.097 ± 0.005 | 0.086 ± 0.011 | n.s. |
| ***FADH2** | nAr. | n.d. | n.d. |  |  | 0.0050 ± 0.0003 | 0.0064 ± 0.0006 | p ≤ 0.05 |
| ***FADH2/FAD+** |  | n.d. | n.d. |  |  | 0.0522 ± 0.0052 | 0.0753 ± 0.0082 | p ≤ 0.05 |
| ***GSSG** | nAr. | 0.7624 ± 0.0934 | 0.8153 ± 0.0623 | n.s. |  | 0.6831 ± 0.0659 | 0.6262 ± 0.0234 | n.s. |
| ***GSH** | nAr. | 0.0145 ± 0.0028 | 0.0168 ± 0.0030 | n.s. |  | 0.0115 ± 0.0024 | 0.0075 ± 0.0007 | p ≤ 0.05 |
| ***[GSH/GSSG]** |  | 0.0188 ± 0.0018 | 0.0217 ± 0.0010 | n.s. |  | 0.0167 ± 0.0025 | 0.0120 ± 0.0010 | p ≤ 0.05 |
| **4-Hydroxynonenal** | nAr. | 0.0340 ± 0.0078 | 0.0335 ± 0.0048 | n.s. |  | 0.0351 ± 0.0028 | 0.0361 ± 0.0060 | n.s. |

Statistical significance was calculated by unpaired t-test of *Prdm16^wt/wt^* vs. *Prdm16^csp1/wt^* mice for each sex condition. All values show normalized peak area (nAr.) and given as mean ± standard deviation. n.s. - not significant. * - measurement applying an electrospray ionization interface operated in positive ionization mode only with the m/z range of 200-1500 amu.

Abbreviations: ATP - adenosine triphosphate, ADP - adenosine diphosphate, AMP - adenosine monophosphate, IMP - inosine monophosphat, NAD+ - nicotinamide adenine dinucleotide oxidized, NADH - nicotinamide adenine dinucleotide reduced, NADP+ - nicotinamide adenine dinucleotide phosphate oxidized, NADPH - nicotinamide adenine dinucleotide phosphate reduced, FAD+ - flavin adenine dinucleotide oxidized, FADH2 - flavin adenine dinucleotide reduced, GSH - glutathione reduced, GSSG - glutathione oxidized

Table IX: Eicosanoid analysis of *Prdm16^csp1/wt^* heart tissue

|  |  | ***csp1* - male - 8 months** | | |  |
| --- | --- | --- | --- | --- | --- |
| **value** | **unit** | *Prdm16^wt/wt^* | *Prdm16^csp1/wt^* | **t-test** | **enzyme + substrate** |
| **n** |  | 7 | 6 |  |  |
| **5-HETE** | ng/g | 240.7 ± 62.3 | 348.2 ± 105.6 | n.s. | LOX + ARA |
| **8-HETE** | ng/g | 220.6 ± 72.4 | 187.2 ± 55.3 | n.s. | LOX + ARA |
| **11-HETE** | ng/g | 153.4 ± 67.2 | 230.6 ± 127.5 | n.s. | LOX + ARA |
| **12-HETE** | ng/g | 403.1 ± 138.0 | 306.1 ± 130.2 | n.s. | LOX + ARA |
| **15-HETE** | ng/g | 1451.1 ± 546.4 | 1221.9 ± 773.1 | n.s. | LOX + ARA |
| **20-HETE** | ng/g | 3.26 ± 0.75 | 2.69 ± 1.19 | n.s. | CYP + ARA |
| **17,18-DiHETE** | ng/g | 0.72 ± 0.05 | 0.83 ± 0.20 | n.s. | CYP + ARA |
| **5,6-EET** | ng/g | 1560.0 ± 862.6 | 3836.9 ± 1827.6 | p ≤ 0.05 | CYP + ARA |
| **8,9-EET** | ng/g | 922.1 ± 474.9 | 1958.5 ± 830.8 | p ≤ 0.05 | CYP + ARA |
| **11,12-EET** | ng/g | 1515.6 ± 649.0 | 2526.9 ± 916.6 | p ≤ 0.05 | CYP + ARA |
| **14,15-EET** | ng/g | 1040.1 ± 544.7 | 2629.5 ± 1218.6 | p ≤ 0.05 | CYP + ARA |
| **EET_sum** | ng/g | 5037.9 ± 2465.4 | 10951.7 ± 4769.6 | p ≤ 0.05 | - |
| **5,6-DHET** | ng/g | 5.76 ± 1.78 | 10.26 ± 2.89 | p ≤ 0.05 | CYP + ARA |
| **8,9-DHET** | ng/g | 2.15 ± 0.62 | 2.49 ± 0.99 | n.s. | CYP + ARA |
| **11,12-DHET** | ng/g | 1.95 ± 0.33 | 2.32 ± 0.97 | n.s. | CYP + ARA |
| **14,15-DHET** | ng/g | 1.58 ± 0.65 | 3.73 ± 2.55 | n.s. | CYP + ARA |
| **DHET_sum** | ng/g | 11.43 ± 2.24 | 18.18 ± 3.97 | p ≤ 0.01 | - |
| **19,20-DiHDPA** | ng/g | 43.4 ± 20.1 | 83.5 ± 34.5 | p ≤ 0.05 | CYP + DHA |
| **19,20-EDP** | ng/g | 3817.6 ± 1954.4 | 6759.0 ± 1779.5 | p ≤ 0.05 | CYP + DHA |
| **17,18-EEQ** | ng/g | 6.62 ± 4.32 | 9.40 ± 4.57 | n.s. | CYP + EPA |
|  |  |  |  |  |  |

Statistical significance was calculated by unpaired t-test of male *Prdm16^wt/wt^* vs. *Prdm16^csp1/wt^* mice. All values are given as mean ± standard deviation. n.s. - not significant.

Abbreviations: ARA - arachidonic acid, CYP - cytochrome P450 enzymes, DHA - docosahexaenoic acid, DHET - dihydroxy eicosatrienoic acids, DiHDPA - dihydroxydocosapentaenoic acid, DiHETE - dihydroxyeicosatetraenoic acid, EDP - epoxydocosapentaenoic acid, EET - epoxyeicosatrienoic acid, EEQ - epoxyeicosatetraenoic acid, EPA - eicosapentaenoic acid, HETE - hydroxyeicosatetraenoic acid, LOX - lipoxygenases
